# Facilitating and Inhibiting Factors in the Design, Implementation, and Applicability of Value-Based Payment Models: A Systematic Literature Review

**DOI:** 10.1177/10775587231160920

**Published:** 2023-03-23

**Authors:** Diogo L. L. Leao, Henricus-Paul Cremers, Dennis van Veghel, Milena Pavlova, Frederique J. Hafkamp, Wim N. J. Groot

**Affiliations:** 1 Maastricht University, The Netherlands; 2 Maastro Clinic, Maastricht, The Netherlands; 3 Netherlands Heart Network, Eindhoven, The Netherlands

**Keywords:** payment reform, value-based health care, value-based payment models, alternative payment models, value

## Abstract

Evidence on the potential for value-based payment models to improve quality of care and ensure more efficient outcomes is limited and mixed. We aim to identify the factors that enhance or inhibit the design, implementation, and application of these models through a systematic literature review. We used the PRISMA guidelines. The facilitating and inhibiting factors were divided into subcategories according to a theoretical framework. We included 143 publications, each reporting multiple factors. Facilitators on objectives and strategies, such as realistic/achievable targets, are reported in 56 studies. Barriers regarding dedicated time and resources (e.g., an excessive amount of time for improvements to manifest) are reported in 25 studies. Consensus within the network regarding objectives and strategies, trust, and good coordination is essential. Health care staff needs to be kept motivated, well-informed, and actively involved. In addition, stakeholders should manage expectations regarding when results are expected to be achieved.

## Introduction

Value-based health care (VBHC) primarily aims to improve value for patients, defined as improving health outcomes against equal or lower costs. One way to achieve this is to make the reimbursement of providers partly or fully dependent on achieved patient value (outcomes/costs) (Porter, 2009), known as value-based payment (VBP). To guide health systems to accomplish high-quality, value-based care, the Quadruple Aim serves as a guiding principle. The four elements to focus on are improving patient experience, improving health of populations, reducing cost of care, and improving provider experience (Cattel & Eijkenaar, 2020; Iglesia et al., 2020). To contribute to the evidence on the effectiveness of VBP models, this review focuses not only on factors that enhance these models in any of the four focus elements of the Quadruple Aim but also on hindering factors that should be avoided.

The Health Care Payment Learning & Action Network (HCPLAN) was developed to align payment models across the public and private sectors. For this, an Alternative Payment Model (APM) Framework (2017) was created. This framework is composed of four categories. The first relates to fee-for-service models not linked to quality or value. These are the models for which VBP was developed to substitute. Category 2 refers to fee-for-service models linked to quality and value, whereas Category 3 refers to alternative payment models that build on the fee-for-service architecture. According to Damberg et al. (2014), VBP mostly refers to pay-for-performance (P4P) and bundled-payment schemes. These models are integrated into Categories 2 and 3 of the APM framework, respectively. In P4P models, health care providers receive financial incentives for achieving targets on performance measures previously defined (Eijkenaar, 2012). The improvement and/or achievement of these targets may be value-related (both costs and outcomes; Roland & Campbell, 2014), but may also solely regard quality indicators (Pay-for-Quality; Busse et al., 2019). Depending on the arrangement, providers may have to pay penalties if targets are not met (Cromwell et al., 2007). Bundled-payment schemes are integrated into Category 3 of the APM framework if they relate to clinical episode payments. In these schemes, providers are held accountable for the quality and cost of care delivered during a certain episode. Providers may benefit from shared savings if spending is within a risk-adjusted budget and may incur shared losses if the budget is exceeded (Mechanic et al., 2011). This may provide financial motivation for health care professionals to coordinate care during the entire episode (Mechanic, 2015).

In the United States, the implementation of VBP models has been growing exponentially (Fisher & Shortell, 2010). This is done, among others, through accountable care organizations (ACOs). ACOs refer to health care organizations composed of physicians, hospitals, and other health care providers who work together to deliver coordinated care to patients. This payment model ties provider reimbursement to performance on quality measures and reductions in the cost of care. Providers in the ACO agree to accept a financial risk and are eligible for a share of the cost savings achieved through improved care delivery if they achieve quality and spending targets that are negotiated between the ACO and the payer (Damberg et al., 2014). According to the HCPLAN, most ACO arrangements can be placed in Category 3A or 3B, depending on whether the risk arrangement includes only upside share savings or both upside shared savings and downside risk for providers (penalties may be applied), respectively (APM Framework, 2017). Category 4 of the APM framework includes population-based payment models. In these schemes, the financial risk is fully placed on the health care providers. In our study, we focused on models in which both health care providers and the payment agency (insurance companies) share in the financial risk. Therefore, we exclude models belonging to Categories 1 and 4 of the APM framework.

Despite the increased interest and uptake of VBP models, evidence of their potential to improve quality of care and efficiency outcomes is mixed and limited (Damberg et al., 2014; Eijkenaar et al., 2013; Jha et al., 2012; Lindenauer et al., 2007; Mehrotra et al., 2009; Rosenthal & Frank, 2006; Ryan, 2009; Ryan et al., 2012). The limited evidence on impact might be due to context, among others. As indicated by Porter and Lee (2013), two of the six pillars to move toward a high-value health care delivery system are the integration of care across separate facilities and expanding geographic reach (Porter & Lee, 2013). As VBP models with a focus on only one provider might create incentives for the optimization of only part of the total patient pathway delivered by the involved provider, this might hinder patient-centeredness and the integration of care pathways between providers. To address this, VBP models are frequently applied within networks of care (NOC). These are groups of health service delivery sites, connected across all levels of care that share responsibility for health outcomes (Carmone et al., 2020). In addition, VBP models are also regularly applied within transmural care. Transmural care is a type of care provided based on collaboration and coordination between different levels of care such as primary and secondary care (Bloemen-Vrencken et al., 2005). As NOC implies a connection between health center, and transmural care connects primary to tertiary care, these concepts partly overlap but are also complementary. We found it important to incorporate both terms as the integration of care can be between any combination of primary, secondary, and tertiary care. In both NOC and transmural care, multidisciplinary teams of care providers from different levels of care and/or health service delivery sites work together, with an emphasis on collaboration. Having the same indicators across all sites could lead not only to cost savings but also, and above all, to improved quality of care (Porter et al., 2016). The complexity that is introduced by this collaboration provides a barrier to the implementation of VBP models, further hindered by the dependence on the institutional setting and the overall context (Conrad et al., 2014). This complexity might be a reason for the lack of consistent positive results from the implementation of these payment models. Analyzing the factors that hinder or enhance the quality of VBP models can help the broader implementation of these models.

Previous reviews have mostly focused on the design and effects of P4P models as well as on the complexity of the models and subsequent need for tailoring to the specific setting of implementation (Eijkenaar, 2013). There have also been reviews on the economic evaluation of P4P and on the effects of monetary incentives for physician groups (Heider & Mang, 2020). The variables of VBP models that influence costs of NOC in the United States have also been reviewed (Chee et al., 2016). What is lacking is a review of the evidence on all the factors that influence VBP models, not only within a context of NOC and transmural care, but also not restricted to one single country. Such evidence may contribute to the development of new and more effective VBP models.

### New Contribution

This systematic literature review aims at contributing to the literature by providing an overview of the factors that facilitate or inhibit the successful development, implementation, and applicability of VBP models in the context of NOC and transmural care context. We also aim to make this overview without restricting our search to any specific countries. With this viewpoint, we expect organizations to take this overview into account when planning to adopt VBP models, gain some knowledge on the factors to support and the ones to avoid, and ultimately create value to their patients. In addition, we decided to take an organizational perspective in this study, rather than a policy evaluation perspective. Our objective was to focus on elements that hospital stakeholders are familiar with and can also change, improve on, and/or avoid. We did focus on the context outside the institutions, which involves the inclusion of external stakeholders such as policy makers.

### Theory

Greenhalgh et al. (2004) published a multilevel framework for studying diffusion of innovations in health care based on a cross-disciplinary systematic literature review. This framework was built to better organize the determinants of diffusion, dissemination, and implementation of innovations in health service delivery, considering the complexity of the situation and its several interactions. Greenhalgh et al. (2004) proposed this model to facilitate and guide a process of critical thinking about the complex aspects of the innovation and the system in which it is introduced and how these aspects might interact as adoption occurs.

The concept of VBHC has been discussed for over a decade. However, its optimal implementation is a topic of ongoing discussion and may be considered an innovation. Porter and Lee (2013) refer to a need for a completely new strategy when introducing VBHC (Porter & Lee, 2013). Ramos et al. (2021) also consider VBHC an innovation, as multiple, complex systems are present, with several actors influencing each other and others, and blurry boundaries. We aim to look back at what research has been conducted and contribute to innovative ways of implementing VBHC. When introducing VBP models, there may be requirements such as organizational changes, process changes, new policies, or new mechanisms. Facilitating and inhibiting factors of VBP models are critical aspects that affect the effectiveness of these innovative payment models. Hence, to further analyze the facilitators and inhibitors of VBP models, we adapt the framework proposed by Greenhalgh et al. (2004) .

**Figure 1.**
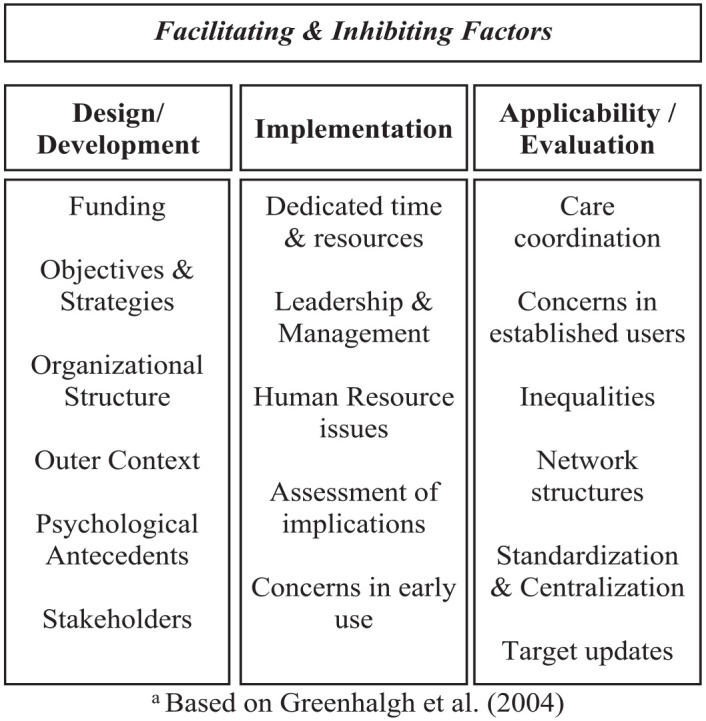
Framework of Facilitating and Inhibiting Factors of VBP Models *Note.* Based on Greenhalgh et al. (2004). VBP = value-based payment.

We only extract and keep framework items relevant to the topic of our review and add new ones. Greenhalgh’s model encompasses the design and implementation as two important stages in building strong links among the determinants of diffusion, dissemination, and implementation of innovations. We take these two stages as important elements in the introduction of VBP models, and add their applicability and evaluation, related to when the model is already functioning/being applied. Therefore, our framework (see Figure 1) divides the facilitating and inhibiting factors into three main categories: development/design, implementation, and applicability/evaluation. These three distinguishable stages may have different facilitating and inhibiting factors associated with them. Making such a division may help differentiate these factors by each stage of the introduction of VBP models. To deepen the framework and systematically organize the factors found in the included publications, we divide these three categories into subcategories.

Outer context and psychological antecedents are two elements included in Greenhalgh’s model, which we find important to include in the design and development stage of our framework, the former relating to the outside elements and the latter relating to the understanding of VBP models. In addition, funding and organizational structure are two elements included in other components of Greenhalgh’s model, which are important to single out and include in a framework for such complex models. Although objectives and strategies, and stakeholders are not directly mentioned by Greenhalgh and colleagues, the conceptual model mentions the importance of clear goals and priorities as well as the involvement of several actors, namely, leaders and managers, which are also vital when introducing VBP models. The implementation stage of our framework is formed by the subcategories: dedicated time and resources, leadership and management, human resource issues, assessment of implications, and concerns in early use. All these subcategories are present, either as elements or as components of elements, in Greenhalgh’s model. We consider that resources such as finances, time, knowledge, and leadership are of great importance when implementing VBP models and are warranted separate categories. There is a wide range of resources, and several different factors can be differentiated and allocated separately, so that conclusions can be taken. In addition, we believe assessing the possible effects of VBP models and any concerns in their early use is also important to consider for success in implementing such models. In the final stage, the applicability and evaluation, we allocated the elements of concerns of established users and network structures from Greenhalgh’s model, to consider any concerns that arise from the application of VBP models as well as any factors that may influence the NOC that involve such models (an important part of our study focus), respectively. In addition, care coordination is considered by the team to be one of the most important elements in the whole framework as it encompasses any factors related to patient care and the coordination expected from adopting VBP models. The standardization and centralization of resources, services, and training are also warranted a separate category, as well as any inequalities that may arise or be solved by applying VBP models.

There may be some overlap when allocating each factor to a category and subcategory, as factors do not belong purely to a single category. However, to make an overview of facilitating and inhibiting factors and take conclusions, there needs to be allocation. The primary researcher builds the framework, and its elements are discussed among the team, until a consensus is reached.

## Methods

As systematic reviews have become progressively important in health care, it is vital to ensure clarity in reporting what is done in the study and what is found (Moher et al., 2009). To achieve this, we applied the Preferred Reporting Items for Systematic Reviews and Meta-Analyses (PRISMA) flowchart and checklists (Supplemental Materials). The flow diagram includes the number of records that were screened and eligibility criteria applied as well as the number of articles that remained to be included in the study. As for the checklists, one outlines the items to include when reporting a systematic literature review, while the other outlines the items to include in the abstract of the study. Prior to the review, the review protocol was registered in Prospero (ID CRD42021259630).

Inclusion criteria are the following:

- Type of payment model—Value-based payment models under Categories 2 and 3 of the APM framework. Category 2 refers to fee-for-service with a link to quality and value, such as P4P models. Category 3 refers to alternative payment models built on fee-for-service architecture, such as shared savings models (with and without downside risk);
- Type of care—Transmural care or care provided in a network of providers;
- Setting—Networks of care, multiple providers, or any other setting that includes more than one provider;
- Analysis of the payment model—Outlining barriers and/or success factors of VBP models.

Exclusion criteria are the following:

- Type of payment model—Non-VBP models such as FFS were excluded. As we only included shared risk models, we also excluded global/budget/population-based payment models such as capitation schemes described in the APM framework under Category 4. Studies not covering payment models at all were also excluded;
- Type of care—Not mentioning transmural care, which means only referring to primary/secondary/tertiary care singularly, and not combined;
- Setting—Single providers (only one organization);
- Analysis of the payment model—Not mentioning barriers and/or success factors of VBP models.

For a study to be included in this systematic literature review, it had to fulfill all four inclusion criteria simultaneously. If a study met one or more exclusion criteria, it was excluded. By doing so, we ensured all studies were aligned with our study aim.

### Search Strategy

The search was conducted in July 2021 in PubMed, PsycINFO, Cochrane Library, JSTOR, EconLit, CINAHL, PsycArticles, and Trip Database.

Three key components were used to build the search terms for the identification of studies on the facilitators and barriers of VBP models: (a) keywords related to VBHC (e.g., P4P or bundled); (b) keywords related to provider payment (e.g., incentive or model); and (c) keywords related to transmural and NOC (e.g., multiple providers or intramural). Synonyms of those keywords and differences in spelling were considered when deciding on the exact search phrase. MeSH terms were also included, when applicable, to ensure comprehensiveness. In addition, the search was limited to publications from January 2005 onward and written in English. We selected this starting period as, based on PubMed searches, VBHC seems to have gained a lot of traction around 2005, with the example of Michael Porter’s book on the redefinition of health care being published in 2006 (Porter, 2006). The search was conducted based on keywords present in the title and/or abstract (Supplemental Materials). The search phrase used in each database can be found in Supplemental Materials.

### Study Selection Process

The selection process followed three steps: screening based on title and abstract, screening based on full text, and screening of reference lists. The primary researcher was the main screener in both the title/abstract and full-text screenings. In the title and abstract screening, the primary screener reviewed all titles and abstracts, and determined a study’s eligibility for the next full-text screening. The inclusion criteria required that the study examined a VBP model and NOC or transmural care. Two other researchers (additional screeners) cross-checked one third of studies that the primary researcher deems “excluded” and all studies “uncertain.” If all screeners could not determine if a study met the inclusion criteria, the study was included for full-text screening.

Three screeners, including the primary researcher, participated in the full-text screening. To ensure consistency, the same percentage of studies “excluded” by the primary screener (one third) and all “uncertain” studies were cross-checked by the two additional screeners. In addition to the inclusion criteria used in the first screening, studies were deemed eligible only if they also focused on facilitators/inhibitors of VBP models. Also, the studies should be peer-reviewed. Commentaries, briefs, essays, reviews, and any non-peer-reviewed publications were excluded. Finally, articles for which the full text cannot not be found were also excluded, after attempting to contact the authors and the journal in which they were published.

After the two stages of screening, the final selection included publications of VBP models in NOC and transmural care and outlining facilitating and/or inhibiting factors of these models. We screened the reference lists of all included publications. During the screening of the reference lists, potentially relevant titles that mention VBP models were included for a check. The main researcher screened the title/abstract and full text using the same inclusion criteria as mentioned before. The included articles were added to the final selection of publications for analysis. Commentaries, briefs, essays, reviews, and any non-peer-reviewed publications, with all other inclusion criteria fulfilled, were once again excluded.

Cohen’s Kappa was used to measure the interrater reliability between the primary researcher and the two additional screeners (Supplemental Materials). In both title and abstract screening (κ = 0.984), and full-text screening (κ = 0.853), there was almost perfect agreement.

### Quality Assessment

The quality of the quantitative studies was assessed using the “Quality Assessment Tool for Quantitative Studies” by the Effective Public Health Practice Project (EPHPP). The Critical Appraisal Skills Program (CASP) checklist was used to assess the qualitative studies. The quality of the included studies was evaluated as strong, moderate, or weak using these two tools. If a quantitative study had no aspects rated as weak in any sections that composed the EPHPP assessment tool, it was considered a study with high quality (strong). A qualitative study was rated as strong if at least six of nine study questions in the CASP assessment tool were evaluated positively (if the answer was “Yes”). Mixed methods studies were assessed based on both checklists mentioned above. The primary researcher carried out the quality assessment.

### Data Extraction and Analysis

After the screening, categories relevant to the review were identified, and information was extracted based on those categories. This directed qualitative content analysis was based on Hsieh and Shannon (2005). The key results were presented narratively and in tables. In addition to information on facilitating and inhibiting factors of VBP models under NOC and transmural care, we also extracted data on the VBP models studied and the outcomes of these models. The themes that formed the units of analysis were (a) general description of the publications and (b) facilitating and inhibiting factors in the design/development, implementation, and applicability of the VBP models studied.

For each included article, facilitating and inhibiting factors were extracted by the primary researcher. Each factor was allocated to one of the three categories formulated and, afterward, to one of the subcategories. After the totality of included publications was analyzed, and factors were allocated, the rest of the author team confirmed the classification made. Any differences in the classification were discussed between all authors until a consensus was reached. Tables, including factors and allocation to categories and subcategories, were built and filled by the primary author (Tables 2 to 4). The rest of the team confirmed their correct classification and allocation. To find each factor in the included publications, several elements were looked at. In some studies, factors were mentioned directly, while in others, facilitators and inhibitors were clear from the context in which they were described. In several papers, the factors might arise from an improvement or deterioration of the outcome measures. Our search included but was not limited to these articles. Therefore, this article focused only on facilitating and inhibiting factors, and not on outcomes. This subject is important for subsequent analysis.

## Results

**Figure 2.**
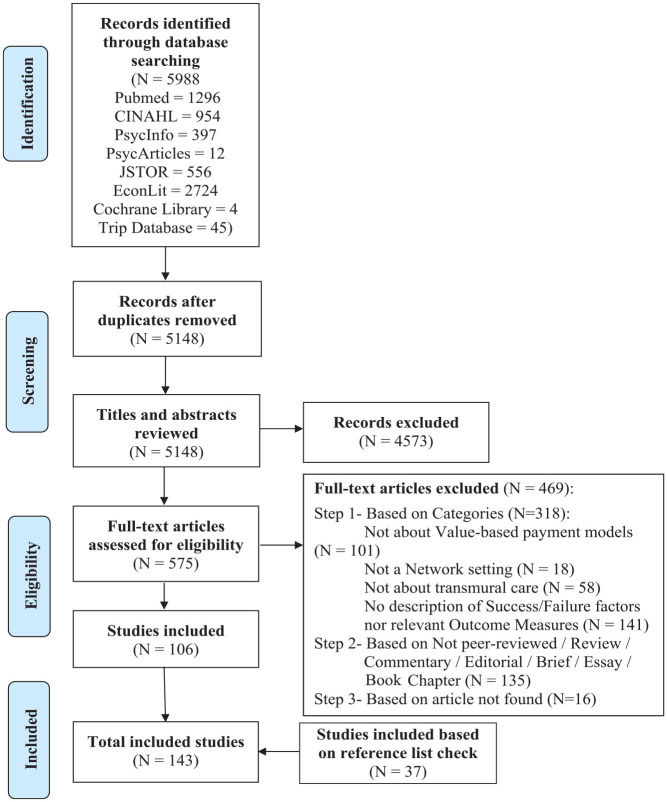
PRISMA Flowchart

The search yielded 5,988 publications, of which 5,148 were included in the first screening phase, after duplicates were eliminated. Based on the inclusion criteria, 575 publications were eligible for the second screening phase. These publications had their full text reviewed, and 106 publications were kept and included in the systematic review. We also checked the reference lists of those publications, as well as the reference lists of the articles that fit the above inclusion criteria but were not original peer-reviewed articles, and 37 publications were included. In total, 143 publications were included, each including one study. More details can be found in Figure 2.

### General Description of the Selected Publications

The main characteristics of the included publications are described in Table 1. Most of the studies (42%) are published between 2017 and 2019. The majority of the studies (76%) have an explanatory aim, only 14 of the 143 studies (10%) are descriptive, and 105 of the 143 included publications (73%) have a quantitative design.

**Table 1.** General Description of the Publications Included in the Analysis.

Classification category and subcategories	*N*	(%)^a^	Reference index in data extraction table (Supplemental Materials)
Year of publication			
2020–2021	24	17	7, 16, 25, 41, 43, 47, 52, 55, 59, 62, 64, 67, 71, 72, 74, 76-78, 89, 91, 96, 100, 102, 138
2017–2019	60	42	2, 3, 8-11, 13-15, 17, 18, 23, 27, 30, 31, 33-35, 37, 39, 42, 44-46, 48-50, 54, 56, 58, 60, 63, 65, 66, 69, 75, 79, 80, 82, 84, 86, 87, 92, 95, 97, 99, 106, 108, 116, 117, 120, 123, 126, 130, 131, 133, 135, 136, 141, 142
2014–2016	35	24	5, 6, 12, 19, 21, 24, 26, 28, 32, 36, 57, 61, 70, 81, 83, 85, 88, 93, 101, 103-105, 107, 111, 113-115, 122, 124, 125, 128, 132, 137, 140, 143
2011–2013	15	10	20, 22, 29, 38, 40, 53, 73, 90, 94, 110, 112, 119, 127, 129, 139
2008–2010	7	5	4, 51, 68, 109, 118, 121, 134
2005–2007	2	1	1, 98
Aim/type of study			
Explanatory	108	76	1-4, 6-14, 16- 24, 27-35, 37-39, 41-48, 50, 53, 56, 58, 61, 62, 64-68, 71, 72, 75-80, 82-101, 105, 106, 109, 110, 112, 114-119, 123, 124, 126-128, 130-,137, 139, 141, 142
Descriptive	14	10	25, 51, 52, 54, 59, 63, 70, 73, 74, 81, 102, 103, 122, 143
Exploratory	21	15	5, 15, 26, 36, 40, 49, 55, 57, 60, 69, 104, 107, 108, 111, 113, 120, 121, 125, 129, 138, 140
Study design			
Quantitative	105	73	1, 2, 3, 6, 8, 9, 11, 13, 14, 16-24, 27-37, 39-46, 50, 51, 53-56, 58, 60-62, 64-68, 72, 75-79, 82-91, 93, 95-97, 99-101, 104, 106, 108-110, 112-117, 119, 122-124, 128-134, 136-143
Qualitative	33	23	4, 5, 7, 10, 12, 15, 25, 26, 47-49, 57, 59, 63, 69, 70, 71, 73, 74, 81, 92, 94, 98, 102, 103, 105, 107, 111, 118, 121, 125-127
Mixed methods	5	3	38, 52, 80, 120, 135
Data collection/data analysis^a^			
Quantitative analysis based on secondary data/patient records	96	67	1-3, 6, 8, 9, 11, 16-24, 27-31, 33-35, 37-39, 41-46, 50, 51, 54-56, 58, 60-62, 64-68, 72, 75, 77-80, 82-87, 89-91, 93, 95-97, 99-101, 104, 106, 108-110, 112, 114-117, 119, 120, 122-124, 128-134, 136, 139, 141-143
Quantitative analysis based on surveys/questionnaires/structured interviews	31	22	11, 13, 14, 32, 34, 36, 38, 46, 52, 53-55, 64, 67, 72, 75, 76, 84, 85, 87, 88, 100, 109, 113, 114, 119, 120, 135, 137, 138, 140
Quantitative analysis based on unstructured/semi-structured/informal interviews	3	2	38, 52, 135
Qualitative analysis based on secondary data/patient records	15	10	10, 25, 38, 63, 69, 70, 73, 74, 80, 103, 105, 107, 118, 120, 126
Qualitative analysis based on surveys/questionnaires/structured interviews	19	13	5, 10, 15, 38, 52, 57, 63, 69, 70, 81, 94, 98, 107, 111, 120, 121, 126, 127, 135
Qualitative analysis based on unstructured/semi-structured/informal interviews	19	13	7, 12, 26, 38, 47-49, 52, 59, 69, 70, 71, 73, 92, 102, 118, 125, 126, 135
Qualitative analysis based on focus group discussions	7	5	4, 5, 57, 73, 74, 81, 111
Qualitative analysis based on observations	6	4	47, 71, 73, 92, 94, 127
Simulation studies	2	1	20, 40
Case studies	14	10	69, 71, 73, 74, 93, 94, 99, 101-103, 105, 106, 126, 127
Quality assessment (quantitative papers)			
Weak	25	17	1, 2, 6, 8, 17, 22, 24, 31, 35, 36, 40, 51, 68, 72, 80, 88, 90, 93, 99, 106, 109, 123, 138, 140, 143
Moderate	40	28	11, 16, 20, 21, 27, 29, 34, 37, 38, 41, 42, 46, 52-55, 64, 66, 67, 76, 78, 79, 84, 86, 87, 91, 101, 108, 112, 113, 115-117, 120, 122, 128, 129, 134, 135, 139
Strong	45	31	3, 9, 13, 14, 18, 19, 23, 28, 30, 32, 33, 39, 43-45, 50, 56, 58, 60-62, 65, 75, 77, 82, 83, 85, 89, 95-97, 100, 104, 110, 114, 119, 124, 130-133, 136, 137, 141, 142
Quality assessment (qualitative papers)			
Weak	7	5	4, 15, 80, 102, 103, 105, 118
Moderate	24	17	5, 7, 10, 12, 25, 26, 38, 47-49, 52, 63, 69, 70, 73, 92, 94, 107, 120, 121, 125-127, 135
Strong	7	5	57, 59, 71, 74, 81, 98, 111

a Percentages refer to the share of all studies reviewed. Percentages may add to more than 100% on each category because studies can report multiple subcategories.

Three main approaches to data collection/analysis are applied in the quantitative studies (e.g., using secondary data/patient records) and five approaches in qualitative studies (e.g., focus group discussions). In addition, there are simulation studies and case studies. Most studies (96 publications, 67%) use a quantitative analysis based on secondary data/patient records. Only two studies (1%) use simulations, and 14 publications (10%) report case studies.

In Table 1, the assessment of study quality is also presented for qualitative and quantitative studies. In total, 45 quantitative and seven qualitative studies have a strong quality (as described in the “Methods” section), which corresponds to 31% and 5% of all publications, respectively. This also includes the assessment of mixed-methods studies, for which quality is assessed using both assessment tools.

### Facilitating Factors of VBP Models

Table 2 presents the facilitating factors of the VBP models found in the literature. The facilitating factors extracted from the included studies are organized according to the framework in Figure 1. Elements are related to the success of the design and development of the VBP model. They refer to a preparatory stage prior to the actual implementation of the models. There are also elements that refer to the success of the implementation of VBP models. After VBP models are implemented, their actual application and evaluation take place. To help with this process, there are elements to take into account. A detailed version of Table 2 can be found in Table 3, in which the facilitating factors identified are grouped into sub-categories, alongside the reference number of the publications that describe those factors. A study may report on multiple factors that belong to different subcategories.

**Table 2.** Facilitating and Inhibiting Factors of Value-Based Payment Models.

	Facilitating factors			Inhibiting factors		
Classification category and subcategories	*N*	(%)^a^	Reference index in data extraction table (Supplemental Materials)	*N*	(%)^a^	Reference index in data extraction table (Supplemental Materials)
Design/development						
Funding	11	8	6, 25, 41, 79, 85, 92, 102, 106, 108, 119, 129	11	8	6, 40, 57, 67, 81, 90, 96, 108, 129, 134, 135
Objectives & strategies	56	39	1, 3, 5, 7, 11, 13, 16, 17, 18, 21, 22, 23, 25, 27, 29, 30, 32, 34, 36, 38, 39, 41, 45, 46, 47, 57, 58, 60, 61, 65, 66, 70, 71, 72, 80, 82, 89, 93, 104, 106, 107, 108, 110, 111, 119, 121, 125, 129, 131, 132, 136, 137, 139, 140, 141, 142	30	21	2, 9, 23, 27, 32-34, 40, 51, 57, 63, 67, 77, 84, 89, 94, 95, 99, 101-103, 109, 116, 121, 132-134, 139, 142, 143
Organizational structure	40	28	2, 4, 7, 8, 12, 13, 16, 19, 24, 27, 28, 37, 39-41, 48, 50, 52, 54, 57, 63, 67, 70, 76, 78, 79, 84, 85, 95-97, 100, 103, 105, 106, 108, 122, 137, 140, 143	10	7	90, 95, 98, 108, 111, 113, 122, 125, 127, 134
Outer context	16	11	7, 8, 17, 32, 35, 65, 68, 69, 82, 86, 96, 99, 112, 115, 117, 119	6	4	52, 73, 90, 104, 119, 126
Psychological antecedents	7	5	14, 70, 71, 87, 105, 125, 138	0	0	NA
Stakeholders	4	3	47, 65, 92, 120	3	2	47, 92, 120
Implementation						
Dedicated time & resources	11	8	3, 10, 13, 23, 27, 35, 61, 63, 124, 128, 130	25	17	3, 7, 8, 10, 13, 17, 23, 27, 35, 53, 63, 73, 79, 84, 91, 102, 103, 112, 117, 124, 127, 133, 136, 140, 141
Leadership & management	6	4	19, 46, 71, 74, 105, 113	0	0	NA
Human resource issues	12	8	1, 12, 19, 27, 44-46, 57, 71, 83, 140, 143	14	10	5, 11, 12, 15, 76, 94, 100, 102, 103, 118, 121, 126, 135, 142
Assessment of implications	0	0	NA	6	4	6, 32, 43, 88, 89, 114
Concerns in early use	4	3	3, 7, 31, 52	9	6	27, 33, 67, 71, 98, 102, 103, 105, 126
Applicability						
Care coordination	36	25	1, 2, 4, 7, 8, 10, 29, 31, 37-40, 52, 54-58, 61, 66, 69, 71, 75, 76, 83, 92, 95, 97, 103, 105, 106, 125, 130, 136, 138, 140	13	9	4, 15, 42, 51, 52, 59, 81, 103, 105, 110, 116, 139, 140
Concerns in established users	0	0	NA	5	3	60, 85, 94, 102, 133
Inequalities	3	2	17, 107, 111	28	20	6, 8, 17, 26, 32, 34, 46, 68, 71, 81, 85, 87, 89, 90, 91, 107, 109, 114, 118, 124-127, 129, 134, 136, 139, 141
Network structures	12	8	4, 24, 39, 54, 56, 63, 72, 73, 103, 108, 120, 130	2	1	83, 106
Standardization & centralization	16	11	4, 7, 38, 40, 48, 49, 53, 57, 60, 76, 88, 95, 105, 107, 121, 123	5	3	12, 57, 59, 67, 77
Target updates	7	5	13, 20, 45, 72, 75, 110, 131	18	13	12, 30, 32, 34, 43, 44, 59, 62, 64, 89, 94, 98, 121, 122, 124, 134, 142, 143

a Percentages add to more than 100% because studies can report multiple facilitating/inhibiting factors.

**Table 3.** Facilitating Factors of Value-Based Payment Models (Expanded).

Classification category and subcategories	Facilitating factors	*N*	(%)^a^	Reference index in data extraction table (Supplemental Materials)
Design/development				
Funding	Sufficient resources to finance a value-based payment model/participate in financial aid programs	11	8	6, 25, 41, 79, 85, 92, 102, 106, 108, 119, 129
Objectives & strategies	Tailored business plan	7	5	25, 45, 57, 66, 119, 121, 129
	Realistic and achievable targets, and clear benchmarks/adequate and frequent incentives to achieve targets (risk-adjusted)	32	22	1, 5, 17, 18, 21-23, 25, 27, 29, 32, 34, 46, 47, 60, 61, 65, 66, 70, 71, 80, 82, 89, 104, 110, 111, 125, 129, 136, 139, 140, 142
	Rotating quality measures, with physicians involved, also rewarding improvement, and linked with savings & new clinical protocols	24	17	1, 3, 11, 13, 18, 22, 23, 25, 27, 38, 39, 58, 66, 72, 104, 107, 121, 129, 131, 132, 137, 139, 141, 142
	Population health management strategies	4	3	3, 7, 41, 106
	Pursue private contracts only after having experience with public contracts	2	1	36, 108
	Distribute payments at department/individual level rather than hospital level	2	1	93, 129
	Model with gains only, which limits risk for healthcare providers	3	2	30, 58, 89
	Model with both gains and losses, which increases the gains for healthcare providers	4	3	16, 18, 41, 46
Organizational structure	Diversity in the composition of professionals involved	18	13	2, 4, 7, 12, 16, 27, 39, 48, 50, 67, 78, 85, 95, 103, 105, 106, 137, 143
	Sufficient infrastructure and advanced health information technology within the network of care	12	8	8, 13, 19, 37, 40, 52, 54, 63, 70, 96, 100, 103
	Involvement of all relevant stakeholders in the design of the model	2	1	57, 140
	Larger organizations, which have fewer initial infrastructure needs and a better alignment across medical practice and hospital settings	6	4	16, 24, 54, 79, 108, 122
	Smaller organizations, which create a more personal setting	8	6	28, 41, 76, 79, 84, 97, 108, 122
Outer context	Model is consistent and/or compatible with location (adjust for regional differences) and current policies	14	10	7, 17, 32, 35, 65, 68, 69, 82, 86, 96, 99, 115, 117, 119
	High-cost/high-performing areas and regions with fewer physician groups	5	3	8, 35, 112, 117, 119
Psychological antecedents	Having a good understanding of the model across involved stakeholders	7	5	14, 70, 71, 87, 105, 125, 138
Stakeholders	Involvement of members of the community from several settings in the management of health facilities	1	1	65
	Involvement of consultancy firms for the management of the program	3	2	47, 92, 120
Implementation				
Dedicated time & resources	Experience with the development, implementation, and evaluation of value-based payment models	10	7	3, 10, 13, 23, 27, 35, 63, 124, 128, 130
	Timely availability of data	2	1	61, 63
Leadership & management	Strong leadership/management	6	4	19, 46, 71, 74, 105, 113
Human resource issues	High motivation, engagement, and trust among involved stakeholders	12	8	1, 12, 19, 27, 44-46, 57, 71, 83, 140, 143
Assessment of implications	Nothing applicable	0	0	NA
Concerns in early use	Adequate access to appropriate care (settings that don’t involve an (overnight) hospital stay for the patient, and including follow-up appointments/postdischarge care programs in the model)	4	3	3, 7, 31, 52
Applicability				
Network structures	Sharing and analyzing historical claims data (learning/open networks)	6	4	4, 72, 73, 108, 120, 130
	Adequate collaboration between clinicians and hospitals (define if organizations in the model are associated with more than one hospital)	6	4	24, 39, 54, 56, 63, 103
Care coordination	Transparency and communication among involved stakeholders	30	21	1, 2, 4, 7, 10, 29, 31, 37-40, 52, 55-57, 61, 66, 71, 76, 83, 92, 95, 103, 105, 106, 125, 130, 136, 138, 140
	Active involvement of medical specialists and/or physicians	9	6	2, 8, 52, 54, 69, 75, 97, 105, 140
	Referral of patients to organizations within the transmural network	2	1	58, 138
Standardization & centralization	Centralized resources, leadership, governance and technology	6	4	7, 48, 49, 95, 105, 123
	Autonomy of healthcare providers to manage their health services	2	1	57, 121
	Adequate trainings for everyone in the team (e.g., for standardized interventions)	9	6	4, 38, 40, 53, 57, 60, 76, 88, 107
Concerns in established users	Nothing applicable	0	0	NA
Target updates	Regular evaluation of the program (make sure it’s having the intended effect)	2	1	13, 131
	Rewarding physicians for excluding no-shows and including patients that started using the program	3	2	20, 45, 72
	Relatively stable patient mix and size	2	1	75, 110
Inequalities	Perceived fairness of the distribution of bonuses	3	2	17, 107, 111

a Percentages refer to the share of all studies reviewed. Percentages add to more than 100% because studies can report multiple facilitating factors.

#### Design and Development

The first category of Table 2 includes publications reporting on the facilitating factors for the design and development of the VBP models, divided into six subcategories. One of the subcategories is funding, mentioned in 11 publications (8%). More specifically, sufficient funding facilitates the development of a VBP model. In total, 39% of all studies report facilitators on objectives and strategies (56 studies), and 28% on organizational structure (40 studies). When considering the objectives and strategies of the VBP model, ensuring realistic and achievable targets, associated with adequate, risk-adjusted, incentives, is the most frequently mentioned facilitating factor (Table 3). Rotating quality measures also rewards improvement and is linked with savings and with physicians involved in their design, which is another very prominent facilitating factor in this subcategory. As for organizational structure, the most frequently mentioned facilitator is diversity in the composition of professionals involved in the design and development of the VBP models. The second most frequently described factor in this subcategory is sufficient infrastructure and advanced health information technology within the NOC, to support the introduction of such a new model. In addition, 16 studies (11%) report outer context facilitators. In this subcategory, a model consistent/compatible with location and current policies is the most frequently described element to facilitate its design. In the “psychological antecedents” subcategory, all involved stakeholders having a prior good understanding of the model helps design a successful model (mentioned in seven studies). Also, four studies report facilitators related to the active involvement of stakeholders. Three of those specifically mention the involvement of consultancy firms in the management of the program as helping with the success of developing a VBP model.

#### Implementation

The second category in Table 2 includes factors to aid in the success of the implementation of VBP models. These elements are further divided into five subcategories. One of those subcategories includes elements related to dedicated time and resources, present in 11 publications (8%). In total, 10 studies state that previous experience in the development, implementation, and evaluation of VBP models aids in the success when implementing the new model (Table 3). Six studies (4%) mention strong and active leadership and management as a supportive element in aligning and motivating internal and external stakeholders. In total, 12 studies (8%) describe factors in the human resource category. More specifically, high motivation, engagement, and trust among involved stakeholders are stated as elements of the success of VBP models. Only 3% of the studies report adequate access to appropriate care as a facilitating factor. This means a model is more successful in a setting that does not involve an overnight hospital stay for the patient and includes follow-up appointments and/or postdischarge care programs. This element is integrated in the “concerns in early use” subcategory.

#### Applicability

The third category in Table 2 relates to the applicability of the VBP models and is subdivided into six other subcategories. One of those subcategories is care coordination, in which success elements are present in 36 studies (25%). The most prominent factor in this subcategory is transparency and communication among involved stakeholders, and active involvement of clinicians, reported in 30 and nine studies, respectively (Table 3). Regarding inequalities, perceived fairness among stakeholders in how bonuses are distributed is mentioned in three studies to facilitate the applicability of VBP models. As for the “network structures” category, learning and open networks sharing and analyzing historical claims are considered in six publications (4%) as a facilitator. The same number of studies points to adequate collaboration between clinicians and hospitals, within a network, as an element of the success of a VBP model. Facilitating factors related to standardization and centralization are mentioned in 16 publications (11%). Within this subcategory, standardized training for everyone in the team is stated by nine studies as helping to make a success of VBP models. Seven publications report facilitators about target updates (5%), in which three elements are included. Making sure the program has the intended effects in regular evaluations and ensuring a relatively stable patient mix and size are two elements mentioned in two studies as facilitators. Rewarding physicians for excluding patients who are included in the program but do not show up, and for including patients that started using the program, is an element of the success of applying the VBP model (three studies).

### Inhibiting Factors of VBP Models

In Table 2, the inhibiting factors related to the studied VBP models are reported. The categories and subcategories are the same as in Table 2 and are the same as in the facilitating factors. Only the factors inside each subcategory differ. In Table 4, a detailed version of Table 2 can be found, and the inhibiting factors found are allocated to each subcategory, alongside the reference number of those publications. There are studies that report multiple factors belonging to different categories and/or subcategories, and other studies do not report barriers.

**Table 4.** Inhibiting Factors of Value-Based Payment Models (Expanded).

Classification category and subcategories	Inhibiting factors	*N*	(%)^a^	Reference index in data extraction table (Supplemental Materials)
Design/development				
Funding	Insufficient resources to finance a value-based payment model	11	8	6, 40, 57, 67, 81, 90, 96, 108, 129, 134, 135
Objectives & strategies	Inadequate outcome measures or targets/insufficient incentives to achieve the targets	21	15	2, 9, 23, 32, 40, 63, 67, 77, 84, 89, 94, 99, 102, 103, 109, 116, 121, 132, 133, 139, 142
	Too much focus on administrative process measures/inadequate or unclear timing of when to move into contract with losses	4	3	34, 51, 103, 143
	Lack of attention given to co-morbidities	5	3	23, 27, 33, 95, 134
	Lack of job description for involved professionals	2	1	57, 121
	Local design, which cannot guarantee a local knowledge about thresholds, performance indicators and reward levels	2	1	94, 101
Organizational structure	Larger organizations, which are more likely to exclude patients from programs and might overlook very local and specialized needs of specific communities	2	1	90, 134
	Smaller organizations, which lack ability to absorb start-up costs and track medication compliance, and get discouraged from making importance changes in processes or inputs of care	6	4	90, 95, 98, 108, 113, 122
	Weak health infrastructure	3	2	111, 125, 127
Outer context	Unsupportive/not considering or adjusting local context (regional differences)	4	3	90, 104, 119, 126
	Unsupportive legislation by the government and other factors outside provider’s control	2	1	52, 73
Psychological antecedents	Nothing applicable	0	0	NA
Stakeholders	Tensions from involvement of consultancy firms	3	2	47, 92, 120
Implementation				
Dedicated time & resources	Excessive amount of time improvements take to manifest	20	14	3, 7, 8, 10, 17, 23, 27, 35, 63, 73, 79, 84, 91, 112, 117, 124, 127, 133, 136, 141
	Low initial quality of care of organizations	1	1	13
	Limited availability of data and/or absence of an integrated information technology system for data registration and sharing	5	3	53, 84, 102, 103, 140
Leadership & management	Nothing applicable	0	0	NA
Human resource issues	Lack of trust among involved stakeholders	5	3	5, 12, 15, 102, 103
	Unfair administration and distribution of gains	1	1	5
	Lack of motivation and engagement among involved stakeholders/lack of uptake of required mechanisms (e.g., telehealth technologies)	9	6	11, 15, 76, 94, 100, 118, 126, 135, 142
	Lack of autonomy of health care providers to manage their health services	1	1	121
Assessment of implications	Difficulty attributing outcomes to specific causes, providers, and/or changes in organizational structure	1	1	6
	Possibility for opportunistic behavior by providers (undertreatment, selection of healthier patients or increasing spending to increase benchmarks)	5	3	32, 43, 88, 89, 114
Concerns in early use	Complexity in the structure of the program/lack of experience to implement required mechanisms	9	6	27, 33, 67, 71, 98, 102, 103, 105, 126
Applicability				
Network structures	Open networks, which might create an overload of patients seeking the most efficient care, neglecting patients that need it the most (with co-morbidities)	1	1	83
	Limited options available to a patient due to tightly controlled and narrow networks of care	1	1	106
Care coordination	Underutilization of primary care physicians & unwillingness of patients to transition from specialists to primary care physicians	3	2	42, 110, 116
	Lack of transparency and communication, and misaligned views among involved stakeholders	10	7	4, 15, 51, 52, 59, 81, 103, 105, 139, 140
Standardization & centralization	Lack of training in population health among involved stakeholders	1	1	12
	Lack of job description for involved professionals/lack of clear and defined standardized approach	4	3	57, 59, 67, 77
Concerns in established users	Lack of compatibility with other value-based payment models in the health care system or current practices in the health care system	2	1	60, 85
	Lack of a patient personalized care plan under the payment models	1	1	133
	Inability to respond to constant payment changes	2	1	94, 102
Target updates	Difficulty of tracking patients who seek care outside the organizations (not under the payment model) & organizations still serving patients that are not beneficiaries under the payment model	4	3	12, 30, 122, 124
	Constant changing benchmarks and patient mix and size/physician turnover	6	4	32, 62, 89, 94, 122, 124
	Inadequate adjustment for differences in patient mix	9	6	34, 43, 44, 59, 64, 98, 134, 142, 143
	Inability of physicians to exclude patients from quality calculations & Freedom to exclude might be abused	1	1	121
Inequalities	Risk that GPs may focus only on the indicators, at the expense of other types of care	2	1	26, 118
	Higher workload of staff due to the program & payment delays/past due	8	6	71, 81, 107, 118, 125-127, 136
	Inequality in the treatment of different groups based on race or disease of a patient	3	2	17, 85, 91
	Rewarding organizations that have performed badly in the past (^a^organizations with better year 1 performance have less improvement between performance years—“ceiling effect”)	3	2	6, 89, 114
	High performing organizations with already low benchmarks don’t have much “fat to trim”)	13	9	8, 32, 34, 46, 68, 87, 90, 109, 124, 129, 134, 139, 141

a Percentages refer to the share of all studies reviewed. Percentages add to more than 100% because studies can report multiple facilitating factors.

#### Design and Development

Regarding the design and development of the VBP models, Table 2 reports on the various factors that inhibit the success of the models. In total, 11 publications (8%) mention the lack of funding as an inhibiting factor. Half of the publications in this category describe inhibiting factors related to objectives and strategies (30 studies). The most frequent factor described as inhibiting the success in the design of VBP models is inadequate outcome measures or targets and/or insufficient incentives to achieve those targets (Table 4). Ten studies report inhibiting factors related to the structure of the organization interested in introducing VBP models (7%). Six of those studies mention that smaller organizations may act as a factor that hinders the development of these models as they lack the ability to absorb start-up costs and track medication compliance. Organizations, in turn, get discouraged from making the necessary changes to successfully implement VBP models in the future. Inhibiting factors related to the outer context are reported in six studies (4%). Four of these six studies mention an unsupportive or lack of consideration in adjusting the design of models given the local context (any regional differences that might exist). As stated in our aim, we applied an organizational perspective, and hence will not go in-depth into this element. However, it is important to point out these inhibiting factors and be aware of their effect. Finally, only three studies (2%) mention tensions between involved consultancy firms (stakeholders) as limiting the success of VBP models.

#### Implementation

Regarding the implementation of VBP models, Table 2 outlines inhibiting factors under this category. In total, 25 studies (17%) report inhibitors related to dedicated time. Overall, 20 of those studies mention that knowing that improvements take a long time to manifest discourages the implementation of VBP models (Table 4). Inhibitors related to human resource issues are mentioned in 14 studies (10%), and nine describe a lack of motivation and engagement among involved stakeholders or lack of uptake of required mechanisms. A lack of trust among the stakeholders involved in the implementation of the models is mentioned in five studies as impeding its success. In the early use of VBP models, there are concerns that hinder its success, reported in nine publications (6%). These concerns are related to the complexity of the structure of the program or lack of experience in implementing required mechanisms. In addition, six studies (4%) mention barriers to the assessment of implications. The most frequently mentioned factor in this subcategory (five studies) is the possibility of opportunistic behavior by providers. This behavior might be translated into selecting healthier patients.

#### Applicability

Regarding the applicability of the VBP models, publications mentioning these inhibiting factors are reported in Table 2. In 13 publications, inhibitors related to care coordination are reported (9%). Within this subcategory, a lack of transparency and communication among involved stakeholders, mentioned in 10 publications, is stated to hinder the potential success of VBP models (Table 4). Factors such as inequalities are reported in 28 studies (20%). In eight of those studies, a higher workload of staff after implementing the program and payment delays are stated to hinder its success. In 18 publications, there are inhibiting factors related to target updates (13%). Half of those studies mention that inadequate adjustments for differences in patient mix act as a barrier to the success of VBP models. In addition, five studies report concerns in established users (3%). Two studies specifically mention a lack of compatibility with other VBP models or with current practices in the health care system. Another two studies mention that the inability to respond to constant payment changes may also impede the successful application of the models. Within “standardization and centralization,” five studies report inhibiting factors (3%). The most prominent factor (four studies) stated that a lack of job description for involved professionals or lack of a clear and defined standardized approach hinders success. Finally, two studies described barriers related to network structures (1%). One publication specifically mentioned that, when applying a VBP model, a tightly controlled and narrow NOC may provide too few options of care for patients.

## Discussion

This systematic literature review presents evidence on the facilitating and inhibiting factors in the design, implementation, and application of VBP models. In our study, there are several factors mentioning stakeholders. This concept refers to anyone with an interest, power, and/or influence in the model, inside (such as managers, nurses, and physicians) or outside (policy makers) the NOC. In addition, articles with weaker quality are not excluded, but triangulated with papers of higher quality, and no singular conclusion is taken from papers with a low quality. The findings show that 40% of the reviewed publications mention objectives and strategies, and organizational structure as facilitators to the design of VBP models. There is a considerable number of studies describing the importance of funding and human resources to the success of VBP models. A similar number of studies describe inhibiting factors in these same subcategories. In addition, an appreciable number of studies outline the presence and lack of prior experience with VBP models as an important factor in the implementation of these models. The inhibiting factor of “excessive amount of time improvements take to manifest” is one of the most prominent factors found in this study. Within the applicability of VBP models, factors regarding care coordination are most prominent, especially in facilitating factors, with 25% of all included studies outlining facilitators that belong to this category.

### Targets and Incentives and Diversity of the Team

A focus on attainable targets and benchmarks, motivating incentives and measures, and evidence-based protocols are the most important elements of these payment models in the literature (Counts et al., 2019; Eijkenaar, 2013; Fisher & Shortell, 2010; Heider & Mang, 2020) and might increase efficiency in health care and quality of health outcomes. In case targets and measures are unrealistic and incentives insufficient, they might act as barriers to the design of VBP models (Counts et al., 2019). National and local legislation needs to be taken into consideration as it can affect the network of organizations and be a major differentiating factor in how to design these models (Chee et al., 2016). In addition, a diverse organizational structure composed of professionals with different backgrounds enriches the organization and contributes to an efficient design of the models, according to the literature (Conrad et al., 2014; Eijkenaar, 2013). This facilitating factor, if lacking, has not seemed to hinder the design and development of VBP models. Further study may be necessary to validate this conclusion, comparing the outcomes of using these models, with and without a diverse team.

### Financial/Human Resources and Prior Experience

The results imply a possible relation between the success of the implementation of VBP models and the availability of financial and human resources, as well as prior experience with VBP models. In the context of collaboration within and between organizations, a well-functioning VBP model is characterized by engagement, trust, and motivation from everyone, together with strong leadership (Conrad et al., 2014; Eijkenaar, 2013; Kuhn & Lehn, 2015). If trust is not built among the stakeholders, or they are unmotivated, the implementation of VBP models can be inhibited (de Vries et al., 2019). In such a complex scenario of NOC and transmural care, having prior experience with these models is an advantage for the implementation of the model. This suggests that, even if VBP models are different given the different contexts of organizations, there are common elements that, if already assimilated, can reduce some of the intricacies of VBP models. Specifically, strong management involving the staff in the change process and implementation of the models may increase their sense of ownership, and hence keep motivation, engagement, and trust at high levels, as suggested by the literature (Haggstrom & Doebbeling, 2011), regardless of the conditions inside and/or outside of the organization.

### Excessive Time Improvements Take to Manifest

Given the complexity of the VBP models, organizations are searching for ways to best implement them, as suggested by the results. With new processes needing to be adapted, tested, and updated, it takes time for improvements to manifest themselves (Heider & Mang, 2020), which might cause friction among the organization’s stakeholders and a subsequent loss of trust in VBP models (Counts et al., 2019). A need arises for all stakeholders to manage expectations and consider that it takes time for such innovative payment models to achieve effects (Milstein & Schreyoegg, 2016). This may be done by the management explaining very clearly the details of VBP models to the organization’s employees, including when and how improvements in efficiency and health outcomes are expected to take place (creating milestones).

### Care Coordination

As indicated by the results, care coordination is a key element in the effective application of VBP models. Transparency and communication among the involved stakeholders are considered vital facilitators, along with involving those stakeholders in the decisions about VBP models. This suggests that, with a high level of transparency and communication, the multidisciplinary team will be more involved and more coordinated, and ultimately perform their tasks more effectively (Kuhn & Lehn, 2015), reducing unnecessary costs, and especially improving quality of care of patients. This collaboration can also go outside the threshold of the organization, namely into the NOC. With hospitals collaborating with each other, for example, by sharing data and communicating about patients, there will be a smoother flow and less waste of both time and money (Kuhn & Lehn, 2015). To achieve a high level of collaboration, results suggest a need for standardization and centralization of resources such as time and knowledge. Training needs to be available for everyone in the team so that everyone knows their tasks and the best way to perform them, keeping autonomy. It may also be that training is needed to explain the idea of VBHC and VBP models as it is not widely implemented (Lally et al., 2019). Without care coordination, communication, and/or transparency among stakeholders, the well-functioning of the VBP model appears to be hindered. This can happen because of misaligned views coming from a lack of standardized training for the team, and a lacking clear approach to take by the stakeholders. However, existing literature does not directly relate these three concepts, and it would be interesting to study how those relate to each other.

### Limitations

Although our review is systematic, and we ensured its quality by following the PRISMA guidelines, there are still some limitations. The cross-check of the screening process was partial (see “Methods” section), and the reference check was only conducted by the primary researcher. This may have created some selection bias. Also, publication bias cannot be entirely excluded as the review only included studies published in academic journals. Some of the included publications did not use random sampling for their data collection, and hence their results may not be generalizable. We provided an extensive overview of facilitating and inhibiting factors, but we did not review information on their importance. Therefore, further research should be conducted, resorting to a consensus among experts, to identify which facilitators and barriers are most important.

## Conclusion

This systematic literature review shows that the effects of facilitating and inhibiting factors of VBP models in the context of NOC and transmural care are recognized and that there are a variety of factors that influence them. Consensus appeared to be a common element among all factors that facilitate the development, implementation, and applicability of VBP models. There needs to be a consensus among stakeholders in the network, such as physicians, nurses, managers, and policy makers, regarding what are the primary objectives of the models, as well as the strategies to achieve those objectives. With different stakeholders’ involvement in the design process, there also needs to be a culture of trust and good coordination in place. Once designed, there appear to be fewer barriers for a VBP model to be successful. Along with the entire network, health care staff needs to be kept motivated and well-informed, as well as actively involved in all phases of the model. It seems to be of vital importance for stakeholders to manage expectations regarding the effectiveness of the models as it takes time to see improvements and the overall added value of VBP models.

## Supplemental Material

sj-docx-1-mcr-10.1177_10775587231160920 – Supplemental material for Facilitating and Inhibiting Factors in the Design, Implementation, and Applicability of Value-Based Payment Models: A Systematic Literature Review Click here for additional data file. Supplemental material, sj-docx-1-mcr-10.1177_10775587231160920 for Facilitating and Inhibiting Factors in the Design, Implementation, and Applicability of Value-Based Payment Models: A Systematic Literature Review by Diogo L. L. Leao, Henricus-Paul Cremers, Dennis van Veghel, Milena Pavlova, Frederique J. Hafkamp and Wim N. J. Groot in Medical Care Research and Review

## References

[bibr1-10775587231160920] Alternative Payment Model (APM) Framework. (2017). Health Care Payment Learning & Action Network. https://hcp-lan.org/apm-framework/

[bibr2-10775587231160920] Bloemen-Vrencken J. H. de Witte L. P. Engels J. P. van den Heuvel W. J. Post M. W. (2005). Transmural care in the rehabilitation sector: Implementation experiences with a transmural care model for people with spinal cord injury. International Journal of Integrated Care, 5, Article e02. 10.5334/ijic.126 PMC1395505 16773154

[bibr3-10775587231160920] Busse R. Klazinga N. Panteli D. Quentin W. (2019). Improving healthcare quality in Europe: Characteristics, effectiveness and implementation of different strategies. 10.1787/b11a6e8f-en 31721544

[bibr4-10775587231160920] Carmone A. E. Kalaris K. Leydon N. Sirivansanti N. Smith J. M. Storey A. Malata A. (2020). Developing a common understanding of networks of care through a scoping study. Health Systems & Reform, 6(2), Article e1810921. 10.1080/23288604.2020.1810921 33021881

[bibr5-10775587231160920] Cattel D. Eijkenaar F. (2020). Value-based provider payment initiatives combining global payments with explicit quality incentives: A systematic review. Medical Care Research and Review, 77(6), 511–537. 31216945 10.1177/1077558719856775 PMC7536531

[bibr6-10775587231160920] Chee T. T. Ryan A. M. Wasfy J. H. Borden W. B. (2016). Current state of value-based purchasing programs. Circulation, 133(22), 2197–2205. 27245648 10.1161/CIRCULATIONAHA.115.010268 PMC5378385

[bibr7-10775587231160920] Conrad D. A. Grembowski D. Hernandez S. E. Lau B. Marcus-Smith M. (2014). Emerging lessons from regional and state innovation in value-based payment reform: Balancing collaboration and disruptive innovation. Milbank Quarterly, 92(3), 568–623. 25199900 10.1111/1468-0009.12078 PMC4221757

[bibr8-10775587231160920] Counts N. Z. Smith J. D. Crowley D. M. (2019). (Expected) value-based payment: From total cost of care to net present value of care. Healthcare, 7(1), 1–3. 10.1016/j.hjdsi.2018.12.005 PMC6684199 30611708

[bibr9-10775587231160920] Cromwell J. Drozd E. M. Smith K. Trisolini M. (2007). Financial gains and risks in pay-for-performance bonus algorithms. Health Care Financing Review, 29(1), 5–14. PMC4195014 18624076

[bibr10-10775587231160920] Damberg C. L. Sorbero M. E. Lovejoy S. L. Martsolf G. R. Raaen L. Mandel D. (2014). Measuring success in health care value-based purchasing programs: Findings from an environmental scan, literature review, and expert panel discussions. RAND Health Quarterly, 4(3), 9. 10.7249/rhq-04-03-09 PMC5161317 28083347

[bibr11-10775587231160920] de Vries E. F. Drewes H. W. Struijs J. N. Heijink R. Baan C. A . (2019). Barriers to payment reform: Experiences from nine Dutch population health management sites. Health Policy, 123(11), 1100–1107. 31578167 10.1016/j.healthpol.2019.09.006

[bibr12-10775587231160920] Eijkenaar F. (2012). Pay for performance in health care: An international overview of initiatives. Medical Care Research and Review, 69(3), 251–276. 22311954 10.1177/1077558711432891

[bibr13-10775587231160920] Eijkenaar F. (2013). Key issues in the design of pay for performance programs. The European Journal of Health Economics, 14(1), 117–131. 21882009 10.1007/s10198-011-0347-6 PMC3535413

[bibr14-10775587231160920] Eijkenaar F. Emmert M. Scheppach M. Schöffski O. (2013). Effects of pay for performance in health care: A systematic review of systematic reviews. Health Policy, 110(2–3), 115–130. 23380190 10.1016/j.healthpol.2013.01.008

[bibr15-10775587231160920] Fisher E. S. Shortell S. M. (2010). Accountable care organizations: Accountable for what, to whom, and how. Journal of the American Medical Association, 304(15), 1715–1716. 20959584 10.1001/jama.2010.1513

[bibr16-10775587231160920] Greenhalgh T. Robert G. Macfarlane F. Bate P. Kyriakidou O. (2004). Diffusion of innovations in service organizations: Systematic review and recommendations. Milbank Quarterly, 82(4), 581–629. 15595944 10.1111/j.0887-378X.2004.00325.x PMC2690184

[bibr17-10775587231160920] Haggstrom D. A. Doebbeling B. N. (2011). Quality measurement and system change of cancer care delivery. Medical Care, 49, S21–S27. 10.1097/MLR.0b013e3181d59529 20940654

[bibr18-10775587231160920] Heider A. K. Mang H. (2020). Effects of monetary incentives in physician groups: A systematic review of reviews. Applied Health Economics and Health Policy, 18(5), 655–667. 32207083 10.1007/s40258-020-00572-x PMC7519000

[bibr19-10775587231160920] Hsieh H. F. Shannon S. E. (2005). Three approaches to qualitative content analysis. Qualitative Health Research, 15(9), 1277–1288. 16204405 10.1177/1049732305276687

[bibr20-10775587231160920] Iglesia E. G. A. Greenhawt M. Shaker M. S. (2020). Achieving the Quadruple Aim to deliver value-based allergy care in an ever-evolving health care system. Annals of Allergy, Asthma & Immunology, 125(2), 126–136. 10.1016/j.anai.2020.04.007 PMC8043132 32289524

[bibr21-10775587231160920] Jha A. K. Joynt K. E. Orav E. J. Epstein A. M. (2012). The long-term effect of premier pay for performance on patient outcomes. New England Journal of Medicine, 366(17), 1606–1615. 22455751 10.1056/NEJMsa1112351

[bibr22-10775587231160920] Kuhn B. Lehn C. (2015). Value-based reimbursement: The banner health network experience. Frontiers of Health Services Management, 32(2), 17–31. 26817267

[bibr23-10775587231160920] Lally K. M. Ducharme C. M. Roach R. L. Towey C. Filinson R. Tuya Fulton A. (2019). Interprofessional training: Geriatrics and palliative care principles for primary care teams in an ACO. Gerontology & Geriatrics Education, 40(1), 121–131. 29630470 10.1080/02701960.2018.1459595

[bibr24-10775587231160920] Lindenauer P. K. Remus D. Roman S. Rothberg M. B. Benjamin E. M. Ma A. Bratzler D. W. (2007). Public reporting and pay for performance in hospital quality improvement. New England Journal of Medicine, 356(5), 486–496. 17259444 10.1056/NEJMsa064964

[bibr25-10775587231160920] Mechanic R. E. (2015). Mandatory Medicare bundled payment—Is it ready for prime time? New England Journal of Medicine, 373(14), 1291–1293. 26308595 10.1056/NEJMp1509155

[bibr26-10775587231160920] Mechanic R. E. Santos P. Landon B. E. Chernew M. E. (2011). Medical group responses to global payment: Early lessons from the “alternative quality contract” in Massachusetts. Health Affairs, 30(9), 1734–1742. 21900665 10.1377/hlthaff.2011.0264

[bibr27-10775587231160920] Mehrotra A. Damberg C. L. Sorbero M. E. Teleki S. S. (2009). Pay for performance in the hospital setting: What is the state of the evidence? American Journal of Medical Quality, 24(1), 19–28. 19073941 10.1177/1062860608326634

[bibr28-10775587231160920] Milstein R. Schreyoegg J. (2016). Pay for performance in the inpatient sector: A review of 34 P4P programs in 14 OECD countries. Health Policy, 120(10), 1125–1140. 27745916 10.1016/j.healthpol.2016.08.009

[bibr29-10775587231160920] Moher D. Liberati A. Tetzlaff J. Altman D. G. (2009). Preferred reporting items for systematic reviews and meta-analyses: The PRISMA statement. PLOS Medicine, 6(7), Article e1000097. 10.1371/journal.pmed.1000097 PMC2707599 19621072

[bibr30-10775587231160920] Porter M. E. (2006). Redefining health care: Creating value-based competition on results. Harvard Business School Press.

[bibr31-10775587231160920] Porter M. E. (2009). A strategy for health care reform—toward a value-based system. New England Journal of Medicine, 361(2), 109–112. 19494209 10.1056/NEJMp0904131

[bibr32-10775587231160920] Porter M. E. Larsson S. Lee T. H. (2016). Standardizing patient outcomes measurement. New England Journal of Medicine, 374(6), 504–506. 26863351 10.1056/NEJMp1511701

[bibr33-10775587231160920] Porter M. E. Lee T. H. (2013). The strategy that will fix health care. https://hbr.org/2013/10/the-strategy-that-will-fix-health-care

[bibr34-10775587231160920] Ramos P. Savage C. Thor J. Atun R. Carlsson K. S. Makdisse M. Neto M. C. Klajner S. Parini P. Mazzocato P. (2021). It takes two to dance the VBHC tango: A multiple case study of the adoption of value-based strategies in Sweden and Brazil. Social Science & Medicine, 282, 114145. 34192620 10.1016/j.socscimed.2021.114145

[bibr35-10775587231160920] Roland M. Campbell S. (2014). Successes and failures of pay for performance in the United Kingdom. New England Journal of Medicine, 370(20), 1944–1949. 24827040 10.1056/NEJMhpr1316051

[bibr36-10775587231160920] Rosenthal M. B. Frank R. G. (2006). What is the empirical basis for paying for quality in health care? Medical Care Research and Review, 63(2), 135–157. 16595409 10.1177/1077558705285291

[bibr37-10775587231160920] Ryan A. M. (2009). Effects of the premier hospital quality incentive demonstration on Medicare patient mortality and cost. Health Services Research, 44(3), 821–842. 19674427 10.1111/j.1475-6773.2009.00956.x PMC2699910

[bibr38-10775587231160920] Ryan A. M. Blustein J. Casalino L. P. (2012). Medicare’s flagship test of pay-for-performance did not spur more rapid quality improvement among low-performing hospitals. Health Affairs, 31(4), 797–805. 22492897 10.1377/hlthaff.2011.0626

