# Supplementary material for: Facilitating and Inhibiting Factors in the Design, Implementation, and Applicability of Value-Based Payment Models: A Systematic Literature Review
Source: Med Care Res Rev. 2023 Mar 23;80(5):467–83. doi: 10.1177/10775587231160920 (PMC10469482; doi:10.1177/10775587231160920)
Supplement: sj-docx-1-mcr-10.1177_10775587231160920 – Supplemental material for Facilitating and Inhibiting Factors in the Design, Implementation, and Applicability of Value-Based Payment Models: A Systematic Literature Review [file sj-docx-1-mcr-10.1177_10775587231160920.docx]

**Additional Materials**

**Additional Material 1** PRISMA 2020 Checklist

| **Section and Topic** | **Item #** | **Checklist item** | **Section Reported** |
| --- | --- | --- | --- |
| **TITLE** | | |  |
| Title | 1 | Identify the report as a systematic review. | Title |
| **ABSTRACT** | | |  |
| Abstract | 2 | See the PRISMA 2020 for Abstracts checklist. | Additional Materials |
| **INTRODUCTION** | | |  |
| Rationale | 3 | Describe the rationale for the review in the context of existing knowledge. | Introduction |
| Objectives | 4 | Provide an explicit statement of the objective(s) or question(s) the review addresses. | Introduction |
| **METHODS** | | |  |
| Eligibility criteria | 5 | Specify the inclusion and exclusion criteria for the review and how studies were grouped for the syntheses. | Methods (Search Strategy & Study Selection Process) |
| Information sources | 6 | Specify all databases, registers, websites, organisations, reference lists and other sources searched or consulted to identify studies. Specify the date when each source was last searched or consulted. | Methods (Search Strategy) |
| Search strategy | 7 | Present the full search strategies for all databases, registers, and websites, including any filters and limits used. | Additional Materials |
| Selection process | 8 | Specify the methods used to decide whether a study met the inclusion criteria of the review, including how many reviewers screened each record and each report retrieved, whether they worked independently, and if applicable, details of automation tools used in the process. | Methods (Study Selection Process) |
| Data collection process | 9 | Specify the methods used to collect data from reports, including how many reviewers collected data from each report, whether they worked independently, any processes for obtaining or confirming data from study investigators, and if applicable, details of automation tools used in the process. | Methods (Data Extraction and Analysis) |
| Data items | 10a | List and define all outcomes for which data were sought. Specify whether all results that were compatible with each outcome domain in each study were sought (e.g. for all measures, time points, analyses), and if not, the methods used to decide which results to collect. | Methods (Data Extraction and Analysis) |
|  | 10b | List and define all other variables for which data were sought (e.g. participant and intervention characteristics, funding sources). Describe any assumptions made about any missing or unclear information. | Not Done |
| Study risk of bias assessment | 11 | Specify the methods used to assess risk of bias in the included studies, including details of the tool(s) used, how many reviewers assessed each study and whether they worked independently, and if applicable, details of automation tools used in the process. | Methods (Study Selection Process & Quality Assessment) |
| Effect measures | 12 | Specify for each outcome the effect measure(s) (e.g. risk ratio, mean difference) used in the synthesis or presentation of results. | Methods (Data Extraction and Analysis) |
| Synthesis methods | 13a | Describe the processes used to decide which studies were eligible for each synthesis (e.g. tabulating the study intervention characteristics and comparing against the planned groups for each synthesis (item #5)). | Not Done |
|  | 13b | Describe any methods required to prepare the data for presentation or synthesis, such as handling of missing summary statistics, or data conversions. | Methods (Data Extraction and Analysis) |
|  | 13c | Describe any methods used to tabulate or visually display results of individual studies and syntheses. | Methods (Data Extraction and Analysis)) |
|  | 13d | Describe any methods used to synthesize results and provide a rationale for the choice(s). If meta-analysis was performed, describe the model(s), method(s) to identify the presence and extent of statistical heterogeneity, and software package(s) used. | Not Done |
|  | 13e | Describe any methods used to explore possible causes of heterogeneity among study results (e.g. subgroup analysis, meta-regression). | Not Done |
|  | 13f | Describe any sensitivity analyses conducted to assess robustness of the synthesized results. | Not Done |
| Reporting bias assessment | 14 | Describe any methods used to assess risk of bias due to missing results in a synthesis (arising from reporting biases). | Not Done |
| Certainty assessment | 15 | Describe any methods used to assess certainty (or confidence) in the body of evidence for an outcome. | Methods (Quality Assessment) |
| **RESULTS** | | |  |
| Study selection | 16a | Describe the results of the search and selection process, from the number of records identified in the search to the number of studies included in the review, ideally using a flow diagram. | Results (initial paragraph) & Figure 2 |
|  | 16b | Cite studies that might appear to meet the inclusion criteria, but which were excluded, and explain why they were excluded. | Results (initial paragraph) |
| Study characteristics | 17 | Cite each included study and present its characteristics. | Results (General description of the selected publications) |
| Risk of bias in studies | 18 | Present assessments of risk of bias for each included study. | Not Done |
| Results of individual studies | 19 | For all outcomes, present, for each study: (a) summary statistics for each group (where appropriate) and (b) an effect estimate and its precision (e.g. confidence/credible interval), ideally using structured tables or plots. | Results |
| Results of syntheses | 20a | For each synthesis, briefly summarise the characteristics and risk of bias among contributing studies. | Not Done |
|  | 20b | Present results of all statistical syntheses conducted. If meta-analysis was done, present for each the summary estimate and its precision (e.g. confidence/credible interval) and measures of statistical heterogeneity. If comparing groups, describe the direction of the effect. | Results |
|  | 20c | Present results of all investigations of possible causes of heterogeneity among study results. | Not Done |
|  | 20d | Present results of all sensitivity analyses conducted to assess the robustness of the synthesized results. | Not Done |
| Reporting biases | 21 | Present assessments of risk of bias due to missing results (arising from reporting biases) for each synthesis assessed. | Not Done |
| Certainty of evidence | 22 | Present assessments of certainty (or confidence) in the body of evidence for each outcome assessed. | Results (General description of the selected publications) |
| **DISCUSSION** | | |  |
| Discussion | 23a | Provide a general interpretation of the results in the context of other evidence. | Discussion |
|  | 23b | Discuss any limitations of the evidence included in the review. | Discussion |
|  | 23c | Discuss any limitations of the review processes used. | Discussion |
|  | 23d | Discuss implications of the results for practice, policy, and future research. | Discussion & Practice Implications |
| **OTHER INFORMATION** | | |  |
| Registration and protocol | 24a | Provide registration information for the review, including register name and registration number, or state that the review was not registered. | Registration and Protocol (in Title Page) |
|  | 24b | Indicate where the review protocol can be accessed, or state that a protocol was not prepared. | Registration and Protocol (in Title Page) |
|  | 24c | Describe and explain any amendments to information provided at registration or in the protocol. | Conducted in PROSPERO |
| Support | 25 | Describe sources of financial or non-financial support for the review, and the role of the funders or sponsors in the review. | Conflicts of Interest and Source of Funding (in Title Page) |
| Competing interests | 26 | Declare any competing interests of review authors. | Conflicts of Interest and Source of Funding (in Title Page) |
| Availability of data, code and other materials | 27 | Report which of the following are publicly available and where they can be found: template data collection forms; data extracted from included studies; data used for all analyses; analytic code; any other materials used in the review. | Additional Materials |

*From:*  Page MJ, McKenzie JE, Bossuyt PM, Boutron I, Hoffmann TC, Mulrow CD, et al. The PRISMA 2020 statement: an updated guideline for reporting systematic reviews. BMJ 2021;372:n71. doi: 10.1136/bmj.n71

**Additional Material 2** Keyword List

| **Component 1** | **Component 2** | **Component 3** |
| --- | --- | --- |
| “Pay for Quality”  Pay-for-Quality  “Payment for Quality”  Payment-for-Quality  “Quality based”  Quality-based  P4Q  “Pay for Performance” (also MeSH)  Pay-for-Performance  “Payment for Performance”  Payment-for-Performance  “Performance based”  Performance-based  P4P  “Payment for Outcome”  Payment-for-Outcome  “Outcome based”  Outcome-based  “Output based”  Output-based  P4O  “Pay for Coordination”  Pay-for-Coordination  “Coordination based”  Coordination-based  P4C  “Value based”  Value-based  Bundled*  “Gain Share”  Gain-Share  “Shared Savings”  Shared-Savings  Risk-sharing  “Risk sharing”  “Episode based”  Episode-based  “Incentive based”  Incentive-based  “Value-based purchasing” (also MeSH)  “Incentive reimbursement” (also MeSH) | Payment*  Reimbursement*  Funding  Compensation*  Spending  Purchasing  Remuneration  Incentive*  “Value-based purchasing” (also MeSH)  Program*  Programme*  Financing  Model | Transmural  Intermural  Network (also MeSH)  “Multiple Organizations”  “Multiple Hospitals”  “Multiple healthcare providers”  “Multiple provider*”  Multiprovider  Interorganizational  “Accountable Care Organization*”  Region* |

**Additional Material 3** PubMed Search

| **Database: Pubmed** | | | |
| --- | --- | --- | --- |
| **Steps** | **Search terms** | **Filters** | **Number of results** |
| **#1** | "pay for quality"[Title/Abstract] OR "pay for quality"[Title/Abstract] OR "payment for quality"[Title/Abstract] OR "payment for quality"[Title/Abstract] OR "quality based"[Title/Abstract] OR "quality based"[Title/Abstract] OR "P4Q"[Title/Abstract] OR "pay for performance"[Title/Abstract] OR "pay for performance"[Title/Abstract] OR "payment for performance"[Title/Abstract] OR "payment for performance"[Title/Abstract] OR "performance based"[Title/Abstract] OR "performance based"[Title/Abstract] OR "P4P"[Title/Abstract] OR "payment for outcome"[Title/Abstract] OR "payment for outcome"[Title/Abstract] OR "outcome based"[Title/Abstract] OR "outcome based"[Title/Abstract] OR "output based"[Title/Abstract] OR "output based"[Title/Abstract] OR "P4O"[Title/Abstract] OR "pay for coordination"[Title/Abstract] OR "pay for coordination"[Title/Abstract] OR "coordination based"[Title/Abstract] OR "coordination based"[Title/Abstract] OR "P4C"[Title/Abstract] OR "value based"[Title/Abstract] OR "value based"[Title/Abstract] OR "bundled*"[Title/Abstract] OR "gain share"[Title/Abstract] OR "gain share"[Title/Abstract] OR "shared savings"[Title/Abstract] OR "shared savings"[Title/Abstract] OR "risk sharing"[Title/Abstract] OR "risk sharing"[Title/Abstract] OR "episode based"[Title/Abstract] OR "episode based"[Title/Abstract] OR "incentive based"[Title/Abstract] OR "incentive based"[Title/Abstract] OR "Value-based purchasing"[Title/Abstract] OR "Incentive reimbursement"[Title/Abstract] OR "pay for performance"[All Fields] OR "Value-based purchasing"[MeSH Terms] | No Filter | 24,034 |
| **#2** | "payment*"[Title/Abstract] OR "reimbursement*"[Title/Abstract] OR "Funding"[Title/Abstract] OR "compensation*"[Title/Abstract] OR "Spending"[Title/Abstract] OR "Purchasing"[Title/Abstract] OR "Remuneration"[Title/Abstract] OR "incentive*"[Title/Abstract] OR "Value-based purchasing"[Title/Abstract] OR "program*"[Title/Abstract] OR "programme*"[Title/Abstract] OR "Financing"[Title/Abstract] OR "Model"[Title/Abstract] OR "Value-based purchasing"[MeSH Terms] | No Filter | 3,296,711 |
| **#3** | "Transmural"[Title/Abstract] OR "Intermural"[Title/Abstract] OR "Network"[Title/Abstract] OR "Multiple Organizations"[Title/Abstract] OR "Multiple Hospitals"[Title/Abstract] OR "Multiple healthcare providers"[Title/Abstract] OR "multiple provider*"[Title/Abstract] OR "Multiprovider"[Title/Abstract] OR "Interorganizational"[Title/Abstract] OR "accountable care organization*"[Title/Abstract] OR "region*"[Title/Abstract] | No Filter | 2,066,861 |
| **#4** | #1 AND #2 AND #3 | Filters - From 2005-2021  English | 1,296 |

**Additional Material 4** CINAHL Search

| **Database: CINAHL** | | | |
| --- | --- | --- | --- |
| **Steps** | **Search terms** | **Filters** | **Number of results** |
| **#1** | TI ( “Pay for Quality” OR Pay-for-Quality OR “Payment for Quality” OR Payment-for-Quality OR “Quality based” OR Quality-based OR P4Q OR “Pay for Performance” OR Pay-for-Performance OR “Payment for Performance” OR Payment-for-Performance OR “Performance based” OR Performance-based OR P4P OR “Payment for Outcome” OR Payment-for-Outcome OR “Outcome based” OR Outcome-based OR “Output based” OR “Output-based” OR P4O OR “Pay for Coordination” OR Pay-for-Coordination OR “Coordination based” OR Coordination-based OR P4C OR “Value based” OR Value-based OR Bundled* OR “Gain Share” OR Gain-Share OR “Shared Savings” OR Shared-Savings OR Risk-Sharing OR “Risk Sharing” OR “Episode based” OR Episode-based OR “Incentive based” OR Incentive-based OR “Value-based purchasing” OR “Incentive reimbursement” ) OR AB ( “Pay for Quality” OR Pay-for-Quality OR “Payment for Quality” OR Payment-for-Quality OR “Quality based” OR Quality-based OR P4Q OR “Pay for Performance” OR Pay-for-Performance OR “Payment for Performance” OR Payment-for-Performance OR “Performance based” OR Performance-based OR P4P OR “Payment for Outcome” OR Payment-for-Outcome OR “Outcome based” OR Outcome-based OR “Output based” OR “Output-based” OR P4O OR “Pay for Coordination” OR Pay-for-Coordination OR “Coordination based” OR Coordination-based OR P4C OR “Value based” OR Value-based OR Bundled* OR “Gain Share” OR Gain-Share OR “Shared Savings” OR Shared-Savings OR Risk-Sharing OR “Risk Sharing” OR “Episode based” OR Episode-based OR “Incentive based” OR Incentive-based OR “Value-based purchasing” OR “Incentive reimbursement” ) OR MW ( “Pay for Performance” OR “Value-based purchasing” OR “Incentive reimbursement” ) | No Filter | 15,533 |
| **#2** | TI ( Payment* OR Reimbursement* OR Funding OR Compensation* OR Spending OR Purchasing OR Remuneration OR Incentive* OR “Value-based purchasing” OR Program* OR Programme* OR Financing OR Model ) OR AB ( Payment* OR Reimbursement* OR Funding OR Compensation* OR Spending OR Purchasing OR Remuneration OR Incentive* OR “Value-based purchasing” OR Program* OR Programme* OR Financing OR Model ) OR MW “Value-based purchasing” | No Filter | 898,363 |
| **#3** | TI ( Transmural OR Intermural OR Network OR “Multiple Organizations” OR “Multiple Hospitals” OR “Multiple healthcare providers” OR “Multiple provider*” OR Multiprovider OR Interorganizational OR “Accountable Care Organization*” OR Region* ) OR AB ( Transmural OR Intermural OR Network OR “Multiple Organizations” OR “Multiple Hospitals” OR “Multiple healthcare providers” OR “Multiple provider*” OR Multiprovider OR Interorganizational OR “Accountable Care Organization*” OR Region* ) OR MW Network | No Filter | 257,087 |
| **#4** | #1 AND #2 AND #3 | Filters - From 2005-2021  English | 954 |

**Additional Material 5** PsycInfo Search

| **Database: PsycInfo** | | | |
| --- | --- | --- | --- |
| **Steps** | **Search terms** | **Filters** | **Number of results** |
| **#1** | TI ( “Pay for Quality” OR Pay-for-Quality OR “Payment for Quality” OR Payment-for-Quality OR “Quality based” OR Quality-based OR P4Q OR “Pay for Performance” OR Pay-for-Performance OR “Payment for Performance” OR Payment-for-Performance OR “Performance based” OR Performance-based OR P4P OR “Payment for Outcome” OR Payment-for-Outcome OR “Outcome based” OR Outcome-based OR “Output based” OR “Output-based” OR P4O OR “Pay for Coordination” OR Pay-for-Coordination OR “Coordination based” OR Coordination-based OR P4C OR “Value based” OR Value-based OR Bundled* OR “Gain Share” OR Gain-Share OR “Shared Savings” OR Shared-Savings OR Risk-Sharing OR “Risk Sharing” OR “Episode based” OR Episode-based OR “Incentive based” OR Incentive-based OR “Value-based purchasing” OR “Incentive reimbursement” ) OR AB ( “Pay for Quality” OR Pay-for-Quality OR “Payment for Quality” OR Payment-for-Quality OR “Quality based” OR Quality-based OR P4Q OR “Pay for Performance” OR Pay-for-Performance OR “Payment for Performance” OR Payment-for-Performance OR “Performance based” OR Performance-based OR P4P OR “Payment for Outcome” OR Payment-for-Outcome OR “Outcome based” OR Outcome-based OR “Output based” OR “Output-based” OR P4O OR “Pay for Coordination” OR Pay-for-Coordination OR “Coordination based” OR Coordination-based OR P4C OR “Value based” OR Value-based OR Bundled* OR “Gain Share” OR Gain-Share OR “Shared Savings” OR Shared-Savings OR Risk-Sharing OR “Risk Sharing” OR “Episode based” OR Episode-based OR “Incentive based” OR Incentive-based OR “Value-based purchasing” OR “Incentive reimbursement” ) OR MA ( “Pay for Performance” OR “Value-based purchasing” OR “Incentive reimbursement” ) | No Filter | 11,200 |
| **#2** | TI ( Payment* OR Reimbursement* OR Funding OR Compensation* OR Spending OR Purchasing OR Remuneration OR Incentive* OR “Value-based purchasing” OR Program* OR Programme* OR Financing OR Model ) OR AB ( Payment* OR Reimbursement* OR Funding OR Compensation* OR Spending OR Purchasing OR Remuneration OR Incentive* OR “Value-based purchasing” OR Program* OR Programme* OR Financing OR Model ) OR MA “Value-based purchasing” | No Filter | 1,104,481 |
| **#3** | TI ( Transmural OR Intermural OR Network OR “Multiple Organizations” OR “Multiple Hospitals” OR “Multiple healthcare providers” OR “Multiple provider*” OR Multiprovider OR Interorganizational OR “Accountable Care Organization*” OR Region* ) OR AB ( Transmural OR Intermural OR Network OR “Multiple Organizations” OR “Multiple Hospitals” OR “Multiple healthcare providers” OR “Multiple provider*” OR Multiprovider OR Interorganizational OR “Accountable Care Organization*” OR Region* ) OR MA Network | No Filter | 300,352 |
| **#4** | #1 AND #2 AND #3 | Filters - From 2005-2021  English | 397 |

**Additional Material 6** PsycArticles Search

| **Database: PsycArticles** | | | |
| --- | --- | --- | --- |
| **Steps** | **Search terms** | **Filters** | **Number of results** |
| **#1** | TI ( “Pay for Quality” OR Pay-for-Quality OR “Payment for Quality” OR Payment-for-Quality OR “Quality based” OR Quality-based OR P4Q OR “Pay for Performance” OR Pay-for-Performance OR “Payment for Performance” OR Payment-for-Performance OR “Performance based” OR Performance-based OR P4P OR “Payment for Outcome” OR Payment-for-Outcome OR “Outcome based” OR Outcome-based OR “Output based” OR “Output-based” OR P4O OR “Pay for Coordination” OR Pay-for-Coordination OR “Coordination based” OR Coordination-based OR P4C OR “Value based” OR Value-based OR Bundled* OR “Gain Share” OR Gain-Share OR “Shared Savings” OR Shared-Savings OR Risk-Sharing OR “Risk Sharing” OR “Episode based” OR Episode-based OR “Incentive based” OR Incentive-based OR “Value-based purchasing” OR “Incentive reimbursement” ) OR AB ( “Pay for Quality” OR Pay-for-Quality OR “Payment for Quality” OR Payment-for-Quality OR “Quality based” OR Quality-based OR P4Q OR “Pay for Performance” OR Pay-for-Performance OR “Payment for Performance” OR Payment-for-Performance OR “Performance based” OR Performance-based OR P4P OR “Payment for Outcome” OR Payment-for-Outcome OR “Outcome based” OR Outcome-based OR “Output based” OR “Output-based” OR P4O OR “Pay for Coordination” OR Pay-for-Coordination OR “Coordination based” OR Coordination-based OR P4C OR “Value based” OR Value-based OR Bundled* OR “Gain Share” OR Gain-Share OR “Shared Savings” OR Shared-Savings OR Risk-Sharing OR “Risk Sharing” OR “Episode based” OR Episode-based OR “Incentive based” OR Incentive-based OR “Value-based purchasing” OR “Incentive reimbursement” ) OR MA ( “Pay for Performance” OR “Value-based purchasing” OR “Incentive reimbursement” ) | No Filter | 414 |
| **#2** | TI ( Payment* OR Reimbursement* OR Funding OR Compensation* OR Spending OR Purchasing OR Remuneration OR Incentive* OR “Value-based purchasing” OR Program* OR Programme* OR Financing OR Model ) OR AB ( Payment* OR Reimbursement* OR Funding OR Compensation* OR Spending OR Purchasing OR Remuneration OR Incentive* OR “Value-based purchasing” OR Program* OR Programme* OR Financing OR Model ) OR MA “Value-based purchasing” | No Filter | 46,930 |
| **#3** | TI ( Transmural OR Intermural OR Network OR “Multiple Organizations” OR “Multiple Hospitals” OR “Multiple healthcare providers” OR “Multiple provider*” OR Multiprovider OR Interorganizational OR “Accountable Care Organization*” OR Region* ) OR AB ( Transmural OR Intermural OR Network OR “Multiple Organizations” OR “Multiple Hospitals” OR “Multiple healthcare providers” OR “Multiple provider*” OR Multiprovider OR Interorganizational OR “Accountable Care Organization*” OR Region* ) OR MA Network | No Filter | 5,636 |
| **#4** | #1 AND #2 AND #3 | Filters - From 2005-2021  English | 12 |

**Additional Material 7** Trip Database Search

| **Database: Trip Database** | | | |
| --- | --- | --- | --- |
| **Steps** | **Search terms** | **Filters** | **Number of results** |
| **#1** | (title:“Pay for Quality” OR Pay-for-Quality OR “Payment for Quality” OR Payment-for-Quality OR “Quality based” OR Quality-based OR P4Q OR “Pay for Performance” OR Pay-for-Performance OR “Payment for Performance” OR Payment-for-Performance OR “Performance based” OR Performance-based OR P4P OR “Payment for Outcome” OR Payment-for-Outcome OR “Outcome based” OR Outcome-based OR “Output based” OR “Output-based” OR P4O OR “Pay for Coordination” OR Pay-for-Coordination OR “Coordination based” OR Coordination-based OR P4C OR “Value based” OR Value-based OR Bundled* OR “Gain Share” OR Gain-Share OR “Shared Savings” OR Shared-Savings OR Risk-Sharing OR “Risk Sharing” OR “Episode based” OR Episode-based OR “Incentive based” OR Incentive-based OR “Value-based purchasing” OR “Incentive reimbursement”) | No Filter | 34,136 |
| **#2** | (title:Payment* OR Reimbursement* OR Funding OR Compensation* OR Spending OR Purchasing OR Remuneration OR Incentive* OR “Value-based purchasing” OR Program* OR Programme* OR Financing OR Model) | No Filter | 61,220 |
| **#3** | (title:Transmural OR Intermural OR Network OR “Multiple Organizations” OR “Multiple Hospitals” OR “Multiple healthcare providers” OR “Multiple provider*” OR Multiprovider OR Interorganizational OR “Accountable Care Organization*” OR Region*) | No Filter | 471,177 |
| **#4** | #1 AND #2 AND #3 | Filters - From 2005-2021  English | 45 |

**Additional Material 8** Cochrane Library Search

| **Database: Cochrane Library** | | | |
| --- | --- | --- | --- |
| **Steps** | **Search terms** | **Filters** | **Number of results** |
| **#1** | (“Pay for Quality” OR Pay-for-Quality OR “Payment for Quality” OR Payment-for-Quality OR “Quality based” OR Quality-based OR P4Q OR “Pay for Performance” OR Pay-for-Performance OR “Payment for Performance” OR Payment-for-Performance OR “Performance based” OR Performance-based OR P4P OR “Payment for Outcome” OR Payment-for-Outcome OR “Outcome based” OR Outcome-based OR “Output based” OR “Output-based” OR P4O OR “Pay for Coordination” OR Pay-for-Coordination OR “Coordination based” OR Coordination-based OR P4C OR “Value based” OR Value-based OR Bundled* OR “Gain Share” OR Gain-Share OR “Shared Savings” OR Shared-Savings OR Risk-Sharing OR “Risk Sharing” OR “Episode based” OR Episode-based OR “Incentive based” OR Incentive-based OR “Value-based purchasing” OR “Incentive reimbursement”):ti,ab,kw | No Filter | 2,043 |
| **#2** | (Payment* OR Reimbursement* OR Funding OR Compensation* OR Spending OR Purchasing OR Remuneration OR Incentive* OR “Value-based purchasing” OR Program* OR Programme* OR Financing OR Model):ti,ab,kw | No Filter | 227,499 |
| **#3** | (Transmural OR Intermural OR Network OR “Multiple Organizations” OR “Multiple Hospitals” OR “Multiple healthcare providers” OR “Multiple provider*” OR Multiprovider OR Interorganizational OR “Accountable Care Organization*” OR Region*):ti,ab,kw | No Filter | 63,313 |
| **#4** | #1 AND #2 AND #3 | Filters - From 2005-2021  English | 78 (4 Cochrane Reviews, 73 Trials and 1 Editorial) |

**Additional Material 9** EconLit Search

| **Database: EconLit** | | | |
| --- | --- | --- | --- |
| **Steps** | **Search terms** | **Filters** | **Number of results** |
| **#1** | TI ( “Pay for Quality” OR Pay-for-Quality OR “Payment for Quality” OR Payment-for-Quality OR “Quality based” OR Quality-based OR P4Q OR “Pay for Performance” OR Pay-for-Performance OR “Payment for Performance” OR Payment-for-Performance OR “Performance based” OR Performance-based OR P4P OR “Payment for Outcome” OR Payment-for-Outcome OR “Outcome based” OR Outcome-based OR “Output based” OR “Output-based” OR P4O OR “Pay for Coordination” OR Pay-for-Coordination OR “Coordination based” OR Coordination-based OR P4C OR “Value based” OR Value-based OR Bundled* OR “Gain Share” OR Gain-Share OR “Shared Savings” OR Shared-Savings OR Risk-Sharing OR “Risk Sharing” OR “Episode based” OR Episode-based OR “Incentive based” OR Incentive-based OR “Value-based purchasing” OR “Incentive reimbursement” ) OR AB ( “Pay for Quality” OR Pay-for-Quality OR “Payment for Quality” OR Payment-for-Quality OR “Quality based” OR Quality-based OR P4Q OR “Pay for Performance” OR Pay-for-Performance OR “Payment for Performance” OR Payment-for-Performance OR “Performance based” OR Performance-based OR P4P OR “Payment for Outcome” OR Payment-for-Outcome OR “Outcome based” OR Outcome-based OR “Output based” OR “Output-based” OR P4O OR “Pay for Coordination” OR Pay-for-Coordination OR “Coordination based” OR Coordination-based OR P4C OR “Value based” OR Value-based OR Bundled* OR “Gain Share” OR Gain-Share OR “Shared Savings” OR Shared-Savings OR Risk-Sharing OR “Risk Sharing” OR “Episode based” OR Episode-based OR “Incentive based” OR Incentive-based OR “Value-based purchasing” OR “Incentive reimbursement” ) | No Filter | 64,343 |
| **#2** | TI ( Payment* OR Reimbursement* OR Funding OR Compensation* OR Spending OR Purchasing OR Remuneration OR Incentive* OR “Value-based purchasing” OR Program* OR Programme* OR Financing OR Model ) OR AB ( Payment* OR Reimbursement* OR Funding OR Compensation* OR Spending OR Purchasing OR Remuneration OR Incentive* OR “Value-based purchasing” OR Program* OR Programme* OR Financing OR Model ) | No Filter | 503,801 |
| **#3** | TI ( Transmural OR Intermural OR Network OR “Multiple Organizations” OR “Multiple Hospitals” OR “Multiple healthcare providers” OR “Multiple provider*” OR Multiprovider OR Interorganizational OR “Accountable Care Organization*” OR Region* ) OR AB ( Transmural OR Intermural OR Network OR “Multiple Organizations” OR “Multiple Hospitals” OR “Multiple healthcare providers” OR “Multiple provider*” OR Multiprovider OR Interorganizational OR “Accountable Care Organization*” OR Region* ) | No Filter | 130,573 |
| **#4** | #1 AND #2 AND #3 | Filters - From 2005-2021  English | 2,724 |

**Additional Material 10** JSTOR Search

| **Database: JSTOR** | | | |
| --- | --- | --- | --- |
| **Steps** | **Search terms** | **Filters** | **Number of results** |
| **#1** | (ti:("Bundled Payment*" OR "Bundled-Payment*" OR "Value-based Purchasing" OR "Value-based Payment" OR Pay-for-Performance OR "Pay for Performance" OR "Pay for Quality" OR "Pay-for-Quality" )) AND la:(eng OR en) | Filters - From 2005-2021  English | 142 |
| **#2** | (ab:("Bundled Payment*" OR "Bundled-Payment*" OR "Value-based Purchasing" OR "Value-based Payment" OR Pay-for-Performance OR "Pay for Performance" OR "Pay for Quality" OR "Pay-for-Quality" )) AND la:(eng OR en) | Filters - From 2005-2021  English | 470 |
| **#3** | #1 + #2 | Filters - From 2005-2021  English | 612  (556 after deduplication) |

**Additional Material 11** Kappa Agreement (Title and Abstract)

|  | **1st Screener** | | | |
| --- | --- | --- | --- | --- |
| **2nd Screener** |  | **Include** | **Exclude** | **Total** |
|  | **Include** | 426 | 8 | 434 |
|  | **Exclude** | 3 | 1513 | 1516 |
|  | **Total** | 429 | 1521 | 1950 |

p0 = 0,994

pe = 0,655

Kappa K = 0,984

**Almost Perfect agreement**

**Additional Material 12** Kappa Agreement (Full-Text)

|  | **1st Screener** | | | |
| --- | --- | --- | --- | --- |
| **2nd Screener** |  | **Include** | **Exclude** | **Total** |
|  | **Include** | 3 | 2 | 5 |
|  | **Exclude** | 0 | 170 | 170 |
|  | **Total** | 3 | 172 | 175 |

p0 = 0,992

pe = 0,948

Kappa K = 0,853

**Almost Perfect agreement**

**Additional Material 13** PRISMA 2020 for Abstracts Checklist

| **Section and Topic** | **Item #** | **Checklist item** | **Reported**  **(Yes/No)** |
| --- | --- | --- | --- |
| **TITLE** | | |  |
| Title | 1 | Identify the report as a systematic review. | Yes |
| **BACKGROUND** | | |  |
| Objectives | 2 | Provide an explicit statement of the main objective(s) or question(s) the review addresses. | Yes |
| **METHODS** | | |  |
| Eligibility criteria | 3 | Specify the inclusion and exclusion criteria for the review. | No |
| Information sources | 4 | Specify the information sources (e.g. databases, registers) used to identify studies and the date when each was last searched. | No |
| Risk of bias | 5 | Specify the methods used to assess risk of bias in the included studies. | Yes |
| Synthesis of results | 6 | Specify the methods used to present and synthesize results. | Yes |
| **RESULTS** | | |  |
| Included studies | 7 | Give the total number of included studies and participants and summarize relevant characteristics of studies. | Yes |
| Synthesis of results | 8 | Present results for main outcomes, preferably indicating the number of included studies and participants for each. If meta-analysis was done, report the summary estimate and confidence/credible interval. If comparing groups, indicate the direction of the effect (i.e. which group is favored). | Yes |
| **DISCUSSION** | | |  |
| Limitations of evidence | 9 | Provide a brief summary of the limitations of the evidence included in the review (e.g. study risk of bias, inconsistency and imprecision). | No |
| Interpretation | 10 | Provide a general interpretation of the results and important implications. | Yes |
| **OTHER** | | |  |
| Competing interests | 11 | Specify the primary source of funding for the review. | No |
| Availability of data, code and other materials | 12 | Provide the register name and registration number. | No |

*From:*  Page MJ, McKenzie JE, Bossuyt PM, Boutron I, Hoffmann TC, Mulrow CD, et al. The PRISMA 2020 statement: an updated guideline for reporting systematic reviews. BMJ 2021;372:n71. doi: 10.1136/bmj.n71

**Data Extraction Table**

**(Bellow)**

| **Nº** | **Reference** | **Aim/**  **Type of Study** | **Study Design** | **Data Collection /**  **Data Analysis** | **Quality Assessment** | **Description of the Value-based Payment Model** | **Country** | **Care Setting** | **Type of Medical Condition** | **Outcome Measures** | **Facilitating factors for the use of Value-based Payment models** | **Barriers for the use of Value-based Payment models** |
| --- | --- | --- | --- | --- | --- | --- | --- | --- | --- | --- | --- | --- |
|  |  |  |  |  |  |  |  |  |  |  |  |  |
| 1 | Mandel, K. E.; Kotagal, U. R. (2007). Pay for Performance Alone Cannot Drive Quality | Explanatory | Quantitative | Quantitative analysis based on single and all-payer data from the PHO asthma registry | Weak | Name of the studied model: Workgroup's name is Providers' Coordination of Care Workgroup's; Provider involvement: Physician-Hospital Organization designed the program; Nature of the incentivized entity: Different practices receive different incentives, based on the achieved targets; Motivation behind model: Patients' benefit; Type of incentive: 3 reward levels (pay for participation, pay for network performance and pay for improvement capability); Nº Quality Indicators: Multiple separate indicators; Performance measure: Absolute measures; Flexibility level: Level of rewards present; Type & Size of payment: Monetary incentives of -2% up until 1% (added depending on levels); Motivation behind model: Improve evidence-based care; Payment Channel: Coupled with usual reimbursement; Certainty of the Targets: Targets set; | USA | Pediatric practices | Asthma | Clinical & Cost outcomes (fee schedule, network asthma population receiving "perfect care"/influenza vaccine); | Reward to recognize practices for committing to the improvement collaborative objectives (devoting time and effort among physicians, nurses, office manager and other staff);  Reward to accelerate practice engagement and promote communication and collaboration among practices; Group-level incentive had a powerful effect in promoting shared learning and the spread of successful interventions across providers (higher engagement of providers); Transparency of comparative (maximizing the impact of the group-level incentive); | - |
| 2 | Shetty, V. A.; Balzer, L. B.; Geissler, K. H.; Chin, D. L. (2019). Association Between Specialist Office Visits and Health Expenditures in Accountable Care Organizations | Explanatory | Quantitative | Quantitative analysis based on Centers for Medicare & Medicaid Services Shared Savings Program Accountable Care Organizations Public-Use Files | Weak | Name of the studied model: MSSP ACO (3+ years); Provider involvement: Provider not involved (CMS regulates); Nature of the incentivized entity: Group (organization); Motivation behind model: Lowering costs; Payment Channel: Receive percentage of savings; Certainty of the Targets: Targets set; Frequency of Incentives: Once a year; | USA | Accountable care organizations | Not specified | Clinical outcomes (emergency department visits, hospital discharges, skilled nursing facility discharges, magnetic resonance imaging volume discharges); | Clinician composition is considered to be an important factor in establishing utilization patterns (balance between PCPs and specialists); Coordination and management of patient care and active specialist involvement in the continuum of patient care; | ACOs integrated with acute care facilities may have less financial incentive to reduce inpatient spending when compared with ACOs established on an outpatient, physician group–based practice if bonuses from shared savings do not compensate for lost fee-for-service revenue |
| 3 | Kaufman, B. G.; O'Brien, E. C.; Stearns, S. C.; Matsouaka, R.; Holmes, G. M.; Weinberger, M.; Song, P. H.; Schwamm, L. H.; Smith, E. E.; Fonarow, G. C.; Xian, Y. (2019). The Medicare Shared Savings Program and Outcomes for Ischemic Stroke Patients: a Retrospective Cohort Study | Explanatory | Quantitative | Quantitative analysis based on hospitalization records from GWTG–Stroke registry, linked to CMS denominator and inpatient claims files, and further merged with CMS MSSP files, the Area Deprivation Index, and the Area Heath Resource File | Strong | Name of the studied model: MSSP ACO (3+ years); Provider involvement: Provider not involved (CMS regulates); Nature of the incentivized entity: Group (organization); Motivation behind model: Reduce readmission rates; Payment Channel: Receive percentage of savings; Certainty of the Targets: Targets set; Frequency of Incentives: Once a year; | USA | Hospitals | Ischemic stroke | Clinical outcomes (discharges to home, length of stay, home-time, recurrent stroke or mortality); | New quality measures are phased in (eg. all-cause unplanned admissions for patients with multiple chronic conditions); ACOs use population health management strategies and systemic changes (requires experience); Use of higher intensity care post-acute care settings (eg.inpatient or skilled nursing rehabilitation settings); | Care that may take years to implement; |
| 4 | Thomas, M. E. (2008). The providers' coordination of care: a model for collaboration across the continuum of care | Explanatory | Qualitative | Qualitative analysis based on focus group discussions | Weak | Name of the studied model: Providers' Coordination of Care (workgroup's name); Motivation behind model: Patients' benefit | USA | Hospitals, nursing homes, and home health agencies | Ulcer | - | Create standardized care practices (clear and consistent communication & standardized interventions); Educate all levels of clinical staff to ensure that every person is knowledgeable about treatment and prevention protocols; Development of a network of local venues of care (case manager can facilitate care transitions and teach/assist through the complexity of the healthcare system); | Individual facility possessiveness, lack of coordination, and a non-standardized "language" between professionals |
| 5 | Chimhutu, V.; Songstad, N. G.; Tjomsland, M.; Mrisho, M.; Moland, K. M. (2016). The inescapable question of fairness in Pay-for-performance bonus distribution: a qualitative study of health workers' experiences in Tanzania | Exploratory | Qualitative | Qualitative analysis based in-depth interviews and focus group discussions | Moderate | Name of the studied model: Pilot P4P; Nature of incentivized entity: Selected groups of health workers are entitled to receive some financial incentives; Motivation behind model: Financial; Type of incentive: Only rewards; Nº Quality Indicators: Multiple separate indicators; Performance measure: Absolute measures; Flexibility level: P4P offers financial incentives to health workers upon reaching a predefined threshold in performance on certain indicators; Payment Channel: Coupled with usual reimbursement | Tanzania | Hospitals and dispensaries | Maternal and pediatric care (conditions not specified) | Organization-related Outcomes/Experience (perceived bonus distribution) | P4P design balancing the need of results, maintaining and nurturing social relations in particular settings; | Experience of unfairness in the way bonuses are distributed and administered at the health facility level; Long term tensions and conflicts at the workplace, feeling of injustice related to P4P (effects on motivation, teamwork and social relations at the workplace); |
| 6 | Paul, David P. (2014). The PGP Demonstrations: Were They Sufficient to Justify Accountable Care Organizations?...Physician Group Practice | Explanatory | Quantitative | Quantitative analysis based on empirical evidence used to characterize the Physician Group Practice Demonstration Project | Weak | Name of the studied model: The Physician Group Practice Demonstration Project (5 years); Nature of the incentivized entity: Group (organization); Provider involvement: Provider not involved (CMS regulates); Motivation behind model: Cost savings & quality of care enhancement; Type of incentive: Only rewards, with providers having the chance of earning bonus payments upon meeting cost savings; Nº Quality Indicators: Multiple separate indicators; Certainty of the Targets: Targets set; Frequency of Incentives: Once a year; Payment Channel: Coupled with usual reimbursement; | USA | Accountable care organizations | Not specified | Cost outcomes (Organizations receiving payments, frequency of those payments, monetary value); | CMS has recently included sharing first-dollar savings for ACOs that meet minimum savings thresholds; | Difficulty of attributing savings achieved to any particular cause or set of causes; Provider-specific costs for each participant were used to encourage encouraged inefficiency (if groups were more inneficient before, then they would have a bigger progression margin); Very high startup costs associated with the establishment of an organization (required a high return on investment); |
| 7 | Harvey, Jillian B.; Vanderbrink, Jocelyn; Mahmud, Yasmin; Kitt‐Lewis, Erin; Wolf, Laura; Shaw, Bethany; Ridgely, M. Susan; Damberg, Cheryl L.; Scanlon, Dennis P.; Kitt-Lewis, Erin (2020). Understanding how health systems facilitate primary care redesign | Explanatory | Qualitative | Qualitative analysis based on semi-structured telephone interviews | Moderate | Name of the studied model: Not specified; Payment Channel: Coupled with usual reimbursement; Certainty of the Targets: Targets set; | USA | Health systems | Not specified | - | Advanced team-based care (combinations of different professions), population management (beginning with primary care and extending out to the larger group of community patients), and care coordination (eg. scheduling specialist appointments and coordinating follow-up) should connect primary care with acute care and specialty care; Higher efficiency in the context of a health system where resources can be centralized (eg. regionalized staff, standardized processes, and centralized leadership, governance and technology); | Substantial length of time it takes to see improvements in outcomes (consistent with literature, stating that it may take between 3 and 10 years); |
| 8 | Parasrampuria, S.; Oakes, A. H.; Wu, S. S.; Parikh, M. A.; Padula, W. V. (2018). VALUE AND PERFORMANCE OF ACCOUNTABLE CARE ORGANIZATIONS: A COST-MINIMIZATION ANALYSIS | Explanatory | Quantitative | Quantitative analysis based on publicly available Centers for Medicare & Medicaid Services files | Weak | Name of the studied model: MSSP ACO (3+ years); Provider involvement: Provider not involved (CMS regulates); Nature of the incentivized entity: Group (organization); Motivation behind model: Financial (cost-minimization analysis); Nº Quality Indicators: Weighted composite quality scores for each ACO and organization-level cost savings; Payment Channel: Receive percentage of savings; Certainty of the Targets: Targets set; Frequency of Incentives: Once a year; | USA | Accountable care organizations | Not specified | Cost outcomes (savings); | Infrastructure for coordinated care; Higher quality level means more coordination of care, which means more savings (through more efficiency); High level of integration of services, the earlier the better (saves the most money); | Systematic dropout for organizations that cannot rapidly improve in quality (difficult to determine whether low performing centers would have the ability to improve their quality); Higher quality organizations don't have as much room to improve; |
| 9 | Resnick, M. J.; Graves, A. J.; Gambrel, R. J.; Thapa, S.; Buntin, M. B.; Penson, D. F. (2018). The association between Medicare accountable care organization enrollment and breast, colorectal, and prostate cancer screening | Explanatory | Quantitative | Quantitative analysis based on Medicare enrollment and claims data & Medicaid Services Shared Savings beneficiary file | Strong | Name of the studied model: MSSP ACO (3+ years); Nature of the incentivized entity: Group (organization); Motivation behind model: Patients' benefit (screenings); Payment Channel: Receive percentage of savings; Certainty of the Targets: Targets set; Frequency of Incentives: Once a year; | USA | Accountable care organizations | Breast, colorectal and prostate cancer | Clinical outcomes (breast, colorectal, and prostate cancer screening); | - | No optimal benchmark screening rates that consider and define appropriate and inappropriate variation in rates of prostate cancer screening (eg. there was a reduction in breast cancer screening, which goes towards reducing overscreening, instead of improving underscreening); |
| 10 | Hearld, L. R.; Carroll, N.; Hall, A. (2019). The adoption and spread of hospital care coordination activities under value-based programs | Explanatory | Qualitative | Qualitative analysis based on the 2013 American Hospital Association Annual Survey of Hospitals, the 2013 AHA Survey of Care Systems, and the Health Resources and Services Administration’s 2013 Area Health Resource File | Moderate | Name of studied model: MSSP & Bundled Payment Program ACO (3+ years); Provider involvement: Provider not involved (CMS regulates); Nature of the incentivized entity: Group (organization); Motivation behind model: Financial (care coordination related to cost savings); Payment Channel: Receive percentage of savings; Certainty of the Targets: Targets set; Frequency of Incentives: Once a year; | USA | Accountable care organizations | Not specified | Organization-related Outcomes/Experience (adoption and spread of care coordination activities); | ACOs are more comprehensive programs that entail more pervasive changes in how providers relate to each other to coordinate care; Predictive analysis tools and prospective management used to identify patients at risk of poor care while they are in the community; | Programs need time to develop; |
| 11 | Duggal, R.; Zhang, Y.; Diana, M. L. (2018). The Association Between Hospital ACO Participation and Readmission Rates | Explanatory | Quantitative | Quantitative analysis based on publicly available on Centers for Medicare & Medicaid Services (CMS) website, additional documents released by CMS, and the American Hospital Association 2013 Survey of Care Systems and Payment | Moderate | Name of the studied model: Pioneer & MSSP ACO (3+ years); Provider involvement: Provider not involved (CMS regulates); Nature of the incentivized entity: Group (organization); Motivation behind model: Patients' benefit (readmission rates); Payment Channel: Receive percentage of savings; Certainty of the Targets: Targets set; Frequency of Incentives: Once a year; | USA | Accountable care organizations | Heart failure, acute myocardial infarction and pneumonia | Clinical outcomes (Heart failure, pneumonia and Acute Myocardial Infarction readmission rate); | Readmission rates were more significantly lower in heart failure (HF), and since it is tested as one of the ACO quality measures for care coordination and patient safety, there could be an increased incentive for ACO hospitals to create a care coordination system especially for HF patients in order to meet the goals set; Pioneer ACOs have higher quality benchmarks than MSSP ACOs, making it difficult for providers to adapt to the Pioneer ACO model (benchmarks need to be more "achievable"); | Providers, even with previous experience in care coordination and care management, may still lack the ability to implement mechanisms required by the ACO model; |
| 12 | Pittman, P.; Forrest, E. (2015). The changing roles of registered nurses in Pioneer Accountable Care Organizations | Explanatory | Qualitative | Qualitative analysis based on semistructured phone interviews | Moderate | Name of the studied model: Pioneer ACO (3+ years); Provider involvement: Provider not involved (CMS regulates); Nature of the incentivized entity: Group (organization); Motivation behind model: Cost savings & quality of care enhancement; Type of incentive: Both rewards and penalties; Payment Channel: Receive percentage of savings; Certainty of the Targets: Targets set; Frequency of Incentives: Once a year; | USA | Accountable care organizations | Not specified | Organization-related Outcomes/Experience (organizational changes reported); | Coordiante care across settings, focus on patient engagement and keep patients away from physicians and hospitals (give registered nurses more responsibilities and power, eg. care planning and assessments and care coordiantors); Transfer some of nurses work to unlicensed assistive personnell, who are paid less (more time for nurses to assume more active roles); | Building trust noted as a major challenge (accross settings or in same setting); Lack of training in population health among registered nurses; Roles of each team member are not clear; Difficulty of tracking patients who seek care outside the ACO; |
| 13 | Diana, Mark L.; Yongkang, Zhang; Yeager, Valerie A.; Stoecker, Charles; Counts, Catherine R. (2019). The impact of accountable care organization participation on hospital patient experience | Explanatory | Quantitative | Quantitative analysis based on HCAHPS survey, the American Hospital Association Annual Survey of Hospitals, and the 2013 AHA Survey of Care Systems and Payment | Strong | Name of the studied model: Pioneer & MSSP ACO (3+ years); Provider involvement: Provider not involved (CMS regulates); Nature of the incentivized entity: Group (organization); Motivation behind model: Cost savings & quality of care enhancement; Type of incentive: Only rewards; Nº Quality Indicators: 33 quality metrics; Payment Channel: Receive percentage of savings; Certainty of the Targets: Targets set; Frequency of Incentives: Once a year; | USA | Hospitals | Not specified | Patient-reported & Organization-related Outcomes/Experience (scores on nursing and doctor communication, patient experience); | Hiring new staff, advanced use of health information technology or/and development of new clinical protocols; Self-evaluation conducted by hospitals leaders to examine their preparedness for ACO participation; Prior care coordination and quality improvement experience position; | Hospitals with low initial quality may face challenges in developing care coordination capabilities when participating in an ACO; |
| 14 | Ouayogodé, M. H.; Mainor, A. J.; Meara, E.; Bynum, J. P. W.; Colla, C. H. (2019). Association Between Care Management and Outcomes Among Patients With Complex Needs in Medicare Accountable Care Organizations | Explanatory | Quantitative | Quantitative analysis based on National Survey of ACOs (NSACO) | Strong | Name of the studied model: MSSP ACO (3+ years); Provider involvement: Provider not involved (CMS regulates); Nature of the incentivized entity: Group (organization); Motivation behind model: Cost savings & quality of care enhancement; Payment Channel: Receive percentage of savings; Certainty of the Targets: Targets set; Frequency of Incentives: Once a year; | USA | Accountable care organizations | Frailty and chronic conditions (conditions not specified) | - | First step to better understanding the promise or limitations of care management and coordination is a better understanding of what health care organizations are implementing to address care needs for patients with complex needs; | - |
| 15 | Schur, Claudia L.; Sutton, Janet P. (2017). Physicians In Medicare ACOs Offer Mixed Views Of Model For Health Care Cost And Quality | Exploratory | Qualitative | Qualitative analysis based on questionnaires to physicians | Weak | Name of the studied model: MSSP & Pioneer ACO (3+ years); Provider involvement: Provider not involved (CMS regulates); Nature of the incentivized entity: Group (organization) Motivation behind model: Patient benefit & Financial interest; Type of Incentive: Both rewards and penalties; Payment Channel: Receive percentage of savings; Certainty of the Targets: Targets set; Frequency of Incentives: Once a year; | USA | Accountable care organizations | Not specified | Organization-related Outcomes/Experience (physician's thoughts toward ACOs); | - | Only a small portion of physicians are affiliated with ACOs in leadership roles (don't see themselves as central to reducing health care costs); Physicians' low awareness of eligibility for shared savings and of whether they or their practice faced financial risk from participation, limited knowledge of which beneficiaries were attributed to the ACO; Low involvement of physicians in the decision to participate in the Medicare ACO program; Many participating physicians’ views are not aligned with ACO goals and that the physicians are divided as to whether or not the ACO model is effective; Physicians participating in Medicare ACOs are only moderately convinced that ACOs are effective; |
| 16 | Nyweide, D. J.; Lee, W.; Colla, C. H. (2020). Accountable Care Organizations' Increase In Nonphysician Practitioners May Signal Shift For Health Care Workforce | Explanatory | Quantitative | Quantitative analysis based on Medicare Shared Savings Program ACO provider-level research Identifiable Files and the public use files | Moderate | Name of the studied model: MSSP ACO (3+ years); Provider involvement: Provider not involved (CMS regulates); Nature of the incentivized entity: Group (organization) Motivation behind model: Patient benefit & Financial interest; Payment Channel: Receive percentage of savings; Certainty of the Targets: Targets set; Frequency of Incentives: Once a year; | USA | Accountable care organizations | Not specified | Organization-related Outcomes/Experience (ACO composition); | Recruiting more nonphysician practitioners (enables ACOs to manage the care of patients and coordinate care for patients between office visits, after hospital discharge, and across care settings); Organize care delivery around a care team in a value-based care model rather than around a single primary care physician; Larger ACOs, as well as models with downside risk (more potential gains); | - |
| 17 | Lin, Yi-Ling; Ortiz, Judith; Boor, Celeste (2018). ACOs' impact on hospitalization rates of rural older adults with diabetes: Early indications | Explanatory | Quantitative | Quantitative analysis based on data from clinics | Weak | Name of the studied model: MSSP ACO (3+ years); Provider involvement: Provider not involved (CMS regulates); Nature of the incentivized entity: Group (organization); Motivation behind model: Patient benefit & Financial interest; Nº Quality Indicators: 34 quality measures; Payment Channel: Receive percentage of savings; Certainty of the Targets: Targets set; Frequency of Incentives: Once a year; | USA | Rural Health Clinics | Diabetes | Clinical outcomes (diabetes-related hospitalization rates); | Even if the new payment incentives offered to ACO participants contribute to improvements in quality of care, it must be determined whether these improvements translate into health disparities reductions; Need to consider that different regions have different levels of income, education and transportation needs; | Since it is early in ACO history, there is lack of evidence of ACO impact on clinical outcomes (implementation of new operational systems, and the communication and coordination between participants), and may require multiple years before measurable impact can be observed; Lower level of trust spotted between African American patients and their physicians as opposed to Whites (may compromise medication adherence and continuity of care); |
| 18 | Borza, T.; Oerline, M. K.; Skolarus, T. A.; Norton, E. C.; Dimick, J. B.; Jacobs, B. L.; Herrel, L. A.; Ellimoottil, C.; Hollingsworth, J. M.; Ryan, A. M.; Miller, D. C.; Shahinian, V. B.; Hollenbeck, B. K. (2019). Association Between Hospital Participation in Medicare Shared Savings Program Accountable Care Organizations and Readmission Following Major Surgery | Explanatory | Quantitative | Quantitative analysis based on a 20% national Medicare sample | Strong | Name of the studied model: MSSP ACO (3+ years); Provider involvement: Provider not involved (CMS regulates); Nature of the incentivized entity: Group (organization); Motivation behind model: Financial (readmission rates lowering costs); Type of Incentive: Both rewards and penalties; Nº Quality Indicators: One quality indicator (readmission rates); Payment Channel: Receive percentage of savings; Certainty of the Targets: Targets set; Frequency of Incentives: Once a year; | USA | Accountable care organizations | Acute myocardial infraction, pneumonia, congestive heart failure, obstructive pulmonary disease, and elective total knee and hip arthroplasty | Clinical outcomes (30-day risk-adjusted readmission rate); | ACOs proactively responded to strong financial incentives implied by both the possibility of Shared Savings bonuses and penalties; ACOs are more likely to use multiple quality improvement tools to identify and track readmissions; | - |
| 19 | Colla, Carrie H.; Lewis, Valerie A.; Kao, Lee-Sien; O'Malley, A. James; Chang, Chiang-Hua; Fisher, Elliott S. (2016). Association Between Medicare Accountable Care Organization Implementation and Spending Among Clinically Vulnerable Beneficiaries | Explanatory | Quantitative | Quantitative analysis based on a random 40% sample of Medicare population drawn from continuously enrolled fee-for-service beneficiaries | Strong | Name of the studied model: Pioneer & MSSP ACO (3+ years); Provider involvement: Provider not involved (CMS regulates); Nature of the incentivized entity: Group (organization); Motivation behind model: Financial (readmission rates lowering costs); Type of incentive: Only rewards; Payment Channel: Receive percentage of savings; Certainty of the Targets: Targets set; Frequency of Incentives: Once a year; | USA | Accountable care organizations | "The clinicaly vulnerable" (conditions not specified) | Clinical & Cost outcomes (total spending, spending in clinically vulnerable, 30-day risk-adjusted readmission rate, hospitalizations); | Factors contributing to success may include ACO participant characteristics such as size, homogeneity, readiness, or structural attributes (eg., whether or not the ACO includes a hospital); market conditions; choice of risk stratification tools; differences in governance models or leadership effectiveness; physician engagement strategies; electronic health records and other health information technology tools; and how providers approached disease management, care transitions, and quality improvement; | - |
| 20 | Lewis, Valerie A.; McClurg, Asha Belle; Smith, Jeremy; Fisher, Elliott S.; Bynum, Julie P. W. (2013). Attributing Patients To Accountable Care Organizations: Performance Year Approach Aligns Stakeholders' Interests | Explanatory | Quantitative | Quantitative analysis based on Medicare fee-for-service claims data to simulate a set of ACOs | Moderate | Name of the studied model: ACO related model (not specified further) (3+ years); Provider involvement: Provider not involved (CMS regulates); Nature of the incentivized entity: Group (organization); Payment Channel: Receive percentage of savings; Certainty of the Targets: Targets set; Frequency of Incentives: Once a year; | USA | Hospital networks | Not specified | - | Removing from the attributed population those patients who do not use the ACO and including patients who have initiated care at the ACO (ensures that physicians are held accountable for the costs and quality of care); | - |
| 21 | McWilliams, J. Michael (2016). Changes in Medicare Shared Savings Program Savings From 2013 to 2014 | Explanatory | Quantitative | Quantitative analysis based on a random 20% sample of Medicare fee-for-service beneficiaries | Moderate | Name of the studied model: MSSP ACO (3+ years); Provider involvement: Provider not involved (CMS regulates); Nature of the incentivized entity: Group (organization); Motivation behind model: Patient benefit & Financial interest; Type of incentive: Only rewards, with ACOs eligible for shared savings bonuses upon spending reductions; Payment Channel: Receive percentage of savings; Certainty of the Targets: Targets set; Frequency of Incentives: Once a year; | USA | Accountable care organizations | Not specified | Cost outcomes (spending); | The growth in MSSP savings suggests continued growth may be possible, particularly if incentives for ACOs to lower spending are strengthened; | - |
| 22 | Song, Z.; Safran, D. G.; Landon, B. E.; Landrum, M. B.; He, Y.; Mechanic, R. E.; Day, M. P.; Chernew, M. E. (2012). The 'Alternative Quality Contract,' based on a global budget, lowered medical spending and improved quality | Explanatory | Quantitative | Quantitative analysis based on Blue Cross Blue Shield of Massachusetts enrollees | Weak | Name of the studied model: Blue Cross Blue Shield Alternative Quality Contract (3+ years); Provider involvement: Provider not involved (CMS regulates); Nature of the incentivized entity: Group (organization); Motivation behind model: Cost savings & quality of care enhancement; Type of Incentive: Similar to Pioneer ACOs, it includes both rewards and penalties; Nº Quality Indicators: Multiple separate indicators; Payment Channel: Receive percentage of savings; Certainty of the Targets: Targets set; Frequency of Incentives: Once a year; | USA | Accountable care organizations | Chronic care management, and adult preventive and pediatric care (conditions not specified) | Clinical & Cost outcomes (savings and quality of care); | Set targets based on actuarial projections to save money over its five-year duration, accounting for anticipated quality bonuses and other payments; Incentives for Medicare beneficiaries and their providers to lower volume without sacrificing quality are key; The long-term success of the model will depend both on how well budgets and bonuses are set and how well groups are able to allocate resources and improve quality within budgets; | - |
| 23 | Gilstrap, L. G.; Huskamp, H. A.; Stevenson, D. G.; Chernew, M. E.; Grabowski, D. C.; McWilliams, J. M. (2018). Changes In End-Of-Life Care In The Medicare Shared Savings Program | Explanatory | Quantitative | Quantitative analysis based on Medicare claims and enrollment files for a random 20% sample of fee-for-service beneficiaries | Strong | Name of the studied model: MSSP ACO (3+ years); Provider involvement: Provider not involved (CMS regulates); Nature of the incentivized entity: Group (organization); Motivation behind model: Patient benefit & Financial interest; Type of incentive: Only rewards, with ACOs eligible for shared savings bonuses upon spending reductions; Payment Channel: Receive percentage of savings; Certainty of the Targets: Targets set;; Frequency of Incentives: Once a year; | USA | Accountable care organizations | "Decedents and high-risk patients" (conditions not specified) | Clinical outcomes (nº transitions/days at home/received intensive care unit (ICU) care in the last 30 days of life, days in ICU care); | Strengthen the ACO's incentives (higher shared-savings rates and financial benchmarks that are not reduced when ACOs lower spending); Need to evaluate the best quality metrics and choose the best ones (the incentive to change performance on any one metric is weak if there are numerous metrics; ACOs need experience and capabilities of the stakeholders for a success; | In the early years of MSSP participation, ACOs may be more inclined to pursue lower-cost, less complex strategies to reduce spending (not include hospice and palliative care providers), due to high costs of hiring or partnering with palliative and end-of-life specialists; Incentives not strong enough; ACOs may need more time, experience, or capabilities before they demonstrate significant progress |
| 24 | Schulz, J.; DeCamp, M.; Berkowitz, S. A. (2015). Medicare Shared Savings Program: public reporting and shared savings distributions | Explanatory | Quantitative | Quantitative analysis based on official ACO websites | Weak | Name of the studied model: MSSP ACO (3+ years); Provider involvement: Provider not involved (CMS regulates); Nature of the incentivized entity: Group (organization); Motivation behind model: Patient benefit & Financial interest; Type of incentive: Only rewards; Payment Channel: Receive percentage of savings; Certainty of the Targets: Targets set; Frequency of Incentives: Once a year; | USA | Accountable care organizations | Not specified | - | We found that ACOs that planned to distribute a majority of their savings to providers were more likely to have generated savings, as were ACOs with larger numbers of participating entities; ACOs that included a hospital planned to allocate a larger percentage of shared savings to their staff; | - |
| 25 | Urwin, J. W.; Caldarella, K. L.; Matloubieh, S. E.; Lee, E.; Mugiishi, M.; Kohatsu, L.; Yoshimoto, J.; Tom, J.; Okamura, S.; Wang, E.; Zhu, J.; Emanuel, E. J.; Volpp, K. G.; Navathe, A. S. (2020). Designing a commercial medical bundle for cancer care: Hawaii Medical Service Association's Cancer Episode Model | Descriptive | Qualitative | Qualitative analysis on of HMSA's Cancer Episode Model | Moderate | Name of the studied model: HMSA's Cancer Episode Model (3+ years); Provider involvement: Provider not involved; Nature of incentivized entity: Individual; Motivation behind model: Financial (quality measures lowering costs); Type of Incentive: Both rewards and penalties in case organizations opt for two-sided risk; Nº Quality Indicators: Multiple separate indicators; Performance measure: Absolute benchmarks; Flexibility level: Fixed targets; Payment Channel: Coupled with usual reimbursement; Certainty of the Targets: Targets set; | USA | Hospitals | Lung, breast, and colorectal cancer | - | A broader scope and inclusion of the total cost of care for specific conditions make practice transformation more tractable and feasible (create accountability); Focus on a small number of types of care (acilitates overcoming treatment norming), a small number of quality metrics, and a higher network pricing (the first performance period allows for 75% of the benchmark to be based on a practice's prior costs, but by period three, up to 100% will be based on network costs); Reward improvement while not penalizing stable achievement of high performers; First-dollar shared savings (bonuses more achievable); | - |
| 26 | Saint-Lary, O.; Leroux, C.; Dubourdieu, C.; Fournier, C.; François-Purssell, I. (2015). Patients' views on pay for performance in France: a qualitative study in primary care | Exploratory | Qualitative | Qualitative analysis based on patient interviews | Moderate | Name of the studied model: Pay for performance (not specified further); Provider involvement: Provider not involved; Nature of the incentivized entity: GPs (individuals) are rewarded; Type of Incentive: Financially rewarding GPs for achieving quality indicators linked to targets; Performance measure: Absolute measures; Payment Channel: Decoupled from usual reimbursement; Certainty of the Targets: Targets set; | France | Family practices | Diabetes and hypertension | Patient-reported Outcomes/Experience (patients' thoughts towards P4P models); | - | Risk that GPs may focus only on the diseases measured by indicators at the expense of other types of care (eg. listening to patients’ most salient needs); Fear from patients that GPs could become more interested in their bonus than in their patients’ care; |
| 27 | Lam, M. B.; Zheng, J.; Orav, E. J.; Jha, A. K. (2019). Early Accountable Care Organization Results in End-of-Life Spending Among Cancer Patients | Explanatory | Quantitative | Quantitative analysis based on national Medicare claims | Moderate | Name of the studied model: ACO model (not further specified) (3+ years); Provider involvement: Provider not involved (CMS regulates); Nature of the incentivized entity: Group (organization); Motivation behind model: Patient benefit & Financial interest; Payment Channel: Receive percentage of savings; Certainty of the Targets: Targets set; Frequency of Incentives: Once a year; | USA | Accountable care organizations | Cancer | Clinical & Cost outcomes (spending in cancer patients, emergency department visits, inpatient hospitalization, intensive care unit admissions, radiation therapy, chemotherapy, and hospice use); | Given the complexity of cancer patients and cancer care, it would be pertinent to have oncologists involved in the development of these quality metrics; Better target end-of- life care to improve clinical and economic effectiveness; Promote/motivate providers to address goals of care and EOL issues early on; | Data may not capture all the costs associated with end-of-life (increasing cost of cancer care is partially driven by innovations in drugs and devices not incorporated into ACO measurements); More time may be needed to show an effect of ACO introduction on spending and hospitalizations for cancer patients; There has been no measurable impact due to the complexity of end-of-life cancer care (use of multiple types of providers and caretakers, making this coordination of care across settings complicated); |
| 28 | McWilliams, J. M.; Hatfield, L. A.; Chernew, M. E.; Landon, B. E.; Schwartz, A. L. (2016). Early Performance of Accountable Care Organizations in Medicare | Explanatory | Quantitative | Quantitative analysis based on Medicare claims and enrollment data for a random 20% sample of fee-for-service beneficiaries | Strong | Name of the studied model: MSSP ACO (3+ years); Provider involvement: Provider not involved (CMS regulates); Nature of the incentivized entity: Group (organization); Motivation behind model: Patient benefit & Financial interest; Type of Incentive: Not just Track 1, with both rewards and penalties; Nº Quality Indicators: Multiple separate indicators; Payment Channel: Receive percentage of savings; Certainty of the Targets: Targets set; Frequency of Incentives: Once a year; | USA | Accountable care organizations | Not specified | Clinical & Cost outcomes (adjusted Medicare spending, performance on "some quality measures"); | Greater savings for independent primary care groups than for groups integrated with hospitals (independent physician groups have stronger incentives to lower inpatient and hospital outpatient spending); | - |
| 29 | Sutton, M.; Nikolova, S.; Boaden, R.; Lester, H.; McDonald, R.; Roland, M.; Sutton, Matt; Nikolova, Silviya; Boaden, Ruth; Lester, Helen; McDonald, Ruth; Roland, Martin (2012). Reduced mortality with hospital pay for performance in England | Explanatory | Quantitative | Quantitative analysis based on patient-level data from national Hospital Episode Statistics from the NHS Information Centre for Health and Social Care | Moderate | Name of the studied model: Advancing Quality program; Nature of incentivized entity: Bonuses allocated internally to clinical teams whose performance had earned the bonus; Provider involvement: Provider not involved; Motivation behind model: Improve quality of care; Type of incentive: Only rewards; Nº Quality Indicators: 28 quality measures; Performance measure: “Tournament” system in which only the top performers received a bonus; Flexibility level: Several stages of rewards; Payment Channel: Receive percentage of revenue; Certainty of the Targets: Targets set (quartiles measured and payments made based on them; Frequency of Incentives: Bonuses paid 6 months later; | England | Hospitals | Acute myocardial infarction, heart failure, and pneumonia | Clinical outcomes (risk adjusted absolute and relative mortality for pneumonia, acute myocardial infraction and heart failure); | The larger size of the bonuses and the greater probability of earning bonuses in the program; Face-to-face communication, pan-regional participation, and the smaller size of the program makes interactions more productive; | - |
| 30 | McWilliams, J. M.; Hatfield, L. A.; Landon, B. E.; Hamed, P.; Chernew, M. E. (2018). Medicare Spending after 3 Years of the Medicare Shared Savings Program | Explanatory | Quantitative | Quantitative analysis based on a random 20% sample of Medicare beneficiaries | Strong | Name of the studied model: MSSP ACO (3+ years); Provider involvement: Provider not involved (CMS regulates); Nature of the incentivized entity: Group (organization); Motivation behind model: Patient benefit & Financial interest; Type of Incentive: Not just Track 1, with both rewards and penalties; Nº Quality Indicators: Multiple separate indicators; Payment Channel: Receive percentage of savings; Certainty of the Targets: Targets set; Frequency of Incentives: Once a year; | USA | Accountable care organizations | Not specified | Cost outcomes (spending level); | Shared-savings contracts that do not impose a downside risk of financial losses for spending above benchmarks may be effective in lowering Medicare spending; | ACOs provide a range of care for patients who are not covered by their ACO contracts, increasing the chances of substantial losses of fee-for-service profits if they are unable to restrict reductions in utilization to those patients; |
| 31 | Schulz, J.; DeCamp, M.; Berkowitz, A. S. A. (2018). Spending Patterns Among Medicare ACOs That Have Reduced Costs | Explanatory | Quantitative | Quantitative analysis based on the Centers for Medicare & Medicaid Services public use file | Weak | Name of the studied model: MSSP ACO (3+ years); Provider involvement: Provider not involved (CMS regulates); Nature of the incentivized entity: Group (organization); Motivation behind model: Patient benefit & Financial interest; Type of Incentive: Not just Track 1, with both rewards and penalties; Payment Channel: Receive percentage of savings; Certainty of the Targets: Targets set; Frequency of Incentives: Once a year; | USA | Accountable care organizations | Not specified | - | Providing care coordination (eg. improvements in communication) and protocol-based clinical services in the emergency setting could help prevent unnecessary hospitalizations and encourage more appropriate outpatient care; | - |
| 32 | Rose, S.; Zaslavsky, A. M.; McWilliams, J. M. (2016). Variation In Accountable Care Organization Spending And Sensitivity To Risk Adjustment: Implications For Benchmarking | Explanatory | Quantitative | Quantitative analysis based on the Consumer Assessment of Healthcare Providers and Systems survey of fee-for-service Medicare beneficiaries and the survey participants’ linked Medicare claims | Strong | Name of the studied model: MSSP ACO (3+ years); Provider involvement: Provider not involved (CMS regulates); Nature of the incentivized entity: Group (organization); Motivation behind model: Patient benefit & Financial interest; Type of Incentive: Both rewards and penalties; Payment Channel: Receive percentage of savings; Certainty of the Targets: Targets set; Frequency of Incentives: Once a year; | USA | Accountable care organizations | Not specified | Cost outcomes (spending level); | Set benchmarks to risk-adjusted average fee-for-service spending in an area, which should be implemented gradually; | ACOs with higher baseline spending leave the program as benchmarks fall below their reach; Benchmarks could inappropriately reward ACOs for serving healthier patients if risk adjustment is inadequate, thereby establishing incentives for ACOs to attract healthier patients rather than become more efficient; ACO’s spending level during its most recent three-year period serves as the basis for resetting (“rebasing”) its benchmark for it next three-year contract period, which diminishes incentives for ACOs to reduce spending because they are penalized with lower benchmarks; |
| 33 | Lam, M. B.; Figueroa, J. F.; Zheng, J.; Orav, E. J.; Jha, A. K. (2018). Spending Among Patients With Cancer in the First 2 Years of Accountable Care Organization Participation | Explanatory | Quantitative | Quantitative analysis based on a 20% sample of Medicare fee-for-service beneficiaries, Medicare Research Identifiable files | Strong | Name of the studied model: ACO model (not further specified) (3+ years); Provider involvement: Provider not involved (CMS regulates); Nature of the incentivized entity: Group (organization); Motivation behind model: Patient benefit & Financial interest; Type of Incentive: Sharing cost savings; Payment Channel: Receive percentage of savings; Certainty of the Targets: Targets set; Frequency of Incentives: Once a year; | USA | Accountable care organizations | Cancer | Clinical & Cost outcomes (spending and utilization in cancer patients); | - | Lack of consideration for the complexity/high risk of the disease, in this case cancer; Difficulty in implementing strategies to reduce utilization across hospitals and practices, in cancer care. It is possible that ACOs have decided to target other "less complicated" kinds of chronic diseases; |
| 34 | Bleser, W. K.; Saunders, R. S.; Muhlestein, D. B.; McClellan, M. (2019). Why Do Accountable Care Organizations Leave The Medicare Shared Savings Program? | Explanatory | Quantitative | Quantitative analysis based publicly available MSSP ACO data, data from the Leavitt Partners ACO Database, CMS’s Medicare Geographic Variation Public Use File, and data from the 2017 Annual ACO Survey about care coordination, health management, and health information technology competencies | Moderate | Name of the studied model: MSSP ACO (3+ years); Provider involvement: Provider not involved (CMS regulates); Nature of the incentivized entity: Group (organization) Motivation behind model: Patient benefit & Financial interest; Type of Incentive: Not just Track 1, with both rewards and penalties; Payment Channel: Receive percentage of savings; Certainty of the Targets: Targets set; Frequency of Incentives: Once a year; | USA | Accountable care organizations | Not specified | Clinical & Cost outcomes (shared-saving bonuses payments, financial performance benchmarks, market-level Medicare cost growth, lower-risk patients, quality scores, postacute care spending, organizational traits); | Heightened financial incentives tied to quality scores, higher minimum quality attainment levels, or higher thresholds for receipt of full bonus payments; | Inadequate timing of when to move MSSP ACOs into downside risk (needs to be studied and the perfect balance should be achieved); Risk-adjustment system might not adequately account for very sick or frail patients (underpays ACOs); High performing ACOs with already low benchmarks/ACOs that made big improvements might be disadvantaged, since they don't have much "fat to trim", and therefore won't gain the shared savings; |
| 35 | Schulz, J.; DeCamp, M.; Berkowitz, S. A. (2017). Regional cost and experience, not size or hospital inclusion, helps predict ACO success | Explanatory | Quantitative | Quantitative analysis based on CMS ACO performance, and CMS-required public reporting information on official ACO websites | Weak | Name of the studied model: MSSP ACO (3+ years); Provider involvement: Provider not involved (CMS regulates); Nature of the incentivized entity: Group (organization); Motivation behind model: Financial; Type of Incentive: Sharing cost savings; Payment Channel: Receive percentage of savings; Certainty of the Targets: Targets set; Frequency of Incentives: Once a year; | USA | Hopitals and academic medical centers | Not specified | - | Need for consideration of regional differences in ACO success; Learning and improving from experience (eg. which methodologies result in cost savings);  ACOs in high cost regions (associated with higher savings) have more room for improvement (may be excess expenditures that could be reduced more easily through ACOs’ care coordination); | Many cost saving initiatives take time to operationalize and then to accrue financial benefits for the organizations (to learn); |
| 36 | Lewis, Valerie A.; Colla, Carrie H.; Schpero, William L.; Shortell, Stephen M.; Fisher, Elliott S. (2014). POLICY. ACO Contracting With Private and Public Payers: A Baseline Comparative Analysis | Exploratory | Quantitative | Quantitative analysis based on the National Survey of Accountable Care Organizations | Weak | Name of the studied model: Private ACO contracts (Blue Cross Blue Shield, Cigna, Aetna, United HealthCare), Medicare contracts (Pioneer and MSSP) & Medicaid contract (not further specified) (3+ years); Provider involvement: Provider not involved (CMS regulates); Nature of the incentivized entity: Group (organization); Motivation behind model: Cost savings & quality of care enhancement; Type of Incentive: Both rewards and penalties; Performance measure: Absolute measures (benchmarks); Flexibility level: Fixed targets; Payment Channel: Coupled with usual reimbursement; Certainty of the Targets: Targets set; Frequency of Incentives: Once a year; | USA | Accountable care organizations | Not specified | - | ACOs with commercial contracts may have more care management and information technology capabilities than ACOs with only Medicare and/or Medicaid ACO, and should be pursued after these public ones (more straightforward and manageable); | - |
| 37 | Kim, H.; Keating, N. L.; Perloff, J. N.; Hodgkin, D.; Liu, X.; Bishop, C. E. (2019). Aggressive Care near the End of Life for Cancer Patients in Medicare Accountable Care Organizations | Explanatory | Quantitative | Quantitative analysis based on Medicare claims, and MSSP ACO Research Identifiable Files | Moderate | Name of the studied model: MSSP ACO (3+ years); Provider involvement: Provider not involved (CMS regulates); Nature of the incentivized entity: Group (organization); Motivation behind model: Financial; Payment Channel: Receive percentage of savings; Certainty of the Targets: Targets set; Frequency of Incentives: Once a year; | USA | Accountable care organizations | Cancer | Clinical outcomes (ICU admissions during last month of life, repeated hospitalizations); | ACOs may provide better discharge and care management through the introduction of electronic health records, and coordinated care may prevent avoidable hospitalizations; PCPs may have key roles in communicating and clarifying information (eg. assisting patients’ treatment decisions); | - |
| 38 | Jones, M.; Hsu, C.; Pearson, D.; Wolford, D.; Labby, D. (2011). An alternative to pay-for-performance: one health plan's approach to quality improvement | Explanatory | Mixed Methods | Quantitative & Qualitative analysis based on data from web-based surveys, site visits, telephone interviews, and document review of submitted reports | Moderate | Name of the studied model: Care Support and System Innovation (CSSI) Program; Provider Involvement: Involved (one of the major facilitators); Motivation behind model: Patient benefit & Financial interest; Certainty of the Targets: Targets set; | USA | Network of community and private medical providers | Not specified | Organization-related Outcomes/Experience (culture, innovations, provider engagement); | Determine appropriate quality measures given the patient-base and the specific needs of the organization; Level of collaboration between CareOregon and its providers made significant contributions to efforts among participants; Trainings and technical assistance helped providers create a ‘‘culture of improvement’’; | - |
| 39 | Benchetrit, L.; Zimmerman, C.; Bao, H.; Dharmarajan, K.; Altaf, F.; Herrin, J.; Lin, Z.; Krumholz, H. M.; Drye, E. E.; Lipska, K. J.; Spatz, E. S. (2019). Admission diagnoses among patients with heart failure: Variation by ACO performance on a measure of risk-standardized acute admission rates | Explanatory | Quantitative | Quantitative analysis based on Medicare claims data from the Chronic Conditions Data Warehouse | Strong | Name of the studied model: MSSP ACO (3+ years); Provider involvement: Provider not involved (CMS regulates); Nature of the incentivized entity: Group (organization); Motivation behind model: Cost savings & quality of care enhancement; Payment Channel: Receive percentage of savings; Certainty of the Targets: Targets set; Frequency of Incentives: Once a year; | USA | Accountable care organizations | Heart failure | - | Engage in structural efforts to improve quality (eg. supporting a collaborative culture with emphasis on learning and problem solving and multidisciplinary care coordination); Develop high-quality, responsive systems of care that are seamlessly coordinated across primary care and specialty services; | - |
| 40 | Eddy, D. M.; Shah, R. (2012). A simulation shows limited savings from meeting quality targets under the Medicare Shared Savings Program | Exploratory | Quantitative | Simulation studies of Medicare patient population | Weak | Name of the studied model: MSSP ACO (3+ years); Provider involvement: Provider not involved (CMS regulates); Nature of the incentivized entity: Group (organization); Motivation behind model: Cost savings & quality of care enhancement; Type of Incentive: Sharing cost savings; Nº Quality Indicators: Separate and composite measures; Payment Channel: Receive percentage of savings; Certainty of the Targets: Targets set; Frequency of Incentives: Once a year; | USA | Accountable care organizations | Type II diabetes | - | To achieve greater savings, accountable care organizations will have improve use of information technology and care coordination (instead of only based on improving quality targets) Health information technology (eg. point-of-care reminders, being completely chartless), care management programs, and education and feedback to providers regarding populations of patients with a given condition | High start-up costs (study mentions $1.7M); Relying on composite measures carries the risk that an ACO could put resources into one or more of its component measures and succeed in reaching those goals, but not succeed on the other components, and ultimately get no credit for its efforts |
| 41 | Kaufman, B. G.; Anderson, D.; Bleser, W. K.; Muhlestein, D. B.; Smith, N.; Clough, J.; McClellan, M. B.; Saunders, R. (2021). Association of ACO Shared Savings Success and Serious Illness Spending | Explanatory | Quantitative | Quantitative analysis based on Medicare Master Beneficiary Summary File | Moderate | Name of the studied model: MSSP ACO (3+ years); Provider involvement: Provider not involved (CMS regulates); Nature of the incentivized entity: Group (organization); Motivation behind model: Cost savings & quality of care enhancement; Type of Incentive: Not just Track 1, with both rewards and penalties; Nº Quality Indicators: Composite score; Payment Channel: Receive percentage of savings; Certainty of the Targets: Targets set; Frequency of Incentives: Once a year; | USA | Accountable care organizations | Advanced liver disease and cirrhosis, lung, colorectal and endometrial cancer, chronic obstructive pulmonary disease, congestive heart failure, alzheimer’s disease and related dementias, diabetes with complications (including ischemic heart disease or peripheral vascular disease), hip fracture, and renal failure | Cost outcomes (shared savings); | The Advance Payment ACO Model (provides participants with monthly upfront payments); Seriously ill patients is an important consideration for ACOs to achieve MSSP shared savings; ACO Track 2 or 3 participation was the strongest driver of shared savings achievement in this descriptive study (downside risk); Smaller, physician-led organizations; | - |
| 42 | Cole, E. S.; Leighton, C.; Zhang, Y. (2018). Distribution of Visits for Chronic Conditions Between Primary Care and Specialist Providers in Medicare Shared Savings Accountable Care Organizations | Explanatory | Quantitative | Quantitative analysis based on Medicare claims and enrollment data, ACO Shared Savings Program Public Use File, and the American Community Survey and Census data | Moderate | Name of the studied model: MSSP ACO (3+ years); Provider involvement: Provider not involved (CMS regulates); Nature of the incentivized entity: Group (organization); Motivation behind model: Cost savings & quality of care enhancement; Payment Channel: Receive percentage of savings; Certainty of the Targets: Targets set; Frequency of Incentives: Once a year; | USA | Accountable care organizations | Asthma, chronic kidney disease, chronic obstructive pulmonary disease, diabetes, depression, hyperlipidemia, hypertension, and rheumatoid arthritis/osteoarthritis | Clinical outcomes (proportion of chronic condition visits delivered by PCPs); | - | Unwillingness of patients to transition from specialists to PCP (transition can be done at different pace, and that needs to be taken into account); Many ACOs may underutilize PCPs (barriers to that shift could include low numbers of PCPs contracted in the ACO) |
| 43 | Eriksson, T.; Tropp, H.; Wiréhn, A. B.; Levin, LÅ (2020). A pain relieving reimbursement program? Effects of a value-based reimbursement program on patient reported outcome measures | Explanatory | Quantitative | Quantitative analysis based on the Stockholm regional patient registry, Statistics Sweden, and Swedish spine register | Strong | Name of the studied model: The Stockholm VBRP; Nature of incentivized entity: Individual; Motivation behind model: Patient benefit & Financial interest; Type of Incentive: Magnitude of the monetary adjustment depends on the discrepancy between the actual and the predicted outcome (providers might have to pay or receive money); Nº Quality Indicators: One quality measure (pain patients feel 1 year after surgery); Performance measure: Level of pain patients feel vs predicted outcome; Flexibility level: Fixed targets; Type & Size of payment: Monetary positive adjustment can go between 1 and 6% and negative between -1 and -18%; Payment Channel: Coupled with usual reimbursement; Certainty of the Targets: Targets set (decided when predicting the outcome); Frequency of Incentives: Once a year; | Sweden | Hospitals | Elective spine surgery | Patient-reported & Organization-related Outcomes/Experience (inequalities and PROMs); | - | The financial incentive of the P4P within the STHLM-VBRP was primarily focused on avoiding negative outcomes rather than incentivizing positive outcomes. Thus, the financial incentives associated with the P4P within the STHLM-VBRP was more of a whip than a carrot for the healthcare providers; Failing to adjust the reimbursement for variation in risk factors among patient may cause providers to attempt shifting their case-mix of patients toward patients with higher probability of positive outcomes (cherry- picking); |
| 44 | Modi, P. K.; Kaufman, S. R.; Borza, T.; Yan, P.; Miller, D. C.; Skolarus, T. A.; Hollingsworth, J. M.; Norton, E. C.; Shahinian, V. B.; Hollenbeck, B. K. (2018). Variation in prostate cancer treatment and spending among Medicare shared savings program accountable care organizations | Explanatory | Quantitative | Quantitative analysis based on Medicare claims and enrollment data, ACO Shared Savings Program Public Use File, and the American Community Survey and Census data | Strong | Name of the studied model: MSSP ACO (3+ years); Provider involvement: Provider not involved (CMS regulates); Nature of the incentivized entity: Group (organization); Motivation behind model: Cost savings & quality of care enhancement; Payment Channel: Receive percentage of savings; Certainty of the Targets: Targets set; Frequency of Incentives: Once a year; | USA | Accountable care organizations | Prostate cancer | - | Organizations that better engage specialists may be able to improve the value of specialty care; | Urologists may be less likely to treat men unlikely to benefit; |
| 45 | Barnett, M. L.; McWilliams, J. M. (2018). Changes in specialty care use and leakage in Medicare accountable care organizations | Explanatory | Quantitative | Quantitative analysis based on Medicare claims for a 20% sample of beneficiaries | Strong | Name of the studied model: MSSP ACO (3 years); Provider involvement: Provider not involved (CMS regulates); Nature of the incentivized entity: Group (organization); Motivation behind model: Cost savings & quality of care enhancement; Nº Quality Indicators: Multiple separate indicators; Payment Channel: Receive percentage of savings; Certainty of the Targets: Targets set; Frequency of Incentives: Once a year; | USA | Accountable care organizations | Not specified | Clinical outcomes (leakage of specialist visits, contract penetration, new specialist visits); | Redesign the delivery system to support new payment models; Higher co-pays for non-ACO providers; Fostering patient engagement; | - |
| 46 | Ouayogodé, M. H.; Colla, C. H.; Lewis, V. A. (2017). Determinants of success in Shared Savings Programs: An analysis of ACO and market characteristics | Explanatory | Quantitative | Quantitative analysis based on the National Survey of ACOs, Medicare fee-for-service enrollees’ claims data, and data publicly available from CMS | Moderate | Name of the studied model: Pioneer & MSSP ACO (3+ years); Provider involvement: Provider not involved (CMS regulates); Nature of the incentivized entity: Group (organization); Motivation behind model: Cost savings & quality of care enhancement; Type of incentive: Both rewards and penalties; Performance measure: Absolute measures; Payment Channel: Receive percentage of savings; Certainty of the Targets: Targets set; Frequency of Incentives: Once a year; | USA | Accountable care organizations | Not specified | - | Organizations with large financial benchmarks at baseline have greater opportunities to achieve saving; Risk-bearing contracts (with more risks come more rewards); Physician engagement in leadership and through financial incentives may help organizations improve financial performance; | Efficient organizations, who have reduced growth in health care spending prior to ACO implementation, may find it difficult to further reduce spending; |
| 47 | Murray, Genevra F.; D'Aunno, Thomas; Lewis, Valerie A. (2021). Critical issues in alliances between management partners and accountable care organizations...AcademyHealth 2018 Annual Research Meeting in Seattle, Washington | Explanatory | Qualitative | Qualitative analysis based on observations for site visits and semi-structured interviews | Moderate | Name of the studied model: ACO program (not further specified) (3+ years); Provider involvement: Provider not involved (CMS regulates); Nature of the incentivized entity: Group (organization); Motivation behind model: Cost savings & quality of care enhancement; Type of Incentive: In 2/3 of partnerships, the management partner is sharing in financial risk or reward ; Payment Channel: Coupled with usual reimbursement; Certainty of the Targets: Targets set; Frequency of Incentives: Once a year; | USA | Accountable care organizations | Not specified | - | Establish clear expectations and build learning objectives into contracts; If seeking a partner, provider organizations should first determine if they are seeking a long-term differentiated alliance or a shorter-term partnership to achieve learning; | Tensions frequently arose issues of who received and controlled money, over the power that the ACO and the management partner hold, and how the ACO perceives the delivery of the management partner's promises; |
| 48 | Lewis, V. A.; Schoenherr, K.; Fraze, T.; Cunningham, A. (2019). Clinical coordination in accountable care organizations: A qualitative study | Explanatory | Qualitative | Qualitative analysis based on semistructured interviews | Moderate | Name of the studied model: Pioneer & MSSP ACO (3+ years); Provider involvement: Provider not involved (CMS regulates); Nature of the incentivized entity: Group (organization); Motivation behind model: Cost savings & quality of care enhancement; Payment Channel: Receive percentage of savings; Certainty of the Targets: Targets set; Frequency of Incentives: Once a year; | USA | Accountable care organizations | Not specified | - | ACOs may be successful at achieving cost and quality performance through implementation of routines of many sorts, such as standardized care pathways or care transition protocols (could create value through boundary spanning, as ACOs may be able to create improved mechanisms to integrate work across primary care) | - |
| 49 | Lewis, V. A.; Tierney, K. I.; Fraze, T.; Murray, G. F. (2019). Care Transformation Strategies and Approaches of Accountable Care Organizations | Exploratory | Qualitative | Qualitative analysis based on interviews with ACO leaders | Moderate | Name of the studied model: MSSP ACO & Other programs not specified further (3+ years); Nature of the incentivized entity: Group (organization); Payment Channel: Coupled with usual reimbursement; Certainty of the Targets: Targets set; Frequency of Incentives: Once a year; | USA | Accountable care organizations | Not specified | - | Hybrid model of pairing practice-based change with overlay programs may be the most successful model if ACOs are able to pull the most effective pieces of centralized and overlay care to wrap around physician practices with efficient and effective care teams; | - |
| 50 | Sukul, D.; Ryan, A. M.; Yan, P.; Markovitz, A.; Nallamothu, B. K.; Lewis, V. A.; Hollingsworth, J. M. (2019). Cardiologist Participation in Accountable Care Organizations and Changes in Spending and Quality for Medicare Patients With Cardiovascular Disease | Explanatory | Quantitative | Quantitative analysis based on a 20% random sample of Medicare beneficiaries | Strong | Name of the studied model: MSSP ACO (3+ years); Provider involvement: Provider not involved (CMS regulates); Nature of the incentivized entity: Group (organization); Motivation behind model: Cost savings & quality of care enhancement; Nº Quality Indicators: Multiple separate indicators; Payment Channel: Coupled with usual reimbursement; Certainty of the Targets: Targets set; Frequency of Incentives: Once a year; | USA | Accountable care organizations | Cardiovascular disease (conditions not specified) | Clinical & Cost outcomes (spending level, heart failure admission rates, all-cause readmissions, emergency department visits); | Cardiologist participation associated with reduced spending; | - |
| 51 | Glickman, Seth W.; Boulding, William; Roos, Jason M. T.; Staelin, Richard; Peterson, Eric D.; Schulman, Kevin A. (2009). Alternative pay-for-Performance Scoring Methods: Implications for Quality Improvement and Patient Outcomes | Descriptive | Quantitative | Quantitative analysis based on Hospital Compare and Joint Commission Core Measures databases | Weak | Name of the studied model: Pay for performance (not specified further); Provider involvement: Provider not involved; Motivation behind model: Improve quality of care; Nº Quality Indicators: Base pay-for-performance payments on a single summary measure compiled from a number of performance indicators; Payment Channel: Decoupled from usual reimbursement; | USA | Hospitals | Acute myocardial Infarction and heart failure | - | - | P4P schemes that incentivize hospitals to focus too much on administrative process measures may be associated with decreased adherence to clinical processes; Financial constrains in the hospital, and constant pressure and stress to optimize services; |
| 52 | Kennedy, G.; Lewis, V. A.; Kundu, S.; Mousqués, J.; Colla, C. H. (2020). Accountable Care Organizations and Post-Acute Care: A Focus on Preferred SNF Networks | Descriptive | Mixed Methods | Quantitative and Qualitative analysis based on the National Survey of ACOs and semi-structured interviews with ACOs | Moderate | Name of the studied model: MSSP ACO (3+ years); Provider involvement: Provider not involved (CMS regulates); Nature of the incentivized entity: Group (organization); Motivation behind model: Cost savings & quality of care enhancement; Payment Channel: Coupled with usual reimbursement; Certainty of the Targets: Targets set; Frequency of Incentives: Once a year; | USA | Accountable care organizations | Not specified | - | Integrate post-acute care services to create a seamless continuum of care; Support emerging technologies for sharing patient information, and jointly established patient care protocols designed to connect patient health and social needs; High-performing ACOs are establishing preferred SNF networks to an effort to improve coordination of care, reduce hospital readmissions, and shorten lengths of stay; | Misaligned incentives, unclear regulations, a lack of integrated health records, and a lack of actionable post-acute care data to drive performance improvement; |
| 53 | Saleh, Shadi S.; Alameddine, Mohamad S.; Natafgi, Nabil M. (2013). ACCEPTABILITY OF QUALITY REPORTING AND PAY FOR PERFORMANCE AMONG PRIMARY HEALTH CENTERS IN LEBANON | Explanatory | Quantitative | Quantitative analysis based on a survey of managers | Moderate | Name of the studied model: Pay for performance (not specified further); Motivation behind model: Efficiency &Improving health outcomes; Nº Quality Indicators: Multiple separate indicators; Performance measure: Absolute measures; Payment Channel: Decoupled from usual reimbursement; | Lebanon | Primary health center network | Not specified | Organization-related Outcomes/Experience (managers willingness to report clinical performance, opinions towards peer-performance comparison and pay-for-performance schemes); | The great willingness of PHCC managers to employ quality-enhancing initiatives flags a policy priority for PHC stakeholders to strengthen PHCC infrastructure and to enable reporting in an easy, standardized, and systematic way; | Absence of an information system and guidelines, as well as standardized data reporting forms (presence of an information system is indicative of facilities’ availability of financial resources and leadership support of quality and innovation); |
| 54 | Bazzoli, G. J.; Harless, D. W.; Chukmaitov, A. S. (2019). A taxonomy of hospitals participating in Medicare accountable care organizations | Descriptive | Quantitative | Quantitative analysis based on Centers for Medicare and Medicaid Services, American Hospital Association, and Health Information and Management Systems Society | Moderate | Name of the studied model: Pioneer & MSSP ACO (3+ years); Provider involvement: Provider not involved (CMS regulates); Nature of the incentivized entity: Group (organization); Motivation behind model: Cost savings & quality of care enhancement; Payment Channel: Coupled with usual reimbursement; Certainty of the Targets: Targets set; Frequency of Incentives: Once a year; | USA | Accountable care organizations | Not specified | - | Health information technology and physician linkages appear to be particularly important features in ACO hospitals; A strong health IT infrastructure is needed to be present to facilitate care coordination and communication; ACOs with more than one hospital participant chose hospitals that have complementary capabilities (more supportive service and/or geographic coverage); Vertical alignment between hospitals and their affiliated physicians reduces transaction costs;  ACOs with hospital involvement had substantially more primary care and specialist physician participation than ACOs lacking hospitals (better alignment across); | - |
| 55 | Gu, J.; Huckfeldt, P.; Sood, N. (2021). The Effects of Accountable Care Organizations Forming Preferred Skilled Nursing Facility Networks on Market Share, Patient Composition, and Outcomes | Exploratory | Quantitative | Quantitative analysis based on the American Hospital Association hospital survey, Medicare Provider Analysis and Review files, and Master Beneficiary Summary files | Moderate | Name of the studied model: Not specified; Provider involvement: Provider not involved (CMS regulates); Nature of the incentivized entity: Group (organization); Payment Channel: Coupled with usual reimbursement; Certainty of the Targets: Targets set; | USA | Accountable care organizations | Not specified | Clinical outcomes (market share, outcomes, and nº of Elixhauser comorbidities); | ACO hospitals send more complex patients to preferred SNFs after network formation (better targeted care coordination); | - |
| 56 | McWilliams, J. M.; Gilstrap, L. G.; Stevenson, D. G.; Chernew, M. E.; Huskamp, H. A.; Grabowski, D. C. (2017). Changes in Postacute Care in the Medicare Shared Savings Program | Explanatory | Quantitative | Quantitative analysis based on a 20% sample of beneficiaries | Strong | Name of the studied model: MSSP ACO (3+ years); Provider involvement: Provider not involved (CMS regulates); Nature of the incentivized entity: Group (organization); Motivation behind model: Cost savings & quality of care enhancement; Nº Quality Indicators: Multiple separate indicators; Payment Channel: Coupled with usual reimbursement; Certainty of the Targets: Targets set; Frequency of Incentives: Once a year; | USA | Accountable care organizations | Acute and post-acute care (conditions not specified) | Clinical & Cost outcomes (post-acute spending, inpatient utilization, discharges to facilities rather than home, SNF use/spending, length of stay, 30-day readmissions, mortality); | Clinicians working within hospitals and SNFs to influence care for ACO patients than with hospital-wide initiatives by ACOs or use of preferred SNFs; Employing or partnering with clinicians to follow patients in SNFs; | - |
| 57 | Manongi, R.; Mushi, D.; Kessy, J.; Salome, S.; Njau, B. (2014). Does training on performance based financing make a difference in performance and quality of health care delivery? Health care provider's perspective in Rungwe Tanzania | Exploratory | Qualitative | Qualitative analysis based on in-depth interviews and group discussions | Strong | Name of the studied model: Performance-Based Financing; Nature of the incentivized entity: Health staff is mentioned in general as receiving incentives; Motivation behind model: Promising in improving both health financing and quality of care; Type of Incentive: Only rewards; Performance measure: Absolute measures; Flexibility level: Fixed targets; Payment Channel: Decoupled from usual reimbursement; Certainty of the Targets: Targets set (pre-defined targets); | Tanzania | Dispensaries, health centers, and hospitals | Not specified | Organization-related Outcomes/Experience (informants' perspective on PBF training); | Design a business plan; Involve communities as partners in running the facility; Involve members of the community in the management of health facilities (eg. health committees or governing boards); Have a positive attitude towards patients;  Improve communication; Involve key stakeholders during the design phase, complemented with the need of official policy guidelines for a smooth implementation; Full autonomy by health care providers to manage their health facilities; Use training of personnel to overcome challenges (eg. lack of performance bonuses); | Inadequate funding and poor design of the project; Lack of job description in the informants' workplace (better awareness of roles and responsibilities increases performance and overall quality of care); |
| 58 | Borza, T.; Kaufman, S. R.; Yan, P.; Herrel, L. A.; Luckenbaugh, A. N.; Miller, D. C.; Skolarus, T. A.; Jacobs, B. L.; Hollingsworth, J. M.; Norton, E. C.; Shahinian, V. B.; Hollenbeck, B. K. (2018). Early effect of Medicare Shared Savings Program accountable care organization participation on prostate cancer care | Explanatory | Quantitative | Quantitative analysis based on a 20% Medicare sample | Strong | Name of the studied model: MSSP ACO (3+ years); Provider involvement: Provider not involved (CMS regulates); Nature of the incentivized entity: Group (organization); Motivation behind model: Cost savings & quality of care enhancement; Nº Quality Indicators: Multiple separate indicators; Payment Channel: Coupled with usual reimbursement; Certainty of the Targets: Targets set; Frequency of Incentives: Once a year; | USA | Accountable care organizations | Prostate cancer | Clinical & Cost outcomes (rate of treatment and overtreatment, and payments); | ACO-affiliated practices were more likely to participate in quality improvement collaboratives; Because ACOs are not at risk for penalties, the incentive to decrease costs may be diminished and hospitals initially may focus on quality improvement initiatives (approach could yield maximal financial gains); Reducing overtreatment by referring patients to providers whose treatment patterns align with the goals of the ACO; | - |
| 59 | Lin, M. P.; Revette, A.; Carr, B. G.; Richardson, L. D.; Wiler, J. L.; Schuur, J. D. (2020). Effect of Accountable Care Organizations on Emergency Medicine Payment and Care Redesign: A Qualitative Study | Descriptive | Qualitative | Qualitative analysis based on semistructured interviews with emergency department and accountable care organization leaders | Strong | Name of the studied model: MSSP ACO (3+ years); Provider involvement: Provider not involved (CMS regulates); Nature of the incentivized entity: Group (organization); Motivation behind model: Cost savings & quality of care enhancement; Nº Quality Indicators: Multiple separate indicators; Payment Channel: Coupled with usual reimbursement; Certainty of the Targets: Targets set; Frequency of Incentives: Once a year; | USA | Accountable care organizations | Emergency care (conditions not specified) | Organization-related Outcomes/Experience (key themes and patterns describing the effect of accountable care on emergency medicine care redesign and payment); | - | Lack of defined population; Lack of standardized approaches to group clinically comparable episodes, and high variation in resource use for acute, undifferentiated illness, precluding predictable costs;  Difficulty attributing outcomes to specific providers;  Lack of understanding about risk adjustment; |
| 60 | Constantinou, Panayotis; Sicsic, Jonathan; Franc, Carine (2017). Effect of pay-for-performance on cervical cancer screening participation in France | Exploratory | Quantitative | Quantitative analysis based on a nationally representative permanent sample of health insurance beneficiaries | Strong | Name of the studied model: P4P program (not further specified); Nature of incentivized entity: Bonuses allocated internally to clinical teams whose performance had earned the bonus; Motivation behind model: "Need to simultaneously incentivize quality and appropriateness of care, cost containment and equity in the access of care" Type of Incentive: Only rewards; Nº Quality Indicators: 29 indicators; Performance measure: Absolute measures; Flexibility level: Fixed targets; Payment Channel: Decoupled from usual reimbursement; Certainty of the Targets: Targets set; Frequency of Incentives: Once a year; | France | Hospitals | Cervical cancer | Clinical outcomes (recommended screening participation); | Sufficient size of incentive to significantly affect physicians’ behaviour and to result in a substantial increase in screening participation; Promoting the diversification of smear-test providers through GP and midwives training could also be important levers to enhance cervical cancer screening coverage; | There was no combined effect in areas where both incentives from different payment models were present (no added value); |
| 61 | Friedberg, M. W.; Rosenthal, M. B.; Werner, R. M.; Volpp, K. G.; Schneider, E. C. (2015). Effects of a Medical Home and Shared Savings Intervention on Quality and Utilization of Care | Explanatory | Quantitative | Quantitative analysis based on lists of practices selected by a state contractor | Strong | Name of the studied model: Pennsylvania Chronic Care Initiative (3 years); Motivation behind model: Cost savings & quality of care enhancement; Type of incentive: Both rewards and penalties; Nº Quality Indicators: 14 quality benchmarks; Performance measure: Absolute measures; Flexibility level: Fixed targets; Payment Channel: Each health plan could decide its own method for calculating savings; Frequency of Incentives: Practices receive monhtly payments based on patients; | USA | Medical homes | Diabetes care, and breast and colorectar cancer | Clinical outcomes (performance on 4 process measures, all-cause hospitalizations/emergency department visits, ambulatory care-sensitive emergency department visits, ambulatory visits to specialists, ambulatory primary care visits); | Inclusion of a substantial shared savings incentive, with shared savings bonus payments being contingent on meeting quality measure benchmarks;  Better care management may have contributed to the higher rate of patient retention; Timely availability of data on (encourages and enables primary care practices to contain unnecessary or avoidable utilization in these settings); | - |
| 62 | Ouayogodé, M. H.; Meara, E.; Ho, K.; Snyder, C. M.; Colla, C. H. (2021). Estimates of ACO savings in the presence of provider and beneficiary selection | Explanatory | Quantitative | Quantitative analysis based on Medicare fee-for-service claims data and summary files, the IQVIA OneKey database, and an Academic Medical Centers provider file from the Office of Health and Human Services | Strong | Name of the studied model: Pioneer & MSSP ACO (3+ years); Provider involvement: Provider not involved (CMS regulates); Nature of the incentivized entity: Group (organization); Motivation behind model: Cost savings & quality of care enhancement; Type of incentive: Both rewards and penalties; Payment Channel: Coupled with usual reimbursement; Certainty of the Targets: Targets set; Frequency of Incentives: Once a year; | USA | Accountable care organizations | Not specified | - | - | The set of beneficiaries attributed to ACOs changing dramatically over time, reders it difficult to manage ACO patients to appropriately capture investments; |
| 63 | D'Aunno, T.; Broffman, L.; Sparer, M.; Kumar, S. R. (2018). Factors That Distinguish High-Performing Accountable Care Organizations in the Medicare Shared Savings Program | Descriptive | Qualitative | Qualitative analysis based on in-person interviews with key individuals at each site, key documents, and site-specific memos on ACO objectives | Moderate | Name of the studied model: MSSP ACO (3+ years); Provider involvement: Provider not involved (CMS regulates); Nature of the incentivized entity: Group (organization); Motivation behind model: Cost savings & quality of care enhancement; Payment Channel: Coupled with usual reimbursement; Certainty of the Targets: Targets set; Frequency of Incentives: Once a year; | USA | Accountable care organizations | Not specified | - | Shorter interventions can improve ACO performance, using care coordinators and local, regional health information systems; High-performing ACOs consisted of well-established physician groups with a history of providing cost-effective patient care prior to their involvement; Timely feedback of performance data and access to data; Need for physician-based hospitals to collaborate with local hospitals (more likely to occur when an ACO has strong, long-standing, and highly trusted physician leadership); | Incentives in the MSSP might be too weak and thus not motivate the organizations to take advantage of their capabilities; Effective care management systems may take years to reach full potential; |
| 64 | Karim, S. A.; Nevola, A.; Morris, M. E.; Tilford, J. M.; Chen, H. F. (2021). Financial Performance of Hospitals in the Appalachian Region Under the Hospital Readmissions Reduction Program and Hospital Value-Based Purchasing Program | Explanatory | Quantitative | Quantitative analysis based on Centers for Medicare and Medicaid Services (CMS) Hospital Cost Report Information System, CMS Impact File, Appalachian Regional Authority website, CMS Provider of Services File, Area Health Resource File, American Community Survey, and State Medicaid Expansion dates from Advisory Board website | Moderate | Name of the studied model: The Hospital Readmission and Reduction Program and Hospital Value-Based Purchasing Program (3+ years); Provider involvement: Provider not involved (CMS regulates); Nature of the incentivized entity: Group (organization); Motivation behind model: Financial; Type of Incentive: Both rewards and penalties; Nº Quality Indicators: Multiple separate indicators; Performance measure: Absolute measures; Flexibility level: Over time, the penalty has increased to the maximum CMS established rate and weighting; Type & Size of payment: Monetary gain or lost up until 1% (increased to 2% in 2017); Payment Channel: Coupled with usual reimbursement; Certainty of the Targets: Different year by year; Frequency of Incentives: Once a year; | USA | Hospitals | Heart failure, pneumonia, acute myocardial infraction, elective tital knee and hip replacement, and COPD | Cost outcomes (operating and total margin); | - | Exclusion of socioeconomic status in the risk adjustment models used to assess hospital performance; |
| 65 | Ouayogodé, M. H.; Meara, E.; Chang, C. H.; Raymond, S. R.; Bynum, J. P. W.; Lewis, V. A.; Colla, C. H. (2018). Forgotten patients: ACO attribution omits those with low service use and the dying | Explanatory | Quantitative | Quantitative analysis based on characteristics of Medicare fee-for-service beneficiaries | Strong | Name of the studied model: Pioneer & MSSP ACO & other non-ACO programs (3+ years); Provider involvement: Provider not involved (CMS regulates); Nature of the incentivized entity: Group (organization); Motivation behind model: Cost savings & quality of care enhancement; Type of Incentive: Both rewards and penalties; Nº Quality Indicators: Multiple separate indicators; Payment Channel: Coupled with usual reimbursement; Certainty of the Targets: Targets set; Frequency of Incentives: Once a year; | USA | Accountable care organizations | Not specified | - | Integrate regional factors when calculating cost benchmarks; Incentivize ACOs to select healthy patients for primary care visits; Involvement of hospice and palliative care in new payment initiatives may improve quality of care and potentially reduce costs; | - |
| 66 | Kim, Y.; Thirukumaran, C. P.; Li, Y. (2018). Greater Reductions in Readmission Rates Achieved by Urban Hospitals Participating in the Medicare Shared Savings Program | Explanatory | Quantitative | Quantitative analysis based on the Centers for Medicare and Medicaid Services Hospital Compare | Moderate | Name of the studied model: MSSP ACO (3+ years); Provider involvement: Provider not involved (CMS regulates); Nature of the incentivized entity: Group (organization); Motivation behind model: Cost savings & quality of care enhancement; Nº Quality Indicators: Multiple separate indicators; Payment Channel: Coupled with usual reimbursement; Certainty of the Targets: Targets set; Frequency of Incentives: Once a year; | USA | Acute care hospitals | Pneumonia, heart failure and other conditions (not specified) | Clinical outcomes (readmission rates for Medicare patients for heart failure and pneumonia); | Design clinical practices that specifically targets frail elderly patients for better care; Financial incentives related to overall costs of patient care and performance on quality measures; Improved coordination of care between hospitals and postacute care providers when their practices and resources are better integrated under the ACOs; | - |
| 67 | Carroll, N. W.; Clement, J. P. (2020). Hospital Performance in the First 6 Years of Medicare's Value-Based Purchasing Program | Explanat ory | Quantitative | Quantitative analysis based on Centers for Medicare and Medicaid Services (CMS) Hospital Compare database, the American Hospital Association’s Annual Survey of Hospitals, the CMS Impact file, Medicare Cost Reports, the Area Health Resource File, and the American Nurses Credentialing Center | Moderate | Name of the studied model: Value-based purchasing program (not specified further) (6 years); Provider involvement: Provider not involved (CMS regulates); Motivation behind model: Cost savings & quality of care enhancement; Type of Incentive: Both rewards and penalties; Nº Quality Indicators: Hospital performance assessed along multiple dimensions of quality, and each dimension assessed using multiple measures Performance measure: Hospital's performance is determined relative to other hospitals' improvement and to its past performance; Payment Channel: Percentage of Medicare's operating payments; Certainty of the Targets: Change every year; Frequency of Incentives: Once a year; | USA | Hospitals | Not specified | - | Found that increases in nurse staffing levels were associated with good performance; | Weak incentives, and lack resources and ability to improve (financial incentives the VBP program poses not sufficient to induce a response from for-profit hospitals);  Structures of the program may be found too complex; Changes in the domains that contribute to VBP program scores (lack of consistency); |
| 68 | Blustein, J.; Borden, W. B.; Valentine, M. (2010). Hospital performance, the local economy, and the local workforce: findings from a US National Longitudinal Study | Explanatory | Quantitative | Quantitative analysis based on the Hospital Compare website, and Health Resources and Services Administration’s Area Resource File | Weak | Name of the studied model: Value-Based Purchasing Initiative; Provider involvement: Provider not involved (CMS regulates); Motivation behind model: Cost savings & quality of care enhancement; Type of Incentive: Both rewards and penalties, since a portion of revenues is withheld and then returned, conditional on ability to meet quality targets; Performance measure: Absolute measures; Payment Channel: Coupled with usual reimbursement; | USA | Hospitals | Acute myocardial infarction and heart failure | - | US hospitals operating in locations with richer economic and human resources attained significantly higher clinical process scores than those located in less advantaged areas (location may be a barrier or facilitator for the payment model, since clinical scores are associated with the models); | Initially, low-performing hospitals show the greatest increases, because if they are less efficient, the progress is higher; |
| 69 | Tory, H. Hogan; Christy Harris, Lemak; Nataliya, Ivankova; Larry, R. Hearld; Jack, Wheeler; Nir, Menachemi (2018). Hospital Vertical Integration Into Subacute Care as a Strategic Response to Value-Based Payment Incentives, Market Factors, and Organizational Factors: A Multiple-Case Study | Exploratory | Qualitative | Case study, with qualitative analysis based on in-depth semistructured interviews with health system executives, information posted on health system websites, annual reports, news articles about health system and organziational strategy, the American Hospital Association's Annual Survey, and Center for Medicare and Medicaid services | Moderate | Name of the studied model: Not specified; Provider involvement: Provider not involved (CMS regulates); Nature of the incentivized entity: Group (organization); Certainty of the Targets: Targets set; | USA | Health systems | Not specified | - | Policy makers need to understand the organizational and market environments, as well as evaluate the current value-based payment policies to ensure they accurately spread the financial risk to all providers across the continuum; Hospitals are vertically integrating in response to the value-based payment incentives; | - |
| 70 | Chien, A. T.; Schiavoni, K. H.; Sprecher, E.; Landon, B. E.; McNeil, B. J.; Chernew, M. E.; Schuster, M. A. (2016). How Accountable Care Organizations Responded to Pediatric Incentives in the Alternative Quality Contract | Descriptive | Qualitative | Qualitative analysis based on the Linked the Massachusetts Health Quality Partners Provider 2011 Database and the American Hospital Association 2009 Annual Survey Database, as well as semistructured interviews with leaders of ACOs | Moderate | Name of the studied model: Alternative Quality Contract & BlueCross BlueShield of Massachusetts’s global payment arrangement (3+ years); Provider involvement: Provider not involved; Nature of the incentivized entity: Group (organization); Motivation behind model: Improve quality of care; Type of Incentive: Both rewards and penalties, similar to Pioneer ACO; Nº Quality Indicators: 64 total measures; Performance measure: Absolute measures; Payment Channel: Coupled with usual reimbursement; Certainty of the Targets: Targets set; Frequency of Incentives: Once a year; | USA | Hospitals | Pediatric care (conditions not specified) | Organization-related Outcomes/Experience (leaders' reports on quality improvement efforts and spending patterns); | In ACOs with basic or substantial pediatric infrastructures, pediatric incentives were robust enough to invest in building pediatric QI systems and in better understanding pediatric spending patterns | - |
| 71 | Coulibaly, A.; Gautier, L.; Zitti, T.; Ridde, V. (2020). Implementing performance-based financing in peripheral health centres in Mali: what can we learn from it? | Explanatory | Qualitative | Qualitative analysis based on semi-structured interviews, informal interviews and non-participant observation | Strong | Name of the studied model: PBF pilot program; Motivation behind model: Patient benefit & Financial interest; Type of Incentive: Only rewards; Nº Quality Indicators: Separate and weighted measures; Performance measure: Absolute measures; Payment Channel: Decoupled from usual reiumbursement; Certainty of the Targets: Targets set (quantity indicators purchased at a fixed price); | Mali | Health community centers and reference health centers | Maternal and pediatric care (conditions not specified) | Organization-related Outcomes/Experience (leadership exercised, reported information regarding intervention); | Reducing delays in incentive payments, communicating effectively and strengthening health workers’ understanding of the PBF system are likely to improve motivation and overall performance; Removing practical norms and professional cultures, that govern the actual behaviour of employees, from official norms; Strong Leadership (increases engagement); | Implementation deficiencies, such as late payment and access difficulties, poses a series of problems that limit the motivational effects of incentives |
| 72 | Hayen, Arthur; van den Berg, Michael Jack; Struijs, Jeroen Nathan; Westert, Gerard Pieter (2021). Dutch shared savings program targeted at primary care: Reduced expenditures in its first year | Explanatory | Quantitative | Quantitative analysis based on Menzis claims and enrollment data, and a survey | Weak | Name of the studied model: MSSP ACO (3+ years); Provider involvement: Provider not involved (CMS regulates); Nature of incentivized entity: GPs incentivized; Motivation behind model: Cost savings & quality of care enhancement; Type of Incentive: Only rewards; Nº Quality Indicators: Multiple separate indicators; Performance measure: The resulting payout to GPs was a function of both absolute performance and improvement on performance; Flexibility level: Fixed targets; Certainty of the Targets: Targets set; Frequency of Incentives: Once a year; | Netherlands | National chain of primary care centers | Asthma, diabetes mellitus II, and cardiovascular conditions (conditions not specified) | Clinical & Cost outcomes & Patient-reported Outcomes/Experience (health care expenditures, patient satisfaction, and quality indicators); | Stronger quality incentives can be introduced by putting more weight on improvement, or by sharing savings only conditionally on achieving a combined set of quality targets;  Rewards to exclude no shows, for savings in primary care, and for savings by other providers; Sharing of expenditure data (volumes of spending, prices and benchmarks); | - |
| 73 | Larson, B. K.; Van Citters, A. D.; Kreindler, S. A.; Carluzzo, K. L.; Gbemudu, J. N.; Wu, F. M.; Nelson, E. C.; Shortell, S. M.; Fisher, E. S. (2012). Insights from transformations under way at four Brookings-Dartmouth accountable care organization pilot sites | Descriptive | Qualitative | Case study, with qualitative analysis based on semistructured individual and group interviews with staff, observed meetings, toured practices, and reviewed documentation. | Moderate | Name of the studied model: Brookings-Dartmouth ACO Collaborative (3+ years); Provider Involvement: Staff worked with state and federal policy makers, national payers, and the pilot sites; Nature of the incentivized entity: Group (organization); Motivation behind model: Improve quality of care; Type of Incentive: No risk in year 1, and transition to risk bearing in year 2; Payment Channel: Coupled with usual reimbursement; Certainty of the Targets: Targets set; Frequency of Incentives: Once a year; | USA | Medical group/independent practice association, independent practice association, and community hospital working with independent provider groups | Not specified | - | Sharing and analysis of historical claims data for ACO-attributed patients (helps reducing uncertainty surrounding setting performance targets for new patient populations); Linking providers to learning networks where payers and providers can address common technical issues (capture lessons from other adopters); | Antitrust and anti-kickback statutes, which limits cooperation between payers and providers; Commercial payers and CMS need to recognize that the transition from existing fragmented fee-for-service to coordinated models of care with shared risk take time; |
| 74 | Cornell, Tatiana (2020). Leadership skills essential in the value-based care era | Descriptive | Qualitative | Case study, with qualitative analysis based on interviews, focus group discussion and archival record data | Strong | Name of the studied model: MSSP ACO (3+ years); Provider involvement: Provider not involved (CMS regulates); Nature of the incentivized entity: Group (organization); Motivation behind model: Cost savings & quality of care enhancement; Nº Quality Indicators: Multiple separate indicators; Payment Channel: Coupled with usual reimbursement; Certainty of the Targets: Targets set; Frequency of Incentives: Once a year; | USA | Accountable care organizations | Not specified | - | Leadership skills to align all internal and external stakeholders to promote MSSP ACO effectiveness (ability to envision and implement the most effective strategies, leverage subject matter expertise, communicate benefits effectively, lead a change of movement, collaborate with all stakeholders, emphasize the importance of technology, and inspire provider to buy into the vision of the value-based care delivery); | - |
| 75 | Cassandra, Leighton; Evan, Cole; A. Everette James; Julia, Driessen (2019). Medicare Shared Savings Program ACO network comprehensiveness and patient panel stability | Explanatory | Quantitative | Quantitative analysis based on Medicare fee-for-service beneficiary and provider files, and the US Census and the ACO Public Use File | Strong | Name of the studied model: MSSP ACO (3+ years); Provider involvement: Provider not involved (CMS regulates); Nature of the incentivized entity: Group (organization); Motivation behind model: Cost savings & quality of care enhancement; Nº Quality Indicators: Multiple separate indicators; Payment Channel: Coupled with usual reimbursement; Certainty of the Targets: Targets set; Frequency of Incentives: Once a year; | USA | Accountable care organizations | Not specified | - | Stable patient assignment year to year improves network comprehensiveness; ACOs increase the accessibility and availability of contracted providers, which limits the need to seek care outside of the ACO’s network and reduces the threat of panel instability; | - |
| 76 | Markovitz, A. A.; Rozier, M. D.; Ryan, A. M.; Goold, S. D.; Ayanian, J. Z.; Norton, E. C.; Peterson, T. A.; Hollingsworth, J. M. (2020). Low-Value Care and Clinician Engagement in a Large Medicare Shared Savings Program ACO: a Survey of Frontline Clinicians | Explanatory | Quantitative | Quantitative analysis based on a survey | Moderate | Name of the studied model: Physician Organization of Michigan MSSP (3+ years); Provider involvement: Provider not involved (CMS regulates); Nature of the incentivized entity: Group (organization); Motivation behind model: Cost savings & quality of care enhancement; Payment Channel: Receive percentage of savings; Certainty of the Targets: Targets set; Frequency of Incentives: Once a year; | USA | Accountable care organizations | Not specified | Organization-related Outcomes/Experience (clinicians' decision to join ACOs/participation in decisions, and awareness about components of ACOs and implementation of its components); | Greater savings among physician-led ACOs than among hospital-led ACOs; ACO efforts to lower spending may benefit from promoting patient education and clinician-patient conversations regarding high-value healthcare decisions; | Clinicians' unawareness and unengagement with ACO objectives and activities (eg. the new organizational financial incentives created by the MSSP); |
| 77 | Modi, P. K.; Kaufman, S. R.; Caram, M. E.; Ryan, A. M.; Shahinian, V. B.; Hollenbeck, B. K. (2021). Medicare Accountable Care Organizations and the Adoption of New Surgical Technology | Explanatory | Quantitative | Quantitative analysis based on a 20% sample of national Medicare claims | Strong | Name of the studied model: MSSP ACO (3+ years); Provider involvement: Provider not involved (CMS regulates); Nature of the incentivized entity: Group (organization); Motivation behind model: Cost savings & quality of care enhancement; Payment Channel: Coupled with usual reimbursement; Certainty of the Targets: Targets set; Frequency of Incentives: Once a year; | USA | Accountable care organizations | Abdominal aortic aneurysm repair, aortic valve replacement, carotid endarterectomy or stent, lung lobectomy, colectomy, and prostatectomy (conditions not specified) | Clinical outcomes (rate of surgical treatment); | - | Incentive to reduce spending may not be large enough to overcome the incentive to expand surgical care and the use of expensive new technology; Hospitals may not know the proportion of surgical patients who will ultimately be attributed to their ACO; |
| 78 | Huang, N.; Raji, M.; Lin, Y. L.; Chou, L. N.; Kuo, Y. F. (2021). Nurse Practitioner Involvement in Medicare Accountable Care Organizations: Association With Quality of Care | Explanatory | Quantitative | Quantitative analysis based on ACO provider/beneficiary files, Medicare claims, and ACO performance data | Moderate | Name of the studied model: MSSP ACO (3+ years); Provider involvement: Provider not involved (CMS regulates); Nature of the incentivized entity: Group (organization); Motivation behind model: Cost savings & quality of care enhancement; Nº Quality Indicators: 17 measures; Payment Channel: Coupled with usual reimbursement; Certainty of the Targets: Targets set; Frequency of Incentives: Once a year; | USA | Accountable care organizations | Diabetes, chronic obstructive pulmonary disease, and heart failure | - | Involve nurse practicioners more (give them more responsibility as well, eg. allocate them to "healthier" patients, so that medical doctors can stay with the "sicker", more complex patients); | - |
| 79 | Zhu, X.; Mueller, K.; Huang, H.; Ullrich, F.; Vaughn, T.; MacKinney, A. C. (2019). Organizational Attributes Associated With Medicare ACO Quality Performance | Explanatory | Quantitative | Quantitative analysis based on data from the Centers for Medicare and Medicaid Services (and additional sources) | Moderate | Name of the studied model: MSSP ACO (3+ years); Provider involvement: Provider not involved (CMS regulates); Nature of the incentivized entity: Group (organization); Motivation behind model: Cost savings & quality of care enhancement; Nº Quality Indicators: 33 quality measures and an overall quality score; Payment Channel: Coupled with usual reimbursement; Certainty of the Targets: Targets set; Frequency of Incentives: Once a year; | USA | Accountable care organizations | Not specified | - | Advance Payment Model (upfront and monthly payments to ACOs with limited resources and experiences); Larger hospital systems serving larger panels may have invested more substantially in quality initiatives and developed better processes for measuring quality and providing actionable feedback to front-line providers; Physician groups serving smaller panels may excel in reducing costs by improving primary care and reducing avoidable utilization; | Model, still on early stage of implementation, needs time; |
| 80 | Zhang, H.; Wernz, C.; Hughes, D. R. (2018). Modeling and designing health care payment innovations for medical imaging | Explanatory | Mixed Methods | Quantitative analysis based on a mathematical model to analyze and improve MSSP | Weak | Name of the studied model: MSSP ACO (3+ years); Provider involvement: Provider not involved (CMS regulates); Nature of the incentivized entity: Group (organization); Motivation behind model: Cost savings & quality of care enhancement; Payment Channel: Coupled with usual reimbursement; Certainty of the Targets: Targets set; Frequency of Incentives: Once a year; | USA | Accountable care organizations | Not specified | - | If the cost benchmark set by CMS is too high, hospitals will keep all incentive to themselves. When reducing the cost benchmark below a certain threshold, hospitals begin to share the incentive with physicians. The greater the sharing rate, the stronger the motivation for physicians to reduce testing thresholds; | - |
| 81 | Olafsdottir, A. E.; Mayumana, I.; Mashasi, I.; Njau, I.; Mamdani, M.; Patouillard, E.; Binyaruka, P.; Abdulla, S.; Borghi, J. (2014). Pay for performance: an analysis of the context of implementation in a pilot project in Tanzania | Descriptive | Qualitative | Qualitative analysis based on in-depth interviews and four focus group discussions, as well as survey of facilities and health workers | Strong | Name of the studied model: P4P (not further specified); Nature of incentivized entity: Different staff eligible to different bonuses; Motivation behind model: Motivation behind model: Cost savings & quality of care enhancement; Type of Incentive: Only rewards; Nº Quality Indicators: Multiple separate indicators; Performance measure: Absolute measures; Flexibility level: Fixed targets; Payment Channel: Decoupled from usual reiumbursement; Certainty of the Targets: Pay-outs made according to the previous cycle; Frequency of Incentives: Pay-outs made every 6 months; | Tanzania | Dispensaries, health centres and hospitals | Not specified | - | - | Shortages of resources (eg. staff), limited supplies (funds and staff supply from schools), and unfavourable community preferences; Workload of staff higher than contracted for (not reflected in the salary), which is not reflected on the salary; Payments are delayed and overtime and eligible allowances are not always paid, causing demotivation; |
| 82 | Aditi, P. Sen; Lena, M. Chen; Lok Wong, Samson; Arnold, M. Epstein; Karen, E. Joynt Maddox (2018). Performance in the Medicare Shared Savings Program by Accountable Care Organizations Disproportionately Serving Dual and Disabled Populations | Explanatory | Quantitative | Quantitative analysis based on Centers for Medicare and Medicaid Services’ ACO Beneficiary-level Research Identifiable File, and the Master Beneficiary Summary File | Strong | Name of the studied model: MSSP ACO (3+ years); Provider involvement: Provider not involved (CMS regulates); Nature of the incentivized entity: Group (organization); Motivation behind model: Cost savings & quality of care enhancement; Type of Incentive: Not just Track 1, with both rewards and penalties; Nº Quality Indicators: Multiple separate indicators; Payment Channel: Coupled with usual reimbursement; Certainty of the Targets: Targets set; Frequency of Incentives: Once a year; | USA | Accountable care organizations | Not specified | - | Cost targets calculated separately for several vulnerable populations, specifically those who are dually enrolled in both Medicaid and Medicare and those who are disabled; MSSP spending benchmarks for ACOs’ second and subsequent agreement periods based on a combination of the ACO’s own historical spending and regional spending; | - |
| 83 | Han, M. A.; Clarke, R.; Ettner, S. L.; Steers, W. N.; Leng, M.; Mangione, C. M. (2016). Predictors of Out-of-ACO Care in the Medicare Shared Savings Program | Explanatory | Quantitative | Quantitative analysis based on data from Centers for Medicare and Medicaid ACO Program Claim and Claim Line Feed dataset | Strong | Name of the studied model: MSSP ACO (3+ years); Provider involvement: Provider not involved (CMS regulates); Nature of the incentivized entity: Group (organization); Motivation behind model: Cost savings & quality of care enhancement; Type of Incentive: Track 1, only rewards; Nº Quality Indicators: Multiple separate indicators; Payment Channel: Coupled with usual reimbursement; Certainty of the Targets: Targets set; Frequency of Incentives: Once a year; | USA | Accountable care organizations | Not specified | Cost outcomes (out-of-ACO expenditures); | Ethnic minorities have a higher trust in familiar providers than white patients; Commorbidities are better taken care in an ACO, because of care coordination; | MSSP’s open-network structure might help patients seek the highest-quality care, but also decrease efficiency, since patients with comorbidities are the ones that mostly need coordination; |
| 84 | Chukmaitov, A.; Harless, D. W.; Bazzoli, G. J.; Muhlestein, D. B. (2019). Preventable Hospital Admissions and 30-Day All-Cause Readmissions: Does Hospital Participation in Accountable Care Organizations Improve Quality of Care? | Explanatory | Quantitative | Quantitative and Quantitative analysis based on the American Hospital Association survey, Health Information and Management System Society information technology data, databases on participants of CMS-initiated pilots and demonstrations, the Area Health Resource File, and data from Leavitt Partners | Moderate | Name of the studied model: Pioneer & MSSP ACO (3+ years); Provider involvement: Provider not involved (CMS regulates); Nature of the incentivized entity: Group (organization); Motivation behind model: Cost savings & quality of care enhancement; Type of Incentive: Both rewards and penalties; Payment Channel: Coupled with usual reimbursement; Certainty of the Targets: Targets set; Frequency of Incentives: Once a year; | USA | Accountable care organizations | Chronic obstructive pulmonary disease, asthma, chronic heart failure, and complications of diabetes | Clinical outcomes (preventable hospitalizations for Chronic obstructive pulmonary disease, asthma, diabetes complications and preventable congestive heart failure, and 30-day readmissions); | Physician-led ACOs generated greater savings relative to hospital-led ACOs (stronger incentives to reduce hospital utilization that may have been difficult for hospitals to implement without hurting their bottom line); | ACO programs may have provided only weak rewards for hospitals to change their business model from generating volume of care to focusing on value-based care; Finally, there may not have been sufficient time to implement organizational changes, transform the practice of medicine, and fully transition to value-based payments; Poor information sharing and lack of interoperability of electronic health records across settings (prevent coordinated care); |
| 85 | Albright, B. B.; Lewis, V. A.; Ross, J. S.; Colla, C. H. (2016). Preventive Care Quality of Medicare Accountable Care Organizations: Associations of Organizational Characteristics With Performance | Explanatory | Quantitative | Quantitative analysis based on data for Medicare ACOs publicly available from The Centers for Medicare and Medicaid Services, and data from the National Survey of ACOs | Strong | Name of the studied model: Pioneer & MSSP ACO (3+ years); Provider involvement: Provider not involved (CMS regulates); Nature of the incentivized entity: Group (organization); Motivation behind model: Cost savings & quality of care enhancement; Type of Incentive: Both rewards and penalties; Nº Quality Indicators: Multiple singular quality indicators and composite scores; Payment Channel: Coupled with usual reimbursement; Certainty of the Targets: Targets set; Frequency of Incentives: Once a year; | USA | Accountable care organizations | Not specified | - | Participation in the Medicare Advanced Payment Model (upfront financial investment directly to smaller MSSP ACOs); Better performance by these ACOs on preventive care may be driven by the investment itself; Balanced ACO workforce (PCPs and specialists); | Having more patients under different contracts in addition to the ACO may greatly complicate quality measurement; Evidence has shown persistence of racial disparities in quality for Medicare ACOs, and that minority patients face added practical barriers to obtaining care; |
| 86 | Herrel, L. A.; Ayanian, J. Z.; Hawken, S. R.; Miller, D. C. (2017). Primary care focus and utilization in the Medicare shared savings program accountable care organizations | Explanatory | Quantitative | Quantitative analysis based on Centers for Medicare and Medicaid Services Shared Savings Program public-use file | Moderate | Name of the studied model: MSSP ACO (3+ years); Provider involvement: Provider not involved (CMS regulates); Nature of the incentivized entity: Group (organization); Motivation behind model: Cost savings & quality of care enhancement; Payment Channel: Coupled with usual reimbursement; Certainty of the Targets: Targets set; Frequency of Incentives: Once a year; | USA | Accountable care organizations | Not specified | - | Contextual (e.g., ACO size) and patient factors (e.g. comorbidities) that vary across MSSP organizations (may affect shared savings), and need to be considered in the model; | - |
| 87 | Fraze, T. K.; Lewis, V. A.; Tierney, E.; Colla, C. H. (2018). Quality of Care Improves for Patients with Diabetes in Medicare Shared Savings Accountable Care Organizations: Organizational Characteristics Associated with Performance | Explanatory | Quantitative | Quantitative analysis based on National Survey of ACOs, and publicly available data from the Centers for Medicare & Medicaid Services | Moderate | Name of the studied model: MSSP ACO (3+ years); Provider involvement: Provider not involved (CMS regulates); Nature of the incentivized entity: Group (organization); Motivation behind model: Cost savings & quality of care enhancement; Nº Quality Indicators: Separate and composite measures; Payment Channel: Coupled with usual reimbursement; Certainty of the Targets: Targets set; Frequency of Incentives: Once a year; | USA | Accountable care organizations | Diabetes | Clinical outcomes (ACO performance in diabetes care); | More preparation (offering more comprehensive services) is associated with a better initial performance; | ACOs with better year 1 performance had less improvement between performance years (overcome initial differences in performance within a relatively short time frame, but there is a ceiling effect, making it hard to continue improving to those already at a higher performance) |
| 88 | Gill, B. S.; Beriwal, S.; Rajagopalan, M. S.; Wang, H.; Hodges, K.; Greenberger, J. S. (2015). Quantitative evaluation of radiation oncologists' adaptability to lower reimbursing treatment programs | Explanatory | Quantitative | Quantitative analysis based on a web-based survey to radiation oncologists | Weak | Name of the studied model: Bundled payment program; Provider involvement: Provider not involved (CMS regulates); Nature of the incentivized entity: Group (organization); Motivation behind model: Patient benefit & Financial interest; Payment Channel: Coupled with usual reimbursement; | USA | Comprehensive cancer center network | Cancer | Organization-related Outcomes/Experience (physician’s willingness to adopt/adoption of LRO); | By educating physicians about reimbursement costs, physicians are more inclined to alter practice patterns; | Concerns remain that value-based models may be challenging to apply in outpatient settings, where prospective payments can lead to undertreatment (“stinting on care”) and selection of healthier patients (“cherry picking”); |
| 89 | Mc, Williams Jm; Hatfield, L. A.; Landon, B. E.; Chernew, M. E. (2020). Savings or Selection? Initial Spending Reductions in the Medicare Shared Savings Program and Considerations for Reform | Explanatory | Quantitative | Quantitative analysis based on Medicare claims data | Strong | Name of the studied model: MSSP ACO (3+ years); Provider involvement: Provider not involved (CMS regulates); Nature of the incentivized entity: Group (organization); Motivation behind model: Cost savings & quality of care enhancement; Type of Incentive: Not just Track 1, with both rewards and penalties; Nº Quality Indicators: Multiple separate indicators; Payment Channel: Coupled with usual reimbursement; Certainty of the Targets: Targets set; Frequency of Incentives: Once a year; | USA | Accountable care organizations | Not specified | Cost outcomes (annual gross savings); | Strengthen incentives for ACOs to lower spending without discouraging participation; A voluntary program may require a longer transition period in which benchmarks are based entirely on ACOs’ baseline spending (no regional component) and updated annually without rebasing; Remove the link between benchmarks and prior savings; In a one-sided contract without downside risk; | Incentives for ACOs to lower spending have been weak to date (benchmarks have been rebased after each three-year contract period to reflect ACOs’ most recent spending); ACO-specific historical spending component of benchmarks continues to be rebased every contract period, and the initial 10%-25% weight given to the regional component discourages participation by providers with high spending; Temporarily increasing spending (earn a future bonus without exerting further effort as its spending falls to its prior level); |
| 90 | DeLia, D.; Hoover, D.; Cantor, J. C. (2012). Statistical uncertainty in the Medicare shared savings program | Explanatory | Quantitative | Quantitative analysis based on a model and calculation of probabilities of shared savings | Weak | Name of the studied model: MSSP ACO (3+ years); Provider involvement: Provider not involved (CMS regulates); Nature of the incentivized entity: Group (organization); Motivation behind model: Cost savings & quality of care enhancement; Type of Incentive: Not just Track 1, with both rewards and penalties; Nº Quality Indicators: Multiple separate indicators; Payment Channel: Coupled with usual reimbursement; Certainty of the Targets: Targets set (risk and demographically adjusted); Frequency of Incentives: Once a year; | USA | Accountable care organizations | Not specified | Cost outcomes (probability of ACO being inappropriately rewarded/paying an undeserved penalty, real savings rate); | - | Calculations suggest that expected ACO income over the 3-year agreement period would be insufficient to recover the start-up costs costs in many cases, particularly when an ACO is small; An ACO that reduces spending relative to a local growth trend would find it harder to be rewarded for doing so if local growth in spending is substantially faster than the national growth rate; ACOs that become too large could limit choices and care options available to patients, and might overlook very local and specialized needs of specific communities; Smaller ACOs might operate at a scale that is too small to generate hoped for efficiencies in care management; Low spending areas are already providing care very efficiently, making it difficult to achieve continued savings under the MSSP; |
| 91 | Kim, Y.; Thirukumaran, C.; Temkin-Greener, H.; Hill, E.; Holloway, R.; Li, Y. (2021). The Effect of Medicare Shared Savings Program on Readmissions and Variations by Race/Ethnicity and Payer Status (December 9, 2020) | Explanatory | Quantitative | Quantitative analysis based on 2016 MSSP ACO Participants file from the CMS website | Moderate | Name of the studied model: MSSP ACO (3+ years); Provider involvement: Provider not involved (CMS regulates); Nature of the incentivized entity: Group (organization); Motivation behind model: Cost savings & quality of care enhancement; Type of Incentive: Only rewards; Nº Quality Indicators: Multiple separate indicators; Flexibility level: Attainment of certain set benchmarks; Payment Channel: Coupled with usual reimbursement; Certainty of the Targets: Targets set; Frequency of Incentives: Once a year; | USA | Accountable care organizations | Ischemic stroke, hip fracture, and elective total joint arthroplasty | Clinical outcomes & Organization-related Outcomes/Experience (readmission rate for ischemic stroke and hip fracture, racial/ethnic disparities); | - | MSSP ACOs may take at least 3 years to achieve reduced readmissions, and may increase disparities by payer status (need time to redesign clinical practices, and improve discharge planning and coordinated care); MSSP had no effect on existing racial/ethnic disparities in readmission rates (patients with less social support and more likely to lack access to primary care after hospital discharge); |
| 92 | Murray, G. F.; D'Aunno, T.; Lewis, V. A. (2018). Trust, Money, and Power: Life Cycle Dynamics in Alliances Between Management Partners and Accountable Care Organizations | Explanatory | Qualitative | Qualitative analysis based on on semistructured interviews and observational data from site visits | Moderate | Name of the studied model: Not specified; Provider involvement: Provider not involved; Nature of the incentivized entity: Group (organization); Payment Channel: Coupled with usual reimbursement; Certainty of the Targets: Targets set; Frequency of Incentives: Once a year; | USA | Accountable care organizations | Not specified | - | Medicare's Advance Payment Program might help with lacking capital or technical expertise that ACOs might run into; Activelly address the sources of tensions from the partnership; | In case tensions are ignored, they will weaken the partnership, which will end up dissolving it; |
| 93 | Kristensen, Søren Rud; Bech, Mickael; Lauridsen, Jørgen T. (2016). Who to pay for performance? The choice of organisational level for hospital performance incentives | Explanatory | Quantitative | Case study, with quantitative analysis based on performance of hospital departments | Weak | Name of the studied model: P4P program (not further specified); Nature of the incentivized entity: Article tests incentives at hospital vs department-level; Type of Incentive: Both rewards and penalties; Flexibility level: There's a threshold, but the further away from it, the bigger the bonus or penalty; Payment Channel: Decoupled from usual reiumbursement; Frequency of Incentives: Once a year; | Denmark | Hospitals | Not specified | - | Results suggest that payers can improve the effectiveness of P4P payments by distributing payments to the department level rather than the hospital level; | - |
| 94 | Kristensen, Søren Rud; McDonald, Ruth; Sutton, Matt (2013). Should pay-for-performance schemes be locally designed? evidence from the commissioning for quality and innovation (CQUIN) framework | Explanatory | Qualitative | Case study, with qualitative analysis based on interviews and observed meetings | Moderate | Name of the studied model: CQUIN payment framework; Provider Involvement: Providers involved in setting goals and indicators; Motivation behind model: Improve patient outcomes; Type of incentive: Proportion of provider income conditional on achieving quality indicators; Type & Size of payments: Incentive increased from 0,5% of a provider's annual contract income in 2009/2010 to 1,5% in 2010/2011 and to 2.5% from April 2012; Payment Channel: Percentage of providers' annual contract; Certainty of the Targets: Targets set; Frequency of Incentives: Once a year; | UK | Acute care scheme | Not specified | Organization-related Outcomes/Experience (local enthusiasm around schemes, identification of variation in local needs and priorities for quality improvement); | - | There cannot be a guarantee that there is local knowledge about setting thresholds, defining good performance indicators, setting reward levels, etc...; Locally developed schemes did not generate the anticipated local enthusiasm and the type of prioritization of indicators; Local strategic and clinical input into the design of P4P schemes should be kept separate from the technical design process (eg.involves defining indicators, agreeing thresholds, and setting prices). These tasks require expertise that is unlikely to exist in each locality; Regularly having to negotiate new goals and schemes leaves little time for engaging clinicians; |
| 95 | Hsu, J.; Vogeli, C.; Price, M.; Brand, R.; Chernew, M. E.; Mohta, N.; Chaguturu, S. K.; Weil, E.; Ferris, T. G. (2017). Substantial Physician Turnover And Beneficiary 'Churn' In A Large Medicare Pioneer ACO | Explanatory | Quantitative | Quantitative analysis based on data from databases from the health system | Strong | Name of the studied model: Pioneer ACO (3+ years); Provider involvement: Provider not involved (CMS regulates); Nature of the incentivized entity: Group (organization); Motivation behind model: Cost savings & quality of care enhancement; Type of Incentive: Both rewards and penalties; Nº Quality Indicators: Multiple separate indicators; Payment Channel: Coupled with usual reimbursement; Certainty of the Targets: Targets set; Frequency of Incentives: Once a year; | USA | Accountable care organizations | Not specified | Organization-related Outcomes/Experience (physician turnover); | Coordinated and standardized efforts to define shared-risk goals and measurements across payers; Concentrate care within a smaller number of physicians or focus on physicians who have a higher volume of ACO-eligible beneficiaries; | Any single payer will have limited penetration at the physician level, which could dilute the impact of the ACO on its financial and quality targets (because of few beneficiaries per physician). With the small numbers, the distribution of high-spending beneficiaries also is skewed such that a few physicians appeared to have the sickest beneficiaries, while many appeared to have mostly beneficiaries with modest spending; |
| 96 | Tanenbaum, J. E.; Votruba, M.; Einstadter, D.; Love, T. E.; Cebul, R. D. (2021). Adoption of Health System Innovations: Evidence of Urban-Rural Disparities from the Ohio Primary Care Marketplace | Explanatory | Quantitative | Quantitative analysis based on data from the Ohio Department of Health, accredited sites, Medicare Shared Savings Program ACO Provider-level Research Identifiable File, The Ohio Health Information Partnership, and Area Health Resource File | Strong | Name of the studied model: MSSP ACO (3+ years); Provider involvement: Provider not involved (CMS regulates); Certainty of the Targets: Targets set; Nature of the incentivized entity: Group (organization); Motivation behind model: Cost savings & quality of care enhancement; Frequency of Incentives: Once a year; Nº Quality Indicators: Multiple separate indicators; Payment Channel: Coupled with usual reimbursement; | USA | Accountable care organizations | Not specified | - | Uptake of electronic health records; Consider repercussions on revenue of urban vs rural areas; | ACO participation imposes substantial costs on rural primary care clinics and lowers participation (might impede their formation); |
| 97 | Murphy, W. S.; Siddiqi, A.; Cheng, T.; Lin, B.; Terry, D.; Talmo, C. T.; Murphy, S. B. (2019). 2018 John Charnley Award: Analysis of US Hip Replacement Bundled Payments: Physician-initiated Episodes Outperform Hospital-initiated Episodes | Explanatory | Quantitative | Quantitative analysis based on CMS Limited Data Set for fee-for-service claims from the CMS | Strong | Name of the studied model: Bundled Payment for Care Improvement initiative (3+ years); Motivation behind model: Cost savings & quality of care enhancement; Type of Incentive: Both rewards and penalties; Payment Channel: Coupled with usual reimbursement | USA | Hospital networks and physician practices | Total hip arthoroplasty | Clinical & Cost outcomes (payments, 90-day mortality, readmissions); | Physician-initiated care episodes are less costly at baseline and achieve greater reductions in payments; Empower the operating surgeon who has the longest direct relationship with the patient to manage the episode of care; | - |
| 98 | Goldman, L. Elizabeth; Henderson, Stuart; Dohan, Daniel P.; Talavera, Jason A.; Dudley, R. Adams (2007). Public Reporting and Pay-for-Performance: Safety-Net Hospital Executives' Concerns and Policy Suggestions | Explanatory | Qualitative | Qualitative analysis based on executive interviews | Strong | Name of the studied model: P4P program (not specified further); Payment Channel: Decoupled from usual reiumbursement | USA | Safety-net hospitals | Not specified | Organization-related Outcomes/Experience (opinions of SNH executives towards measuring performance and the P4P focus); | - | Rural and other small urban hospitals reported feeling affected by few opportunities to marge or share resources; Risk adjustment using social factors has not been adequately evaluated for measures of quality (eg. patient satisfaction); |
| 99 | Truchil, A.; Dravid, N.; Singer, S.; Martinez, Z.; Kuruna, T.; Waulters, S. (2018). Lessons from the Camden Coalition of Healthcare Providers' First Medicaid Shared Savings Performance Evaluation | Explanatory | Quantitative | Case study, with quantitative analysis based on Camden Coalition’s ACO performance | Weak | Name of the studied model: Rutgers CSHP (2 years); Provider involvement: Provider not involved; Nature of the incentivized entity: Group (organization); Payment Channel: Coupled with usual reimbursement; Certainty of the Targets: Targets set; Frequency of Incentives: Once a year; | USA | Coalition of Healthcare Providers (community applied for Accountable care organization certification) | Not specified | Cost outcomes (shared savings); | - | Targets were a combination of new expensive therapies and increases in the unit cost of existing, commonly-prescribed therapies, rather than increases in disease prevalence or an uptick in utilization of these therapies; |
| 100 | Zhao, M.; Hamadi, H.; Haley, D. R.; Xu, J.; White-Williams, C.; Park, S. (2020). Telehealth: Advances in Alternative Payment Models | Explanatory | Quantitative | Quantitative analysis based on the American Hospital Association Annual Survey, Area Health Resource File, and Dartmouth Atlas Hospital Service Area dataset, all merged using Centers for Medicare and Medicaid Provider Number and County Federal Information Processing Standards | Strong | Name of the studied model: ACO model (not further specified) & Bundled payment model (3+ years); Provider involvement: Provider not involved; Nature of the incentivized entity: Group (organization); Payment Channel: Coupled with usual reimbursement; Certainty of the Targets: Targets set; Frequency of Incentives: Once a year; | USA | Hospitals | Not specified | - | Uptake of telehealth-related capabilities and their strong integration into care-delivery systems; Large system-affiliated, not-for-profit, and teaching hospitals have a greater propensity to adopt telehealth programs; | Clinical professionals are not adept to using telehealth-related technologies; |
| 101 | Karina, Newhall; David, Stone; Ryan, Svoboda; Philip, Goodney (2016). Possible consequences of regionally based bundled payments for diabetic amputations for safety net hospitals in Texas | Explanatory | Quantitative | Case study, with quantitative analysis based on publically available data from Centers for Medicare and Medicaid and the Texas Department of Health & Case Study | Moderate | Name of the studied model: Bundled Payments (not further specified); Type of Incentive: Both rewards and penalties; Payment Channel: Coupled with usual reimbursement; | USA | Safety net hospitals | Diabetes | - | - | A regionally based bundled payment model would lead to disproportionately larger financial losses at safety net hospitals; |
| 102 | Friedberg, M. W.; Chen, P. G.; Simmons, M.; Sherry, T.; Mendel, P.; Raaen, L.; Ryan, J.; Orr, P.; Vargo, C.; Carlasare, L.; Botts, C.; Blake, K. (2020). Effects of Health Care Payment Models on Physician Practice in the United States: Follow-Up Study | Descriptive | Qualitative | Case study, with qualitative analysis based on semistructured interviews with physician practice leaders, physicians, and other observers | Weak | Name of the studied model: Fee for service, capitation, episode-based and bundled, shared savings, pay for performance, retainer-based, and medical homes and accountable care organizations; Provider involvement: Provider not involved; Payment Channel: Coupled with usual reimbursement; Certainty of the Targets: Targets set; Frequency of Incentives: Once a year; | USA | Physician practices | Not specified | Organization-related Outcomes/Experience (challenges posed and strategies to deal with APMs, effects of models, physician practices' decisions to engage in the models); | Offer subsidies for up-front infrastructure investments and to partners that provided such infrastructure at nominal cost (eg.in exchange for a share of any bonuses received); | Internal financial incentives for individual physicians did not changed and remained modest. Individual physician financial incentives based on costs of care were almost nonexistent; High degree of financial risk aversion, which influenced their decisions to engage in new payment models; Practices of all sizes and specialties reported that understanding complex new payment models often entailed a significant resource investment, either to hire consultants or to build internal capabilities; Problems with data integrity and timeliness, errors in payment model execution, and incomprehensible incentives; Accelarating pace of change makes it hard for small primary care practices to find trustworthy advice (some practice leaders requested "time-outs" from further changes, to "catch-up"); |
| 103 | Friedberg, M. W.; Chen, P. G.; White, C.; Jung, O.; Raaen, L.; Hirshman, S.; Hoch, E.; Stevens, C.; Ginsburg, P. B.; Casalino, L. P.; Tutty, M.; Vargo, C.; Lipinski, L. (2015). Effects of Health Care Payment Models on Physician Practice in the United States | Descriptive | Qualitative | Case study, with qualitative analysis based on data from physician practices and other market participants and observers | Weak | Name of the studied model: Capitation, episode-based and bundled payment, shared savings, pay for performance, and retainer-based practice, accountable care organizations and medical homes; Provider involvement: Provider not involved; Payment Channel: Coupled with usual reimbursement; Certainty of the Targets: Targets set; Frequency of Incentives: Once a year; | USA | Physician practices | Not specified | Organization-related Outcomes/Experience (facilitators and barriers of models); | Rapid uptake and upgrading of electronic health records; Collaboration, with multispecialty teams working to prevent progression of disease; For some smaller, independent practices, merging with larger practices or hospitals was an attractive option for accessing the capital necessary; | Inconsistencies between financial and nonfinancial incentives; The multiplicity of PFP and other incentive programs has created a heavy administrative burden; Data on prices unavailable (limits practices' abilities to contain costs of care and causes frustration); Lack of linkage between physicians' incomes and quality/efficiency of care, and lack of alignment between when they thought they should do for patients and what they were paid to do (enhanced by changes in the payment models and regulations); Concerns from physicians and practice leaders about the complexity of the models; |
| 104 | Damberg, Cheryl L.; Elliott, Marc N.; Ewing, Brett A. (2015). Pay-For-Performance. Pay-For-Performance Schemes That Use Patient And Provider Categories Would Reduce Payment Disparities | Exploratory | Quantitative | Quantitative analysis based on data from the provider organizations that contracted with IHA health plans | Strong | Name of the studied model: Pay-for-performance program (no further specified); Provider involvement: Capitation rate is determined in negotiations between the plan and the provider organization; Motivation behind model: Financial; Nº Quality Indicators: Weighted measures; Performance measure: Absolute measures; Flexibility level: Fixed targets; Payment Channel: Coupled with usual reimbursement | USA | Provider organizations | Not specified | Cost outcomes (payments to disadvantaged provider organizations, payment differentials across provider organizations); | Post-adjustment by provider categories is an attractive potential addition to the design of incentive programs; Paying for improvement;  Creating stronger incentives among lower performers could better target the use of incentive dollars to drive improvements; | In the context of the California P4P program, provider organizations that served larger proportions of disadvantaged patients had lower average quality performance and lower average per member per month quality incentive payments in 2009, compared to provider organizations with smaller shares of disadvantaged patients; |
| 105 | George, M.; Bencic, S.; Bleiberg, S.; Alawa, N.; Sanghavi, D. (2014). Case study: Delivery and payment reform in congestive heart failure at two large academic centers | Explanatory | Qualitative | Qualitative analysis based on performance of two cases from Duke University Health System (“Duke”) and University of Colorado Hospital (“Colorado”) | Weak | Name of the studied model: Hospital Readmission Reduction Program & MSSP ACO (3+ years); Provider involvement: Provider not involved; Nature of Incentivized Entity: Distribute payments among all providers involved in the episode of care; Motivation behind model: Patient benefit & Financial interest; Type of Incentive: Both rewards and penalties; Nº Quality Indicators: In Hospital Readmission Reduction Program, 30-day readmission after discharge was reported Flexibility level: Fixed targets; Payment Channel: Coupled with usual reimbursement; Certainty of the Targets: Targets set; Frequency of Incentives: Once a year; | USA | Hospitals | Congestive heart failure | Clinical outcomes (preventable chronic heart failure readmission rates, quality of patient care transitions); | Putting in place a dedicated care team for each patient; Familiarity with the payment model and evidence of cost savings helped minimize the associated risks; Strong leadership; Improving communication, coordinating care across all providers and sites of care, and creating standard protocols for post-discharge processes (eg. follow-up procedures); | Financial incentives that are misaligned with the goals of improving patient care; Complex conditions lower predictability and standardization of care and costs; |
| 106 | Kim, D. H.; Lloyd, C.; Fernandez, D. K.; Spielman, A.; Bradshaw, D. (2017). A Direct Experience in a New Accountable Care Organization: Results, Challenges, and the Role of the Neurosurgeon | Explanatory | Quantitative | Case Studies, with quantitative analysis of an ACO (experiences, challenges, and successes, as well as to describe the role of the neurosurgery service) | Weak | Name of the studied model: MSSP ACO (3+ years); Provider involvement: Provider not involved (CMS regulates); Nature of incentivized entity: Payment formula adjusted to different staff members; Motivation behind model: Cost savings & quality of care enhancement; Type of Incentive: Not just Track 1, with both rewards and penalties, with rewards and penalties; Nº Quality Indicators: 33 quality measures and a composite quality score; Payment Channel: Coupled with usual reimbursement; Certainty of the Targets: Targets set; Frequency of Incentives: Once a year; | USA | Accountable care organizations | Cervical and lumbar surgery (conditions not specified) | Cost outcomes (total savings, cost per patient in cervical and lumbar intraoperative care); | Infrastructure investments and care coordination; Population health approach; Include neurosurgeons from early on (forming collaborative partnerships with the hospital); Establishing a cohesive, collaborative physician group that can make joint decisions, based on accurate data; | A movement toward tightly controlled and narrow networks will limit options available to a patient; |
| 107 | Bertone MP, Lagarde M, Witter S. Performance-based financing in the context of the complex remuneration of health workers: findings from a mixed-method study in rural Sierra Leone. BMC Health Serv Res. 2016;16: 286. | Exploratory | Qualitative | Qualitative analysis based on a survey, an 8-week longitudinal logbook collecting data, and in-depth interviews with workers | Moderate | Name of the studied model: Performance-Based Financing; Provider involvement: Provider not involved; Nature of the incentivized entity: Incentives cover facility running costs and individual staff incentives; Motivation behind model: Patient benefit & Financial interest; Type of Incentive: Payment of a financial bonus to healthcare providers based on their performance; Nº Quality Indicators: Multiple separate indicators; Performance measure: Absolute measures; Flexibility level: Fixed targets; Payment Channel: Partly coupled and partly decoupled from usual reimbursement; Certainty of the Targets: Targets set; Frequency of incentives: The performance bonus is calculated quarterly; | Sierra Leone | Community Health Centers, and community and maternal/child health posts | Not specified | Organization-related Outcomes/Experience (health workers' views on bonuses and salaries, implementation issues with the PBF scheme); | Examine financial incentives together and not independently; Clarification of responsibilities and tasks in service delivery; Perceived improvement in the physical working environment thanks to the facility component of the bonus; | Delays in the payment of the PBF bonus, due to lengthy verification procedures or other issues; |
| 108 | Bleser WK, Saunders RS, Muhlestein DB, Morrison SQ, Pham HH, McClellan MB. ACO quality over time: the MSSP experience and opportunities for system-wide improvement. Am J Accountable Care. 2018;6(1):e1–15. | Exploratory | Quantitative | Quantitative analysis based on secondary MSSP public use files linked to the Leavitt Partners ACO Database | Moderate | Name of the studied model: MSSP ACO (3+ years); Provider involvement: Provider not involved (CMS regulates); Nature of the incentivized entity: Group (organization); Motivation behind model: Cost savings & quality of care enhancement; Nº Quality Indicators: Several single measures and composite scores; Payment Channel: Coupled with usual reimbursement; Certainty of the Targets: Targets set; Frequency of Incentives: Once a year; | USA | Accountable care organizations | Not specified | Clinical & Cost outcomes (quality measures performance, quality of ACOs, PAC expenditures); | ACO infrastructure development funding, better relationships with PAC facilities, and opportunities for diverse ACOs to share their learnings; ACOs with 1 or more commercial contracts are documented as having higher quality than those with only public contracts; Hospital-led ACOs, with well-developed infrastructure and referral networks (requirement of on-site screening and specialists, to which smaller provider-led ACOs may not have direct access to); Provider-led ACOs often had higher patient/caregiver experience scores (more personal setting that patients may prefer); | Suboptimal startup cost mechanisms, including increased demand for resources to engage, attribute, and manage new beneficiaries; Smaller, physician-led ACOs reported less program experience, as well as risk bearing (fewer economies of scale and less ability to absorb startup costs); |
| 109 | Campbell, S.M., Reeves, D., Kontopantelis, E., Sibbald, B., Roland, M.: Effects of pay for performance on the quality of primary care in England. N. Engl. J. Med. 361(4), 368–378 (2009) | Explanatory | Quantitative | Quantitative analysis based on medical records and questionnaires | Weak | Name of the studied model: P4P (not further specified); Motivation behind model: Improve quality of care; Nº Quality Indicators: 136 indicators; Type & Size of payment: Payments make up approximately 25% of family practitioners’ income; Payment Channel: Decoupled from usual reiumbursement; Certainty of the Targets: Targets set; | UK | Family practices | Asthma, diabetes, and coronary heart disease | Clinical outcomes & Patient-reported Outcomes/Experience (quality of care for asthma, diabetes and heart disease, patients’ reports on access to care or on interpersonal aspects of care, level of continuity of care); | - | Practices focus on meeting rapid-access targets in which access to any doctor in the practice within 48 hours was linked to incentives but access to a particular physician was not; Hard to improve after achieving near-maximal scores and/or no schemes for improvement and/or little financial incentives for further improvement; |
| 110 | Cheng SH, Lee TT, Chen CC. A longitudinal examination of a pay-for- performance program for diabetes care: evidence from a natural experiment. Med Care 2012; 50(2): 109–116. | Explanatory | Quantitative | Quantitative analysis based on claims data for health care utilization | Strong | Name of the studied model: P4P (not further specified); Nature of incentivized entity: Incentives provided to health care providers; Motivation behind model: Improve quality of care; Type of Incentive: Only rewards; Nº Quality Indicators: Multiple separate indicators; Performance measure: Absolute measures; Payment Channel: Decoupled from usual reiumbursement; Certainty of the Targets: Targets set; | Taiwan | Nationwide national institute of health | Diabetes | Clinical & Cost outcomes (diabetes specific examinations and tests after enrollment, diabetes-related physician visits and hospitalizations, health care expenses); | Frequent and continuous financial incentives; Initial expenses of the P4P program during the first year might be offset by the potential savings in subsequent years for patients continually enrolled in the program (panel stability); | Potential underuse of otherwise needed services; |
| 111 | Chimhutu V, Lindkvist I, Lange S: When incentives work too well: locally implemented pay for performance(P4P) and adverse sanctions towards home birth in Tanzania-a qualitative study. BMC Health Serv Res 2014, 14:23. | Exploratory | Qualitative | Qualitative analysis based on in-depth interviews and focus groups | Strong | Name of the studied model: P4P (not further specified); Provider involvement: Provider not involved; Nature of incentivized entity: An increase in the utilization of health services can trigger a bonus to the responsible health workers/ managers; Motivation behind model: Improve quality of care; Type of Incentive: Only rewards; Nº Quality Indicators: Multiple separate indicators; Flexibility level: Fixed targets; Payment Channel: Decoupled from usual reiumbursement; Certainty of the Targets: Targets set; | Tanzania | Public dispensaries and health centers | Home birth | Organization-related Outcomes/Experience (health workers' expectations related to the introduction of P4P and knowledge about the P4P scheme); | Consider adverse effects when using external rewards for improved health outcomes; The extent to which bonus payments are perceived as fair, even in cases where they depend upon factors that are partly outside the control of the health workers; | A weak health infrastructure has been identified as one of the major threats to the effectiveness of P4P; |
| 112 | Colla CH, Lewis VA, Gottlieb DJ, Fisher ES. Cancer spending and accountable care organizations: evidence from the Physician Group Practice Demonstration. Healthc (Amst). 2013;1:100-107. | Explanatory | Quantitative | Quantitative analysis based on Medicare fee-for-service claims data | Moderate | Name of the studied model: Pioneer & MSSP ACO (3+ years); Provider involvement: Provider not involved (CMS regulates); Nature of the incentivized entity: Group (organization); Motivation behind model: Cost savings & quality of care enhancement; Type of Incentive: Both rewards and penalties; Payment Channel: Coupled with usual reimbursement; Certainty of the Targets: Targets set; Frequency of Incentives: Once a year; | USA | Accountable care organizations | Cancer | Clinical & Cost outcomes (Medicare spending on cancer care, in acute care payments for inpatient stays mortality among cancer patients, hospice use, hospital discharges, ICU days, and cancer-specific procedures or chemotherapy); | Focus on high-cost, high-risk groups such as cancer patients is important for success of the reforms | Changes in cancer treatment are difficult to implement and take a lot of time; |
| 113 | Colla CH, Lewis VA, Shortell SM, Fisher ES. First national survey of ACOs finds that physicians are playing strong leadership and ownership roles. Health Aff (Millwood). 2014; 33(6):964–971. [PubMed: 24889945] | Exploratory | Quantitative | Quantitative analysis based on the National Survey of Accountable Care Organizations | Moderate | Name of the studied model: Pioneer & MSSP ACO (3+ years); Provider involvement: Provider not involved (CMS regulates); Nature of the incentivized entity: Group (organization); Motivation behind model: Cost savings & quality of care enhancement; Type of Incentive: Both rewards and penalties; Payment Channel: Coupled with usual reimbursement; Certainty of the Targets: Targets set; Frequency of Incentives: Once a year; | USA | Accountable care organizations | Not specified | - | Strong, effective and active leadership from physicians; | The networks in physician-led ACOs may be limited because physicians are ill equipped to develop and manage relationships with multiple organizations; Difficult for physician-led ACOs to track medication compliance; |
| 114 | Dummit LA, Kahvecioglu D, Marrufo G, et al. Association between hospital participation in a Medicare bundled payment initiative and payments and quality outcomes for lower extremity joint replacement episodes. JAMA. 2016;316(12): 1267-1278. | Explanatory | Quantitative | Quantitative analysis based on Medicare Part A and Part B enrollment and claims data, and a mail survey | Strong | Name of the studied model: Bundled Payments for Care Improvement (3+ years); Nature of the incentivized entity: Group (organization); Motivation behind model: Patient benefit & Financial interest; Type of Incentive: When episode payments were below the target, participants were eligible for additional amounts; when payments were above the target, participants may have had to repay CMS; Performance measure: Absolute measures; Flexibility level: Fixed targets;; Payment Channel: Coupled with usual reimbursement; Certainty of the Targets: Targets set; | USA | Hospitals, physician group practices, postacute care providers, and home health agencies | Lower extremity joint (primarily hip and knee) replacement | Clinical & Cost outcomes (BPCI Medicare episode payments, 30-day unplanned readmissions, unplanned readmissions, 30-day emergency department visits, 90-day emergency department visits, 30-day postdischarge mortality, 90-day postdischarge mortality); | - | Bundled payment approaches might provide incentives to increase the number of episodes, particularly with less intensive patients, because of the opportunity for greater financial rewards; Bundled payment approaches might select patients who would be less costly to treat; |
| 115 | Ellimoottil C, Ryan AM, Hou H, Dupree J, Hallstrom B, Miller DC. Medicare’s new bundled payment for joint replacement may penalize hospitals that treat medically complex patients. Health Aff (Millwood). 2016;35:1651-1657. | Explanatory | Quantitative | Quantitative analysis based on Medicare claims | Moderate | Name of the studied model: Comprehensive Care for Joint Replacement bundled payment program (3+ years); Provider involvement: Provider not involved; Type of Incentive: Both rewards and penalties; Nº Quality Indicators: Multiple separate indicators; Performance measure: Absolute measures; Motivation behind model: Financial; Payment Channel: Coupled with usual reimbursement; Certainty of the Targets: Targets set; | USA | Hospitals, practices and clinics | Joint replacement | - | Using region-based target pricing led to reduced reconciliation payments to hospitals that treat medically complex patients; | - |
| 116 | Hollingsworth JM, Nallamothu BK, Yan P, et al. Medicare accountable care organizations are not associated with reductions in the use of low-value coronary revascularization. Circ Cardiovasc Qual Outcomes. 2018;11(6):e004492. | Explanatory | Quantitative | Quantitative analysis based on National Medicare claims from a 20% random sample of beneficiaries, and data from the Carrier, Denominator, Medicare Provider Analysis and Review, and Outpatient research identifiable files, | Moderate | Name of the studied model: Not specified; Nature of the incentivized entity: Group (organization); Payment Channel: Coupled with usual reimbursement; Certainty of the Targets: Targets set; | USA | Accountable care organizations | Coronary revascularization | Clinical outcomes (rates of low- and high- value coronary revascularization); | - | Incentives of MSSP and Pioneer may be too weak to limit the growth in spending on cardiac specialty care (too little skin in the game);  Lack of specialist involvement; |
| 117 | Hsu J, Price M, Vogeli C, et al. Bending The Spending Curve By Altering Care Delivery Patterns: The Role Of Care Management Within A Pioneer ACO. Health Aff (Project Hope). 2017;36(5):876-884. | Explanatory | Quantitative | Quantitative analysis based on Medicare claims data | Moderate | Name of the studied model: Pioneer ACO (3+ years); Provider involvement: Provider not involved (CMS regulates); Nature of the incentivized entity: Group (organization); Motivation behind model: Cost savings & quality of care enhancement; Type of Incentive: Both rewards and penalties; Payment Channel: Coupled with usual reimbursement; Certainty of the Targets: Targets set; Frequency of Incentives: Once a year; | USA | Accountable care organizations | Emergency care (conditions not specified) | Clinical & Cost outcomes (Medicare spending, rate of hospitalization, nonemergency ED visits); | Use policy solutions to reduce population turnover (eg. require beneficiaries to join ACOs); Targeting beneficiaries with high risks that their primary care physicians believe are modifiable; | It could take time and investment before even efficacious programs achieve clinical or financial payoffs; |
| 118 | Kalk A, Paul F, Grabosch E: ‘Paying for performance’ in Rwanda: does it pay off? Tropical Med Int Health 2010, 15(2):182–190. | Explanatory | Qualitative | Qualitative analysis based on a cross-sectoral literature review on P4P, and semi-structured interviews | Weak | Name of the studied model: P4P scheme (not specified further); Provider involvement: Provider not involved (CMS regulates); Payment Channel: Decoupled from usual reiumbursement; | Rwanda | Rwanda's health sector (not specified further) | Not specified | Organization-related Outcomes/experience (reported effects); | - | Overworked staff tend to neglect other core tasks for the sake of the incentives (consistently ignores other health threats and challenges); If the funding comes to an end, deep depression of staff motivation far below original levels might follow; |
| 119 | Lewis, V. A., Colla, C. H., Carluzzo, K. L., Kler, S. E. and Fisher, E. S., Accountable Care Organizations in the United States: market and demographic factors associated with formation. Health Serv Res, 2013. 48(6 Pt 1): p. 1840-58. | Explanatory | Quantitative | Quantitative analysis based on the American Community Survey, and Medicare fee-for-service claims data | Strong | Name of the studied model: Pioneer & MSSP ACO (3+ years); Provider involvement: Provider not involved (CMS regulates); Nature of the incentivized entity: Group (organization); Motivation behind model: Cost savings & quality of care enhancement; Type of Incentive: Both rewards and penalties; Payment Channel: Coupled with usual reimbursement; Certainty of the Targets: Targets set; Frequency of Incentives: Once a year; | USA | Hospitals | Not specified | Clinical & Cost outcomes (performance on quality, Medicare per capita spending, primary care physician groups, care penetration, poverty rates); | Financing programs such as Medicare’s Advanced Payment initiative, or modifications to risk adjustment methods; Tailor program to local context; High-cost and high-performing areas; | - |
| 120 | Lewis, V.A., Tierney, K.I., Colla, C.H. and Shortell, S.M. (2017), “The new frontier of strategic alliances in health care: new partnerships under accountable care organizations”, Social Science and Medicine, Vol. 190, pp. 1-10, available at: https://doi.org/10.1016/j.socscimed.2017.04.054 | Exploratory | Mixed Methods | Qualitative and quantitative analysis based on the National Survey of ACOs, Medicare ACO performance data and interviews with executive leaders of ACOs | Moderate | Name of the studied model: ACO model (not specified further) (3+ years); Provider involvement: Provider not involved (CMS regulates); Nature of the incentivized entity: Group (organization); Motivation behind model: Cost savings & quality of care enhancement; Type of Incentive: Both rewards and penalties; Payment Channel: Coupled with usual reimbursement; Certainty of the Targets: Targets set; Frequency of Incentives: Once a year; | USA | Accountable care organizations | Not specified | Organization-related Outcomes/experience (existing data and interviews with executive leaders on partnership ACOs) | Partnership selection is an important factor in forming a successful alliance (and conflict management);  Data sharing and transparency may help build stronger working relationships; | Competitive partners, rather than collaborative; |
| 121 | McDonald R, Roland M. Pay for performance in primary care in England and California: comparison of unintended consequences. Ann Fam Med 2009; 7(2): 121–127. | Exploratory | Qualitative | Qualitative analysis based on interviews with colleagues of researcher's informants | Moderate | Name of the studied model: UK - Quality and Outcomes Framework & USA - Pay-for-performance programs (not specified further); Nature of the incentivized entity: Payments to independent pratice associations or directly to practices; Motivation behind model: Improve quality of care; Type of Incentive: Only rewards; Nº Quality Indicators: Multiple separate indicators; Type & Size of payment: In England, payments contribute as much as 30% of practice income (reported lower in California); Payment Channel: Coupled with usual reimbursement; | UK & USA | Statewide primary care practices | Not specified | - | When designing incentive schemes, more attention needs to be paid to factors likely to produce unintended consequences; Payments based on data entered by physicians in the medical records (under the direct control of the physician); Reward systems that promote feelings of competence and autonomy (enhance motivation); | Programs involving surveillance and external rewards have the potential to damage intrinsic motivation (the desire to undertake a task for its own sake);  Many physicians were unaware of the target contents or had a poor understanding of the relation between their performance and incentives payments received; Loss of autonomy felt by physicians; Inability of physicians to exclude patients from quality calculations (some characteristics of the programs might actively disadvantage patients from underserved populations). The freedom to exclude might be abused if allowed; |
| 122 | McWilliams JM, Chernew ME, Landon BE, Schwartz AL. Performance differences in year 1 of pioneer accountable care organizations. N Engl J Med. 2015; 372(20):1927–1936. [PubMed: 25875195] | Descriptive | Quantitative | Quantitative analysis based on Medicare fee-for-service claims | Moderate | Name of the studied model: Pioneer ACO (3+ years); Provider involvement: Provider not involved (CMS regulates); Nature of the incentivized entity: Group (organization); Motivation behind model: Cost savings & quality of care enhancement; Type of Incentive: ACOs share in savings with Medicare if spending for an attributed patient population falls sufficiently below a financial benchmark and incur losses if spending sufficiently exceeds the benchmark; Nº Quality Indicators: 33 quality measures; Payment Channel: Coupled with usual reimbursement Certainty of the Targets: Targets set; Frequency of Incentives: Once a year; | USA | Accountable care organizations | Not specified | Cost outcomes (adjusted Medicare spending and per-beneficiary spending); | Larger specialty-oriented organizations have lower leakage (less beneficiaries seeking care outside the network) and may have greater incentives to invest in care management of assigned patients; Smaller primary care oriented organizations have higher contract penetration and may have greater incentives to make fundamental changes in capacity and care delivery that are organization-wide; | Limited influence of ACO contracts over the care provided by contracting organizations (low penetration) could discourage smaller ACOs from making important changes; Leakage (patients having care provided by specialists outside the assigned organizations); Unstably assigned beneficiaries were more likely than stably assigned beneficiaries to be in several high-cost groups that may be targeted for care management; |
| 123 | Muhlestein DB, Morrison SQ, Saunders RS, Bleser WK, McClellan MB, & Winfield LD (2018). Medicare Accountable Care Spending Patterns: Shifting Expenditures Associated with Savings. American Journal of Accountable Care, 6(1), 11–19. | Explanatory | Quantitative | Quantitative analysis based on MSSP public use files | Weak | Name of the studied model: MSSP ACO (3+ years); Nature of the incentivized entity: Group (organization); Motivation behind model: Cost savings & quality of care enhancement; Payment Channel: Coupled with usual reimbursement; Provider involvement: Provider not involved (CMS regulates); Certainty of the Targets: Targets set; Frequency of Incentives: Once a year; | USA | Accountable care organizations | Not specified | Cost outcomes (spending on inpatient, skilled nursing facility care and physician services); | ACOs may be able to achieve savings by re-distributing resources to different sites of care; | - |
| 124 | Nyweide DJ, Lee W, Cuerdon TT, et al. Association of Pioneer Accountable Care Organizations vs traditional Medicare fee for service with spending, utilization, and patient experience. JAMA. 2015;313(21):2152-2161. doi: 10.1001/jama.2015.4930. | Explanatory | Quantitative | Quantitative analysis based on claims data in the Chronic Conditions Ware­house | Strong | Name of the studied model: Pioneer ACO (3+ years); Provider involvement: Provider not involved (CMS regulates); Nature of the incentivized entity: Group (organization); Motivation behind model: Cost savings & quality of care enhancement; Type of Incentive: Both rewards and penalties; Nº Quality Indicators: 33 quality measures; Performance measure: Absolute measures; Payment Channel: Coupled with usual reimbursement**;** Certainty of the Targets: Targets set; Frequency of Incentives: Once a year; | USA | Accountable care organizations | Not specified | Clinical & Cost outcomes (total spending for beneficiaries, utilization of physician services, emergency department, and postacute care, scores for timely care and clinician communciation); | Learn how to effectively manage the care of a population of FFS Medicare beneficiaries to realize smaller increases in spending; | Hard to sustain such big focus on care management from year 1 to year 2; May take more time for some ACOs to rede­sign care delivery; High turnover of physicians and their aligned beneficiaries; Spillover effects, where ACOs serve beneficiaries not associated to those ACOs, making the comparison between ACOs and non-ACOs appear to have a smaller difference; Challenging for ACOs to continually im­prove on baseline performance over time, especially since growth in spending is declining; |
| 125 | Ogundeji YK, Jackson C, Sheldon T, Olubajo O, Ihebuzor N. Pay for performance in Nigeria: the influence of context and implementation on results. Health Policy Plann. 2016;31:955–63. | Exploratory | Qualitative | Qualitative analysis based on in-depth interviews with health workers | Moderate | Name of the studied model: Pay-for-performance program (no further specified); Nature of the incentivized entity: Group (organization) & Individual health workers have the opportunity to earn part of it as bonuses; Type of Incentive: Only rewards; Nº Quality Indicators: Multiple separate indicators; Performance measure: Absolute measures; Payment Channel: Decoupled from usual reiumbursement; Frequency of incentives: Quarterly payments to health facility and monthly payments to health workers; | Nigeria | Health facilities | Not specified | Organization-related Outcomes/experience (views and experiences of participants); | Adequate health worker understanding of the scheme and good managerial skills (improved motivation and performance); Minimising delays in incentive payments, effective communication and improving the health workers understanding of the P4P scheme; | Uncertainty of earning the incentives; Inadequate infrastructures (reduces motivation and overall results); |
| 126 | Ridde V, Yaogo M, Zongo S, Somé P-A, Turcotte-Tremblay A-M. Twelve months of implementation of health care performance-based financing in Burkina Faso: a qualitative multiple case study. Int J Health Plann Manage. 2018;33:e153–67. | Explanatory | Qualitative | Case study, with qualitative analysis based on empirical data collected from observations, informal and formal interviews, and documents | Moderate | Name of the studied model: Performance‐based financing interventions; Provider involvement: Provider not involved; Type of Incentive: Only rewards; Nº Quality Indicators: 23 indicators; Performance measure: Absolute measures; Payment Channel: Decoupled from usual reiumbursement; Certainty of the Targets: Targets set; | Burkina Faso | Regional hospital, district hospitals, and primary care health centres | Not specified | Organization-related Outcomes/experience (knowledge about PBF and its details); | - | Several elements of the local context and of the health system appear not to have been sufficiently considered (eg. presence of medical support personnel in health centres, and the essential role of COGES members); Front‐line workers do not know all the details of the intervention, and the central actors and funding agency are unable to control its functioning as a whole; Delays in paying bonuses; |
| 127 | Ssengooba F, McPake B, Palmer N. Why performance-based contracting failed in Uganda--an “open-box” evaluation of a complex health system intervention. Soc Sci Med. 2012;75:377–83. | Explanatory | Qualitative | Case study, with qualitative analysis based on collecting experiences of participants at district and hospital levels | Moderate | Name of the studied model: Performance-based contracting; Provider involvement: Provider not involved; Nature of incentivized entity: Members of the group were eligible to receive a monetary bonus if achieved/exceeded contract targets; Motivation behind model: Improve quality of care; Type of Incentive: Only rewards; Nº Quality Indicators: Multiple separate indicators; Performance measure: Absolute measures; Flexibility level: Absolute targets and improvements; Type & Size of payment: Maximum bonus was equivalent to 11% of the total annual grant amount provided by the government; Payment Channel: Coupled with usual reimbursement; Certainty of the Targets: Targets set; | Uganda | Hospitals | Not specified | Organization-related Outcomes/experience (problems with implementation of PBC); | - | Underfinancing of the initiative, underestimation of the technical, institutional capacity requirements for successful implementation, overloading of the implementation team, and the failure to consider important actors who influence outcomes; Inadequate time was allowed for the selection of service targets by the health centres (yet, they got ‘locked-in’ to these poor choices); Delays, short-cuts and uncertainty about the size and payment of bonuses; |
| 128 | Sutherland, S. E., Egan, B. M., Fleming, D. O., Helmrich, G. A., Davis, R. A., Rutledge, V., & Sinopoli, A. (2016). Medicare Shared Savings Program second-year results: Predictors of success. GHS Proceedings, 1(1), 22–27. | Explanatory | Quantitative | Quantitative analysis based on data from Centers for Medicare & Medicaid Services | Moderate | Name of the studied model: MSSP ACO (3+ years); Provider involvement: Provider not involved (CMS regulates); Nature of the incentivized entity: Group (organization); Motivation behind model: Cost savings & quality of care enhancement; Nº Quality Indicators: 33 quality measures; Payment Channel: Coupled with usual reimbursement; Certainty of the Targets: Targets set; Frequency of Incentives: Once a year; | USA | Accountable care organizations | Diabetes, mammography screening, and coronary artery disease | Clinical & Cost outcomes & Patient-reported Outcomes/Experience (savings, baseline costs, overall quality scores, hypertension/LDL cholesterol control in diabetes, mammography screening, patients’ rating of doctor, number of beneficiaries, health/functional status, and coronary artery disease composite score); | ACOs that generated savings had more years of experience in MSSP and higher overall quality scores; | - |
| 129 | Werner RM, Kolstad JT, Stuart EA, Polsky D. The effect of pay-for-performance in hospitals: lessons for quality improvement. Health Aff (Millwood) 2011; 30:690-8. | Exploratory | Quantitative | Quantitative analysis based on the Hospital Compare data available on the Centers for Medicare & Medicaid Services website, and supplemented with hospital characteristics from the Medicare Provider of Service File and Impact File | Moderate | Name of the studied model: Name of the studied model: Pay-for-performance program (no further specified); Provider involvement: Provider not involved (CMS regulates); Nature of the incentivized entity: Hospitals receive incentives; Motivation behind model: Improve quality of care; Type of Incentive: Financial bonuses were distributed to hospitals, and those performing bellow a threshold had to pay penalties for their low performance; Nº Quality Indicators: Separate measures, afterwards combined into composite scores; Performance measure: Absolute measures; Flexibility level: In addition to attaining bonuses, hospitals in the top 20 percent of improvement received another incentive; Payment Channel: Coupled with usual reimbursement; Certainty of the Targets: Targets set; Frequency of Incentives: Once a year; | USA | Hospitals | Acute care conditions (conditions not specified) | Clinical outcomes (performance of hospitals); | Providing up-front funding; Tailoring pay-for-performance programs to hospitals’ specific situations; Higher and more frequent bonuses;  Using financial penalties rather than only rewards may have a larger effect;  Tie incentives to a set of measures only until performance has improved;  Providing payments to individuals rather than organizations; | P4P is less effective with providers who lack the resources to invest in quality improvement; Very high rates of performance/Higher naseline before the program at participating hospitals, whose performance could not be improved much more; |
| 130 | Winblad U, Mor V, McHugh JP, & Rahman M (2017). ACO-Affiliated Hospitals Reduced Rehospitalizations From Skilled Nursing Facilities Faster Than Other Hospitals. Health Aff (Millwood), 36(1), 67–73. doi:10.1377/hlthaff.2016.0759 [PubMed: 28069848] | Explanatory | Quantitative | Quantitative analysis based on Centers for Medicare and Medicaid Services lists, Medicare Part A claims and Medicare enrollment files, and Minimum Data Sets | Strong | Name of the studied model: Pioneer & MSSP ACO (3+ years); Provider involvement: Provider not involved (CMS regulates); Nature of the incentivized entity: Group (organization); Motivation behind model: Cost savings & quality of care enhancement; Type of Incentive: Both rewards and penalties; Nº Quality Indicators: Multiple separate indicators; Payment Channel: Coupled with usual reimbursement; Certainty of the Targets: Targets set; Frequency of Incentives: Once a year; | USA | Hospitals | Elderly care (conditions not specified) | Clinical outcomes (rehospitalizations); | Use years of previous data to determine hospital performance; Enhancing information sharing and communication between hospitals and skilled nursing facilities; | - |
| 131 | Barbash IJ, Pike F, Gunn SR, Seymour CW, Kahn JM. Effects of physician-targeted pay for performance on use of spontaneous breathing trials in mechanically ventilated patients. Am J Respir Crit Care Med. 2017;196:56–63. https://doi.org/10.1164/rccm.201607-1505OC. | Explanatory | Quantitative | Quantitative analysis based on ICU-level data from an existing ICU patient registry derived from the UPMC electronic health record | Strong | Name of the studied model: P4P program (not further specified); Provider involvement: Provider not involved Nature of incentivized entity: Individual; Motivation behind model: Improve quality of care; Type of Incentive: Only rewards; Nº Quality Indicators: 3 measures; Performance measure: Absolute measures, divided between the three performance measures; Type & Size of payment: Annual quality bonus equal to approximately 7.5% of each ICU physician’s annual base salary Payment Channel: Coupled with usual reimbursement; Frequency of Incentives: Once a year; | USA | Hospitals | Mechanically ventilated patients (conditions not specified) | - | Selecting measures for which performance is low, rotating measures;  Ensure that all hospitals have an opportunity to improve; Continuously evaluate P4P programs to confirm that they are having their intended effect; | - |
| 132 | Chen H-J, Huang N, Chen L-S, Chou Y-J, Li C-P, Wu C-Y, et al. Does pay-for-performance program increase providers adherence to guidelines for managing hepatitis B and hepatitis C virus infection in Taiwan? PLoS One. 2016;11:e0161002. https://doi.org/10.1371/journal. pone.0161002. | Explanatory | Quantitative | Quantitative analysis based on population-based data extracted from the NHI claims files | Strong | Name of the studied model: The HBV/ HCV-P4P program (3+ years); Nature of the incentivized entity: Group (organization); Motivation behind model: Improve quality of care; Type of Incentive: Rewards providers for desirable performance, with possibility of bonuses based on using recommended services; Payment Channel: Decoupled from usual reiumbursement; Certainty of the Targets: Targets set; Frequency of Incentives: Once a year; | Taiwan | Hospitals and clinics | Hepatitis B and C | Clinical outcomes (enrollees’ attendance to twice-annual follow-up visits, receive recommended US examinations, and AST/ALT tests); | Quarterly reporting is clearly more expensive than annual, but the ongoing feedback is perceived to be valuable and the incremental cost is small; | Achievable bonus amount is too small, regardless of the frequency of payment; |
| 133 | Chen T-T. Hsueh Y-S (Arthur), Ko C-H, Shih L-N, Yang S-S. the effect of a hepatitis pay-for-performance program on outcomes of patients undergoing antiviral therapy. Eur J Pub Health. 2017;27:955–60. https://doi. org/10.1093/eurpub/ckx114. | Explanatory | Quantitative | Quantitative analysis based on databases of the National Health Insurance Administration | Strong | Name of the studied model: The hepatitis P4P program; Nature of the incentivized entity: Group (organization); Motivation behind model: Improve quality of care; Type of Incentive: Only rewards; Payment Channel: Decoupled from usual reiumbursement; | Taiwan | Hospitals | Hepatitis | Clinical outcomes (risk of hospital admission for severe hepatitis patients/developing liver cirrhosis); | - | Extra incentive to hire a dedicated case manager is too small; Observation period was not long enough (4years) with respect to the prognosis of the condition; Lack of a patient personalized care plan in the P4P program; |
| 134 | Chen, T.T., Chung, K.P., Lin, I.C., Lai, M.S.: The unintended consequence of diabetes mellitus pay-for-performance (P4P) program in Taiwan: are patients with more comorbidities or more severe conditions likely to be excluded from the P4P program? Health Serv. Res. (28 Sept 2010) | Explanatory | Quantitative | Quantitative analysis based on regular NHI claim data and the P4P database | Moderate | Name of the studied model: Diabetes Mellitus Pay-for-Performance Program; Provider involvement: Doctors are permitted to choose which of their DM patients are included in the P4P program; Nature of the incentivized entity: Group (organization); Type of Incentive: Only rewards; Nº Quality Indicators: Composite score; Performance measure: Incentive structure adopted a quality tournament, in which only the highest performing 25% of providers received rewards; Payment Channel: Decoupled from usual reiumbursement; | Taiwan | Hospitals | Diabetes mellitus | Organization-related Outcomes/experience (exclusion of patients from P4P programs); | - | The insufficient funds for implementation of the P4P program (eg. procedural changes such as the reporting of clinical data); Larger hospitals may be more likely to exclude patients from P4P programs; Patients with greater severity or comorbidity were more likely to be excluded from P4P programs; Hospitals with a lower baseline score in the previous year (2006) were more likely to exclude patients in the current year (2007), perhaps because hospitals with lower baseline scores in the previous year may want to increase their benefits in the next year; |
| 135 | Girault A, Bellanger M, Lalloué B, Loirat P, Moisdon JC, Minvielle E. Implementing hospital pay-for-performance: Lessons learned from the French pilot program. Health Policy 2017; 121: 407-417 [PMID: 28189271 DOI: 10.1016/j.healthpol.2017.01.007] | Explanatory | Mixed Methods | Quantitative and Qualitative analysis based on questionnaires and semi-structured interviews | Moderate | Name of the studied model: French IFAQ program (3 years); Performance measure: Awarding a financial bonus to hospitals depending on their relative rank, which was calculated based on quality indicator scores; Nature of the incentivized entity: Group (organization); Motivation behind model: Patient benefit & Financial interest; Type of Incentive: Only rewards; Nº Quality Indicators: Weighted average of none quality indicators; Type & Size of payment: Incentive was calculated as a portion of their annual budget, ranging from 0.3% to 0.5%; Payment Channel: Coupled with usual reimbursement; | France | Hospitals | Not specified | Organization-related Outcomes/experience (leaders' thoughts towards program awareness and strategies); | - | Lack of financial investment; Lack of awareness among the hospital staff, which breakes the rationale of “people react to financial incentives”; |
| 136 | Haarsager J, Krishnasamy R, Gray NA. Impact of pay for performance on access at first dialysis in Queensland. Nephrology. 2018;23:469–75. | Explanatory | Quantitative | Quantitative analysis based on patients registered with the Australia and New Zealand Dialysis and Transplant Registry | Strong | Name of the studied model: Queensland’s pay-for-performance program; Provider involvement: Provider not involved; Nature of the incentivized entity: Payments made to renal units; Motivation behind model: Improve quality of care; Type of Incentive: Only rewards; Nº Quality Indicators: Multiple separate indicators; Payment Channel: Decoupled from usual reiumbursement; Certainty of the Targets: Targets set; Frequency of Incentives: Payments to individual renal units were made bi-annually; | Australia | Clinical practices | Peritoneal dialysis, and haemodialysis | Clinical outcomes (change in AVF/AVG rate at first haemodialysis or PD); | Pay for performance has been shown to be more effective when incentive funds are explicit and direct | Improvements in planning take time to have an impact; Staff did not receive the payment for a prolonged period after the decision to create dialysis access; |
| 137 | Hsieh H-M, Lin T-H, Lee I-C, Huang C-J, Shin S-J, Chiu H-C. The association between participation in a pay-for-performance program and macrovascular complications in patients with type 2 diabetes in Taiwan: a nationwide population-based cohort study. Prev Med. 2016;85:53–9. https://doi.org/10. 1016/j.ypmed.2015.12.013. | Explanatory | Quantitative | Quantitative analysis based on nationwide diabetes P4P database and NHI administrative claims database | Strong | Name of the studied model: P4P (not further specified); Nature of incentivized entity: Individual; Motivation behind model: Improve quality of care; Type of Incentive: Only rewards; Nº Quality Indicators: Composite score; Performance measure: Both absolute and relative to others; Payment Channel: Decoupled from usual reiumbursement; | Taiwan | Hospitals and clinics | Type II diabetes | Clinical outcomes (risk of macrovacular complication); | Adherence to guidelines (eg. treatment recommendations for diabetes and for common diabetes-related diseases, like hypertension); Multidisciplinary team approach to the P4P program (team included: physicians, nurses, case manager, pharmacists, nutritionists and information technologists; | - |
| 138 | Jan CF, Lee MC, Chiu CM, Huang CK, Hwang SJ, Chang CJ, Chiu TY. Awareness of, attitude toward, and willingness to participate in pay for performance programs among family physicians: a cross-sectional study. BMC Fam Pract 2020; 21: 60 [PMID: 32228473 DOI: 10.1186/s12875-020-01118-9] | Exploratory | Quantitative | Quantitative analysis based on a structured questionnaire | Weak | Name of the studied model: P4P (not further specified); Motivation behind model: Improve health outcomes; Type of Incentive: Only rewards; Payment Channel: Decoupled from usual reiumbursement | Taiwan | Family practices | Not specified | Organization-related Outcomes/experience (physicians' awareness/attitude of P4P programs); | Better awareness and understanding of P4P programs, and a less negative attitude toward P4P programs (increase motivation); Providing technical and educational support, reducing administrative burden, forging a cooperative relationship with other medical facilities or healthcare providers, developing more accurate quality measures, and minimizing unintended consequences; | - |
| 139 | Kuo RNC, Chung KP, Lai MS. Effect of the Pay-for-Performance Program for Breast Cancer Care in Taiwan. J Oncol Pract. 2011;7(3S):e8s–15s. | Explanatory | Quantitative | Quantitative analysis based on Taiwan Cancer Database and National Health Insurance Database | Moderate | Name of the studied model: Pay-for- performance program for breast cancer care; Motivation behind model: Improve quality of care; Type of Incentive: Only rewards; Nº Quality Indicators: Multiple separate indicators; Payment Channel: Decoupled from usual reiumbursement; Certainty of the Targets: Targets set; | Taiwan | Hospitals | Breast cancer | Clinical outcomes (quality care of enrollees); | Include scaled bonuses and incentivize to achieve and maintain performance at the target level; Financial incentives for improvement and for completing planned treatments; | Hospitals that are not well coordinated prefer to retain the original payment scheme so as to reduce financial risk in the case of incomplete treatment or overuse of resources because of complications; The design of financial incentives within the BC-P4P program may be cause for some concern; BC-P4P program may reward hospitals for performance even though they had already performed better before joining the BC-P4P program; |
| 140 | Marsteller JA, Young JH, Fakeye OA, et al. Early provider perspectives within an accountable care organization. Am J Accountable Care 2016:4:27–37. | Exploratory | Quantitative | Quantitative analysis based on an online provider survey | Weak | Name of the studied model: MSSP ACO (3+ years); Provider involvement: Provider not involved (CMS regulates); Nature of the incentivized entity: Group (organization); Motivation behind model: Cost savings & quality of care enhancement; Nº Quality Indicators: Multiple separate indicators; Payment Channel: Coupled with usual reimbursement; Certainty of the Targets: Targets set; Frequency of Incentives: Once a year; | USA | Accountable care organizations | Not specified | Organization-related Outcomes/Experience (providers' rating of comfort, understanding, expectations about becoming an ACO, satisfaction with chronic care, reported coordination/communication, use of care coordinators, behavioral therapists or support staff); | Incorporating quality of care into criteria for promotion, and rewarding clinical excellence; Effective engagement of providers (motivate the care transformations necessary to improve outcomes), by involving them in shared savings distribution schemes,  Improve communication and coordination of care over enhancements (cooperation among physicians and acute care facilities relies on alignment of incentives across settings); | Difficulty accessing Electronic Health Records information on encounters outside of the ACO network; Care coordination challenges within the ACO and inadequate time during office visits for proper attention to beneficiaries with complex conditions; |
| 141 | McConnell KJ, Renfro S, Chan BK, et al. Early Performance in Medicaid Accountable Care Organizations: A Comparison of Oregon and Colorado. JAMA Intern Med. 2017;177(4):538-545. | Explanatory | Quantitative | Quantitative analysis based on data from each state’s Medicaid agency | Strong | Name of the studied model: Medicaid ACO model (not further specified) (3+ years); Provider involvement: Provider not involved (CMS regulates); Nature of the incentivized entity: Group (organization); Motivation behind model: Patient benefit & Financial interest; Type of Incentive: Both rewards and penalties; Payment Channel: Coupled with usual reimbursement; Certainty of the Targets: Targets set; Frequency of Incentives: Once a year; | USA | Coordinated care organizations and regional care collaborative organizations | Not specified | Clinical & Cost outcomes (standardized expenditures, emergency department visits, primary care visits, acute preventable hospital admissions, measures of access/appropriateness of care); | Focus on manageable, incremental steps has been followed by growth in enrollment, reductions in utilization, and improvement in some key performance indicators; | Organizations may need more time to fully implement changes that translate to greater savings; There may be limits to the extent to which relative savings can be achieved in a period of shrinking (as opposed to growing) health care spending; |
| 142 | Roberts ET, Zaslavsky AM, McWilliams JM. The Value-Based Payment Modifier: Program Outcomes and Implications for Disparities. Ann Intern Med. 2018 Feb 20;168(4):255-65. | Explanatory | Quantitative | Quantitative analysis based on claims and enrollment data for a random 20% sample of beneficiaries continuously enrolled in Part A and B of fee-for-service Medicare | Strong | Name of the studied model: Value-Based Payment Modifier; Provider involvement: Provider not involved (CMS regulates); Nature of the incentivized entity: Group (organization); Motivation behind model: Patient benefit & Financial interest; Type of Incentive: Both rewards and penalties; Nº Quality Indicators: Multiple separate indicators; Frequency of Incentives: Once a year; | USA | Hospitals | Not specified | Clinical & Cost outcomes (performance on quality and spending measures); | Incentives to improve quality and lower spending; Depleting providers’ resources to improve care for vulnerable patients, and use penalties to create incentives for practices to avoid the sicker or poorer patients (risk adjustment); Control over selection of quality measures; | Although bonuses were much larger than penalties, practices had to perform at least one standard deviation better than the mean to be eligible for a bonus, which may have weakened incentives for poor performers to improve; P4P programs with weak incentives and inadequate risk adjustment could contribute to health care disparities without eliciting a behavioral change that improves care on average; Some practices may have been unaware of the program, and others may have needed more than two years to respond effectively to the incentives even if they found them sufficiently strong to warrant a response; |
| 143 | Whitcomb WF, Lagu T, Krushell RJ, Lehman AP, Greenbaum J, McGirr J, et al. Experience with designing and implementing a bundled payment program for total hip replacement. Jt Comm J Qual Patient Saf 2015;41:406-13. | Descriptive | Quantitative | Quantitative analysis based on data from primary stakeholders, including New England Orthopedic Surgeons and affiliates of Baystate Health | Weak | Name of the studied model: Bundled payment program; Provider involvement: Provider not involved; Motivation behind model: Patient benefit & Financial interest; Type of Incentive: Only rewards; Payment Channel: Coupled with usual reimbursement; | USA | Baystate Health, an integrated health care delivery system in western Massachusetts | Total hip replacement | Clinical & Cost outcomes (length of hospital stay, discharge to home or home with services, total payments, and posthospital payments); | Diverse committee structure (using both a clinical and finance team), and engaging stakeholders early on; Commitment of the physician group, the willingness of the health plan representatives to provide financial and clinical input, and the dedication of hospitals and associations; | Pilot program encountered substantial administrative burden related to the manual administration of claims; Significant regulatory delays to contract approval and no risk-adjustment or stop-loss features; |

**References (Included Articles)**

1. Mandel, K. E.; Kotagal, U. R. (2007). Pay for Performance Alone Cannot Drive Quality
2. Shetty, V. A.; Balzer, L. B.; Geissler, K. H.; Chin, D. L. (2019). Association Between Specialist Office Visits and Health Expenditures in Accountable Care Organizations
3. Kaufman, B. G.; O'Brien, E. C.; Stearns, S. C.; Matsouaka, R.; Holmes, G. M.; Weinberger, M.; Song, P. H.; Schwamm, L. H.; Smith, E. E.; Fonarow, G. C.; Xian, Y. (2019). The Medicare Shared Savings Program and Outcomes for Ischemic Stroke Patients: a Retrospective Cohort Study
4. Thomas, M. E. (2008). The providers' coordination of care: a model for collaboration across the continuum of care
5. Chimhutu, V.; Songstad, N. G.; Tjomsland, M.; Mrisho, M.; Moland, K. M. (2016). The inescapable question of fairness in Pay-for-performance bonus distribution: a qualitative study of health workers' experiences in Tanzania
6. Paul, David P. (2014). The PGP Demonstrations: Were They Sufficient to Justify Accountable Care Organizations?...Physician Group Practice
7. Harvey, Jillian B.; Vanderbrink, Jocelyn; Mahmud, Yasmin; Kitt‐Lewis, Erin; Wolf, Laura; Shaw, Bethany; Ridgely, M. Susan; Damberg, Cheryl L.; Scanlon, Dennis P.; Kitt-Lewis, Erin (2020). Understanding how health systems facilitate primary care redesign
8. Parasrampuria, S.; Oakes, A. H.; Wu, S. S.; Parikh, M. A.; Padula, W. V. (2018). VALUE AND PERFORMANCE OF ACCOUNTABLE CARE ORGANIZATIONS: A COST-MINIMIZATION ANALYSIS
9. Resnick, M. J.; Graves, A. J.; Gambrel, R. J.; Thapa, S.; Buntin, M. B.; Penson, D. F. (2018). The association between Medicare accountable care organization enrollment and breast, colorectal, and prostate cancer screening
10. Hearld, L. R.; Carroll, N.; Hall, A. (2019). The adoption and spread of hospital care coordination activities under value-based programs
11. Duggal, R.; Zhang, Y.; Diana, M. L. (2018). The Association Between Hospital ACO Participation and Readmission Rates
12. Pittman, P.; Forrest, E. (2015). The changing roles of registered nurses in Pioneer Accountable Care Organizations
13. Diana, Mark L.; Yongkang, Zhang; Yeager, Valerie A.; Stoecker, Charles; Counts, Catherine R. (2019). The impact of accountable care organization participation on hospital patient experience
14. Ouayogodé, M. H.; Mainor, A. J.; Meara, E.; Bynum, J. P. W.; Colla, C. H. (2019). Association Between Care Management and Outcomes Among Patients With Complex Needs in Medicare Accountable Care Organizations
15. Schur, Claudia L.; Sutton, Janet P. (2017). Physicians In Medicare ACOs Offer Mixed Views Of Model For Health Care Cost And Quality
16. Nyweide, D. J.; Lee, W.; Colla, C. H. (2020). Accountable Care Organizations' Increase In Nonphysician Practitioners May Signal Shift For Health Care Workforce
17. Lin, Yi-Ling; Ortiz, Judith; Boor, Celeste (2018). ACOs' impact on hospitalization rates of rural older adults with diabetes: Early indications
18. Borza, T.; Oerline, M. K.; Skolarus, T. A.; Norton, E. C.; Dimick, J. B.; Jacobs, B. L.; Herrel, L. A.; Ellimoottil, C.; Hollingsworth, J. M.; Ryan, A. M.; Miller, D. C.; Shahinian, V. B.; Hollenbeck, B. K. (2019). Association Between Hospital Participation in Medicare Shared Savings Program Accountable Care Organizations and Readmission Following Major Surgery
19. Colla, Carrie H.; Lewis, Valerie A.; Kao, Lee-Sien; O'Malley, A. James; Chang, Chiang-Hua; Fisher, Elliott S. (2016). Association Between Medicare Accountable Care Organization Implementation and Spending Among Clinically Vulnerable Beneficiaries
20. Lewis, Valerie A.; McClurg, Asha Belle; Smith, Jeremy; Fisher, Elliott S.; Bynum, Julie P. W. (2013). Attributing Patients To Accountable Care Organizations: Performance Year Approach Aligns Stakeholders' Interests
21. McWilliams, J. Michael (2016). Changes in Medicare Shared Savings Program Savings From 2013 to 2014
22. Song, Z.; Safran, D. G.; Landon, B. E.; Landrum, M. B.; He, Y.; Mechanic, R. E.; Day, M. P.; Chernew, M. E. (2012). The 'Alternative Quality Contract,' based on a global budget, lowered medical spending and improved quality
23. Gilstrap, L. G.; Huskamp, H. A.; Stevenson, D. G.; Chernew, M. E.; Grabowski, D. C.; McWilliams, J. M. (2018). Changes In End-Of-Life Care In The Medicare Shared Savings Program
24. Schulz, J.; DeCamp, M.; Berkowitz, S. A. (2015). Medicare Shared Savings Program: public reporting and shared savings distributions
25. Urwin, J. W.; Caldarella, K. L.; Matloubieh, S. E.; Lee, E.; Mugiishi, M.; Kohatsu, L.; Yoshimoto, J.; Tom, J.; Okamura, S.; Wang, E.; Zhu, J.; Emanuel, E. J.; Volpp, K. G.; Navathe, A. S. (2020). Designing a commercial medical bundle for cancer care: Hawaii Medical Service Association's Cancer Episode Model
26. Saint-Lary, O.; Leroux, C.; Dubourdieu, C.; Fournier, C.; François-Purssell, I. (2015). Patients' views on pay for performance in France: a qualitative study in primary care
27. Lam, M. B.; Zheng, J.; Orav, E. J.; Jha, A. K. (2019). Early Accountable Care Organization Results in End-of-Life Spending Among Cancer Patients
28. McWilliams, J. M.; Hatfield, L. A.; Chernew, M. E.; Landon, B. E.; Schwartz, A. L. (2016). Early Performance of Accountable Care Organizations in Medicare
29. Sutton, M.; Nikolova, S.; Boaden, R.; Lester, H.; McDonald, R.; Roland, M.; Sutton, Matt; Nikolova, Silviya; Boaden, Ruth; Lester, Helen; McDonald, Ruth; Roland, Martin (2012). Reduced mortality with hospital pay for performance in England
30. McWilliams, J. M.; Hatfield, L. A.; Landon, B. E.; Hamed, P.; Chernew, M. E. (2018). Medicare Spending after 3 Years of the Medicare Shared Savings Program
31. Schulz, J.; DeCamp, M.; Berkowitz, A. S. A. (2018). Spending Patterns Among Medicare ACOs That Have Reduced Costs
32. Rose, S.; Zaslavsky, A. M.; McWilliams, J. M. (2016). Variation In Accountable Care Organization Spending And Sensitivity To Risk Adjustment: Implications For Benchmarking
33. Lam, M. B.; Figueroa, J. F.; Zheng, J.; Orav, E. J.; Jha, A. K. (2018). Spending Among Patients With Cancer in the First 2 Years of Accountable Care Organization Participation
34. Bleser, W. K.; Saunders, R. S.; Muhlestein, D. B.; McClellan, M. (2019). Why Do Accountable Care Organizations Leave The Medicare Shared Savings Program?
35. Schulz, J.; DeCamp, M.; Berkowitz, S. A. (2017). Regional cost and experience, not size or hospital inclusion, helps predict ACO success
36. Lewis, Valerie A.; Colla, Carrie H.; Schpero, William L.; Shortell, Stephen M.; Fisher, Elliott S. (2014). POLICY. ACO Contracting With Private and Public Payers: A Baseline Comparative Analysis
37. Kim, H.; Keating, N. L.; Perloff, J. N.; Hodgkin, D.; Liu, X.; Bishop, C. E. (2019). Aggressive Care near the End of Life for Cancer Patients in Medicare Accountable Care Organizations
38. Jones, M.; Hsu, C.; Pearson, D.; Wolford, D.; Labby, D. (2011). An alternative to pay-for-performance: one health plan's approach to quality improvement
39. Benchetrit, L.; Zimmerman, C.; Bao, H.; Dharmarajan, K.; Altaf, F.; Herrin, J.; Lin, Z.; Krumholz, H. M.; Drye, E. E.; Lipska, K. J.; Spatz, E. S. (2019). Admission diagnoses among patients with heart failure: Variation by ACO performance on a measure of risk-standardized acute admission rates
40. Eddy, D. M.; Shah, R. (2012). A simulation shows limited savings from meeting quality targets under the Medicare Shared Savings Program
41. Kaufman, B. G.; Anderson, D.; Bleser, W. K.; Muhlestein, D. B.; Smith, N.; Clough, J.; McClellan, M. B.; Saunders, R. (2021). Association of ACO Shared Savings Success and Serious Illness Spending
42. Cole, E. S.; Leighton, C.; Zhang, Y. (2018). Distribution of Visits for Chronic Conditions Between Primary Care and Specialist Providers in Medicare Shared Savings Accountable Care Organizations
43. Eriksson, T.; Tropp, H.; Wiréhn, A. B.; Levin, LÅ (2020). A pain relieving reimbursement program? Effects of a value-based reimbursement program on patient reported outcome measures
44. Modi, P. K.; Kaufman, S. R.; Borza, T.; Yan, P.; Miller, D. C.; Skolarus, T. A.; Hollingsworth, J. M.; Norton, E. C.; Shahinian, V. B.; Hollenbeck, B. K. (2018). Variation in prostate cancer treatment and spending among Medicare shared savings program accountable care organizations
45. Barnett, M. L.; McWilliams, J. M. (2018). Changes in specialty care use and leakage in Medicare accountable care organizations
46. Ouayogodé, M. H.; Colla, C. H.; Lewis, V. A. (2017). Determinants of success in Shared Savings Programs: An analysis of ACO and market characteristics
47. Murray, Genevra F.; D'Aunno, Thomas; Lewis, Valerie A. (2021). Critical issues in alliances between management partners and accountable care organizations...AcademyHealth 2018 Annual Research Meeting in Seattle, Washington
48. Lewis, V. A.; Schoenherr, K.; Fraze, T.; Cunningham, A. (2019). Clinical coordination in accountable care organizations: A qualitative study
49. Lewis, V. A.; Tierney, K. I.; Fraze, T.; Murray, G. F. (2019). Care Transformation Strategies and Approaches of Accountable Care Organizations
50. Sukul, D.; Ryan, A. M.; Yan, P.; Markovitz, A.; Nallamothu, B. K.; Lewis, V. A.; Hollingsworth, J. M. (2019). Cardiologist Participation in Accountable Care Organizations and Changes in Spending and Quality for Medicare Patients With Cardiovascular Disease
51. Glickman, Seth W.; Boulding, William; Roos, Jason M. T.; Staelin, Richard; Peterson, Eric D.; Schulman, Kevin A. (2009). Alternative pay-for-Performance Scoring Methods: Implications for Quality Improvement and Patient Outcomes
52. Kennedy, G.; Lewis, V. A.; Kundu, S.; Mousqués, J.; Colla, C. H. (2020). Accountable Care Organizations and Post-Acute Care: A Focus on Preferred SNF Networks
53. Saleh, Shadi S.; Alameddine, Mohamad S.; Natafgi, Nabil M. (2013). ACCEPTABILITY OF QUALITY REPORTING AND PAY FOR PERFORMANCE AMONG PRIMARY HEALTH CENTERS IN LEBANON
54. Bazzoli, G. J.; Harless, D. W.; Chukmaitov, A. S. (2019). A taxonomy of hospitals participating in Medicare accountable care organizations
55. Gu, J.; Huckfeldt, P.; Sood, N. (2021). The Effects of Accountable Care Organizations Forming Preferred Skilled Nursing Facility Networks on Market Share, Patient Composition, and Outcomes
56. McWilliams, J. M.; Gilstrap, L. G.; Stevenson, D. G.; Chernew, M. E.; Huskamp, H. A.; Grabowski, D. C. (2017). Changes in Postacute Care in the Medicare Shared Savings Program
57. Manongi, R.; Mushi, D.; Kessy, J.; Salome, S.; Njau, B. (2014). Does training on performance based financing make a difference in performance and quality of health care delivery? Health care provider's perspective in Rungwe Tanzania
58. Borza, T.; Kaufman, S. R.; Yan, P.; Herrel, L. A.; Luckenbaugh, A. N.; Miller, D. C.; Skolarus, T. A.; Jacobs, B. L.; Hollingsworth, J. M.; Norton, E. C.; Shahinian, V. B.; Hollenbeck, B. K. (2018). Early effect of Medicare Shared Savings Program accountable care organization participation on prostate cancer care
59. Lin, M. P.; Revette, A.; Carr, B. G.; Richardson, L. D.; Wiler, J. L.; Schuur, J. D. (2020). Effect of Accountable Care Organizations on Emergency Medicine Payment and Care Redesign: A Qualitative Study
60. Constantinou, Panayotis; Sicsic, Jonathan; Franc, Carine (2017). Effect of pay-for-performance on cervical cancer screening participation in France
61. Friedberg, M. W.; Rosenthal, M. B.; Werner, R. M.; Volpp, K. G.; Schneider, E. C. (2015). Effects of a Medical Home and Shared Savings Intervention on Quality and Utilization of Care
62. Ouayogodé, M. H.; Meara, E.; Ho, K.; Snyder, C. M.; Colla, C. H. (2021). Estimates of ACO savings in the presence of provider and beneficiary selection
63. D'Aunno, T.; Broffman, L.; Sparer, M.; Kumar, S. R. (2018). Factors That Distinguish High-Performing Accountable Care Organizations in the Medicare Shared Savings Program
64. Karim, S. A.; Nevola, A.; Morris, M. E.; Tilford, J. M.; Chen, H. F. (2021). Financial Performance of Hospitals in the Appalachian Region Under the Hospital Readmissions Reduction Program and Hospital Value-Based Purchasing Program
65. Ouayogodé, M. H.; Meara, E.; Chang, C. H.; Raymond, S. R.; Bynum, J. P. W.; Lewis, V. A.; Colla, C. H. (2018). Forgotten patients: ACO attribution omits those with low service use and the dying
66. Kim, Y.; Thirukumaran, C. P.; Li, Y. (2018). Greater Reductions in Readmission Rates Achieved by Urban Hospitals Participating in the Medicare Shared Savings Program
67. Carroll, N. W.; Clement, J. P. (2020). Hospital Performance in the First 6 Years of Medicare's Value-Based Purchasing Program
68. Blustein, J.; Borden, W. B.; Valentine, M. (2010). Hospital performance, the local economy, and the local workforce: findings from a US National Longitudinal Study
69. Tory, H. Hogan; Christy Harris, Lemak; Nataliya, Ivankova; Larry, R. Hearld; Jack, Wheeler; Nir, Menachemi (2018). Hospital Vertical Integration Into Subacute Care as a Strategic Response to Value-Based Payment Incentives, Market Factors, and Organizational Factors: A Multiple-Case Study
70. Chien, A. T.; Schiavoni, K. H.; Sprecher, E.; Landon, B. E.; McNeil, B. J.; Chernew, M. E.; Schuster, M. A. (2016). How Accountable Care Organizations Responded to Pediatric Incentives in the Alternative Quality Contract
71. Coulibaly, A.; Gautier, L.; Zitti, T.; Ridde, V. (2020). Implementing performance-based financing in peripheral health centres in Mali: what can we learn from it?
72. Hayen, Arthur; van den Berg, Michael Jack; Struijs, Jeroen Nathan; Westert, Gerard Pieter (2021). Dutch shared savings program targeted at primary care: Reduced expenditures in its first year
73. Larson, B. K.; Van Citters, A. D.; Kreindler, S. A.; Carluzzo, K. L.; Gbemudu, J. N.; Wu, F. M.; Nelson, E. C.; Shortell, S. M.; Fisher, E. S. (2012). Insights from transformations under way at four Brookings-Dartmouth accountable care organization pilot sites
74. Cornell, Tatiana (2020). Leadership skills essential in the value-based care era
75. Cassandra, Leighton; Evan, Cole; A. Everette James; Julia, Driessen (2019). Medicare Shared Savings Program ACO network comprehensiveness and patient panel stability
76. Markovitz, A. A.; Rozier, M. D.; Ryan, A. M.; Goold, S. D.; Ayanian, J. Z.; Norton, E. C.; Peterson, T. A.; Hollingsworth, J. M. (2020). Low-Value Care and Clinician Engagement in a Large Medicare Shared Savings Program ACO: a Survey of Frontline Clinicians
77. Modi, P. K.; Kaufman, S. R.; Caram, M. E.; Ryan, A. M.; Shahinian, V. B.; Hollenbeck, B. K. (2021). Medicare Accountable Care Organizations and the Adoption of New Surgical Technology
78. Huang, N.; Raji, M.; Lin, Y. L.; Chou, L. N.; Kuo, Y. F. (2021). Nurse Practitioner Involvement in Medicare Accountable Care Organizations: Association With Quality of Care
79. Zhu, X.; Mueller, K.; Huang, H.; Ullrich, F.; Vaughn, T.; MacKinney, A. C. (2019). Organizational Attributes Associated With Medicare ACO Quality Performance
80. Zhang, H.; Wernz, C.; Hughes, D. R. (2018). Modeling and designing health care payment innovations for medical imaging
81. Olafsdottir, A. E.; Mayumana, I.; Mashasi, I.; Njau, I.; Mamdani, M.; Patouillard, E.; Binyaruka, P.; Abdulla, S.; Borghi, J. (2014). Pay for performance: an analysis of the context of implementation in a pilot project in Tanzania
82. Aditi, P. Sen; Lena, M. Chen; Lok Wong, Samson; Arnold, M. Epstein; Karen, E. Joynt Maddox (2018). Performance in the Medicare Shared Savings Program by Accountable Care Organizations Disproportionately Serving Dual and Disabled Populations
83. Han, M. A.; Clarke, R.; Ettner, S. L.; Steers, W. N.; Leng, M.; Mangione, C. M. (2016). Predictors of Out-of-ACO Care in the Medicare Shared Savings Program
84. Chukmaitov, A.; Harless, D. W.; Bazzoli, G. J.; Muhlestein, D. B. (2019). Preventable Hospital Admissions and 30-Day All-Cause Readmissions: Does Hospital Participation in Accountable Care Organizations Improve Quality of Care?
85. Albright, B. B.; Lewis, V. A.; Ross, J. S.; Colla, C. H. (2016). Preventive Care Quality of Medicare Accountable Care Organizations: Associations of Organizational Characteristics With Performance
86. Herrel, L. A.; Ayanian, J. Z.; Hawken, S. R.; Miller, D. C. (2017). Primary care focus and utilization in the Medicare shared savings program accountable care organizations
87. Fraze, T. K.; Lewis, V. A.; Tierney, E.; Colla, C. H. (2018). Quality of Care Improves for Patients with Diabetes in Medicare Shared Savings Accountable Care Organizations: Organizational Characteristics Associated with Performance
88. Gill, B. S.; Beriwal, S.; Rajagopalan, M. S.; Wang, H.; Hodges, K.; Greenberger, J. S. (2015). Quantitative evaluation of radiation oncologists' adaptability to lower reimbursing treatment programs
89. Mc, Williams Jm; Hatfield, L. A.; Landon, B. E.; Chernew, M. E. (2020). Savings or Selection? Initial Spending Reductions in the Medicare Shared Savings Program and Considerations for Reform
90. DeLia, D.; Hoover, D.; Cantor, J. C. (2012). Statistical uncertainty in the Medicare shared savings program
91. Kim, Y.; Thirukumaran, C.; Temkin-Greener, H.; Hill, E.; Holloway, R.; Li, Y. (2021). The Effect of Medicare Shared Savings Program on Readmissions and Variations by Race/Ethnicity and Payer Status (December 9, 2020)
92. Murray, G. F.; D'Aunno, T.; Lewis, V. A. (2018). Trust, Money, and Power: Life Cycle Dynamics in Alliances Between Management Partners and Accountable Care Organizations
93. Kristensen, Søren Rud; Bech, Mickael; Lauridsen, Jørgen T. (2016). Who to pay for performance? The choice of organisational level for hospital performance incentives
94. Kristensen, Søren Rud; McDonald, Ruth; Sutton, Matt (2013). Should pay-for-performance schemes be locally designed? evidence from the commissioning for quality and innovation (CQUIN) framework
95. Hsu, J.; Vogeli, C.; Price, M.; Brand, R.; Chernew, M. E.; Mohta, N.; Chaguturu, S. K.; Weil, E.; Ferris, T. G. (2017). Substantial Physician Turnover And Beneficiary 'Churn' In A Large Medicare Pioneer ACO
96. Tanenbaum, J. E.; Votruba, M.; Einstadter, D.; Love, T. E.; Cebul, R. D. (2021). Adoption of Health System Innovations: Evidence of Urban-Rural Disparities from the Ohio Primary Care Marketplace
97. Murphy, W. S.; Siddiqi, A.; Cheng, T.; Lin, B.; Terry, D.; Talmo, C. T.; Murphy, S. B. (2019). 2018 John Charnley Award: Analysis of US Hip Replacement Bundled Payments: Physician-initiated Episodes Outperform Hospital-initiated Episodes
98. Goldman, L. Elizabeth; Henderson, Stuart; Dohan, Daniel P.; Talavera, Jason A.; Dudley, R. Adams (2007). Public Reporting and Pay-for-Performance: Safety-Net Hospital Executives' Concerns and Policy Suggestions
99. Truchil, A.; Dravid, N.; Singer, S.; Martinez, Z.; Kuruna, T.; Waulters, S. (2018). Lessons from the Camden Coalition of Healthcare Providers' First Medicaid Shared Savings Performance Evaluation
100. Zhao, M.; Hamadi, H.; Haley, D. R.; Xu, J.; White-Williams, C.; Park, S. (2020). Telehealth: Advances in Alternative Payment Models
101. Karina, Newhall; David, Stone; Ryan, Svoboda; Philip, Goodney (2016). Possible consequences of regionally based bundled payments for diabetic amputations for safety net hospitals in Texas
102. Friedberg, M. W.; Chen, P. G.; Simmons, M.; Sherry, T.; Mendel, P.; Raaen, L.; Ryan, J.; Orr, P.; Vargo, C.; Carlasare, L.; Botts, C.; Blake, K. (2020). Effects of Health Care Payment Models on Physician Practice in the United States: Follow-Up Study
103. Friedberg, M. W.; Chen, P. G.; White, C.; Jung, O.; Raaen, L.; Hirshman, S.; Hoch, E.; Stevens, C.; Ginsburg, P. B.; Casalino, L. P.; Tutty, M.; Vargo, C.; Lipinski, L. (2015). Effects of Health Care Payment Models on Physician Practice in the United States
104. Damberg, Cheryl L.; Elliott, Marc N.; Ewing, Brett A. (2015). Pay-For-Performance. Pay-For-Performance Schemes That Use Patient And Provider Categories Would Reduce Payment Disparities
105. George, M.; Bencic, S.; Bleiberg, S.; Alawa, N.; Sanghavi, D. (2014). Case study: Delivery and payment reform in congestive heart failure at two large academic centers
106. Kim, D. H.; Lloyd, C.; Fernandez, D. K.; Spielman, A.; Bradshaw, D. (2017). A Direct Experience in a New Accountable Care Organization: Results, Challenges, and the Role of the Neurosurgeon
107. Bertone MP, Lagarde M, Witter S. Performance-based financing in the context of the complex remuneration of health workers: findings from a mixed-method study in rural Sierra Leone. BMC Health Serv Res. 2016;16: 286.
108. Bleser WK, Saunders RS, Muhlestein DB, Morrison SQ, Pham HH, McClellan MB. ACO quality over time: the MSSP experience and opportunities for system-wide improvement. Am J Accountable Care. 2018;6(1):e1–15.
109. Campbell, S.M., Reeves, D., Kontopantelis, E., Sibbald, B., Roland, M.: Effects of pay for performance on the quality of primary care in England. N. Engl. J. Med. 361(4), 368–378 (2009)
110. Cheng SH, Lee TT, Chen CC. A longitudinal examination of a pay-for- performance program for diabetes care: evidence from a natural experiment. Med Care 2012; 50(2): 109–116.
111. Chimhutu V, Lindkvist I, Lange S: When incentives work too well: locally implemented pay for performance(P4P) and adverse sanctions towards home birth in Tanzania-a qualitative study. BMC Health Serv Res 2014, 14:23.
112. Colla CH, Lewis VA, Gottlieb DJ, Fisher ES. Cancer spending and accountable care organizations: evidence from the Physician Group Practice Demonstration. Healthc (Amst). 2013;1:100-107.
113. Colla CH, Lewis VA, Shortell SM, Fisher ES. First national survey of ACOs finds that physicians are playing strong leadership and ownership roles. Health Aff (Millwood). 2014; 33(6):964–971. [PubMed: 24889945]
114. Dummit LA, Kahvecioglu D, Marrufo G, et al. Association between hospital participation in a Medicare bundled payment initiative and payments and quality outcomes for lower extremity joint replacement episodes. JAMA. 2016;316(12): 1267-1278.
115. Ellimoottil C, Ryan AM, Hou H, Dupree J, Hallstrom B, Miller DC. Medicare’s new bundled payment for joint replacement may penalize hospitals that treat medically complex patients. Health Aff (Millwood). 2016;35:1651-1657.
116. Hollingsworth JM, Nallamothu BK, Yan P, et al. Medicare accountable care organizations are not associated with reductions in the use of low-value coronary revascularization. Circ Cardiovasc Qual Outcomes. 2018;11(6):e004492.
117. Hsu J, Price M, Vogeli C, et al. Bending The Spending Curve By Altering Care Delivery Patterns: The Role Of Care Management Within A Pioneer ACO. Health Aff (Project Hope). 2017;36(5):876-884.
118. Kalk A, Paul F, Grabosch E: ‘Paying for performance’ in Rwanda: does it pay off? Tropical Med Int Health 2010, 15(2):182–190.
119. Lewis, V. A., Colla, C. H., Carluzzo, K. L., Kler, S. E. and Fisher, E. S., Accountable Care Organizations in the United States: market and demographic factors associated with formation. Health Serv Res, 2013. 48(6 Pt 1): p. 1840-58.
120. Lewis, V.A., Tierney, K.I., Colla, C.H. and Shortell, S.M. (2017), “The new frontier of strategic alliances in health care: new partnerships under accountable care organizations”, Social Science and Medicine, Vol. 190, pp. 1-10, available at: https://doi.org/10.1016/j.socscimed.2017.04.054
121. McDonald R, Roland M. Pay for performance in primary care in England and California: comparison of unintended consequences. Ann Fam Med 2009; 7(2): 121–127.
122. McWilliams JM, Chernew ME, Landon BE, Schwartz AL. Performance differences in year 1 of pioneer accountable care organizations. N Engl J Med. 2015; 372(20):1927–1936. [PubMed: 25875195]
123. Muhlestein DB, Morrison SQ, Saunders RS, Bleser WK, McClellan MB, & Winfield LD (2018). Medicare Accountable Care Spending Patterns: Shifting Expenditures Associated with Savings. American Journal of Accountable Care, 6(1), 11–19.
124. Nyweide DJ, Lee W, Cuerdon TT, et al. Association of Pioneer Accountable Care Organizations vs traditional Medicare fee for service with spending, utilization, and patient experience. JAMA. 2015;313(21):2152-2161. doi: 10.1001/jama.2015.4930.
125. Ogundeji YK, Jackson C, Sheldon T, Olubajo O, Ihebuzor N. Pay for performance in Nigeria: the influence of context and implementation on results. Health Policy Plann. 2016;31:955–63.
126. Ridde V, Yaogo M, Zongo S, Somé P-A, Turcotte-Tremblay A-M. Twelve months of implementation of health care performance-based financing in Burkina Faso: a qualitative multiple case study. Int J Health Plann Manage. 2018;33:e153–67.
127. Ssengooba F, McPake B, Palmer N. Why performance-based contracting failed in Uganda--an “open-box” evaluation of a complex health system intervention. Soc Sci Med. 2012;75:377–83.
128. Sutherland, S. E., Egan, B. M., Fleming, D. O., Helmrich, G. A., Davis, R. A., Rutledge, V., & Sinopoli, A. (2016). Medicare Shared Savings Program second-year results: Predictors of success. GHS Proceedings, 1(1), 22–27.
129. Werner RM, Kolstad JT, Stuart EA, Polsky D. The effect of pay-for-performance in hospitals: lessons for quality improvement. Health Aff (Millwood) 2011; 30:690-8.
130. Winblad U, Mor V, McHugh JP, & Rahman M (2017). ACO-Affiliated Hospitals Reduced Rehospitalizations From Skilled Nursing Facilities Faster Than Other Hospitals. Health Aff (Millwood), 36(1), 67–73. doi:10.1377/hlthaff.2016.0759 [PubMed: 28069848]
131. Barbash IJ, Pike F, Gunn SR, Seymour CW, Kahn JM. Effects of physician-targeted pay for performance on use of spontaneous breathing trials in mechanically ventilated patients. Am J Respir Crit Care Med. 2017;196:56–63. https://doi.org/10.1164/rccm.201607-1505OC.
132. Chen H-J, Huang N, Chen L-S, Chou Y-J, Li C-P, Wu C-Y, et al. Does pay-for-performance program increase providers adherence to guidelines for managing hepatitis B and hepatitis C virus infection in Taiwan? PLoS One. 2016;11:e0161002. https://doi.org/10.1371/journal. pone.0161002.
133. Chen T-T. Hsueh Y-S (Arthur), Ko C-H, Shih L-N, Yang S-S. the effect of a hepatitis pay-for-performance program on outcomes of patients undergoing antiviral therapy. Eur J Pub Health. 2017;27:955–60. https://doi. org/10.1093/eurpub/ckx114.
134. Chen, T.T., Chung, K.P., Lin, I.C., Lai, M.S.: The unintended consequence of diabetes mellitus pay-for-performance (P4P) program in Taiwan: are patients with more comorbidities or more severe conditions likely to be excluded from the P4P program? Health Serv. Res. (28 Sept 2010)
135. Girault A, Bellanger M, Lalloué B, Loirat P, Moisdon JC, Minvielle E. Implementing hospital pay-for-performance: Lessons learned from the French pilot program. Health Policy 2017; 121: 407-417 [PMID: 28189271 DOI: 10.1016/j.healthpol.2017.01.007]
136. Haarsager J, Krishnasamy R, Gray NA. Impact of pay for performance on access at first dialysis in Queensland. Nephrology. 2018;23:469–75.
137. Hsieh H-M, Lin T-H, Lee I-C, Huang C-J, Shin S-J, Chiu H-C. The association between participation in a pay-for-performance program and macrovascular complications in patients with type 2 diabetes in Taiwan: a nationwide population-based cohort study. Prev Med. 2016;85:53–9. https://doi.org/10. 1016/j.ypmed.2015.12.013.
138. Jan CF, Lee MC, Chiu CM, Huang CK, Hwang SJ, Chang CJ, Chiu TY. Awareness of, attitude toward, and willingness to participate in pay for performance programs among family physicians: a cross-sectional study. BMC Fam Pract 2020; 21: 60 [PMID: 32228473 DOI: 10.1186/s12875-020-01118-9]
139. Kuo RNC, Chung KP, Lai MS. Effect of the Pay-for-Performance Program for Breast Cancer Care in Taiwan. J Oncol Pract. 2011;7(3S):e8s–15s.
140. Marsteller JA, Young JH, Fakeye OA, et al. Early provider perspectives within an accountable care organization. Am J Accountable Care 2016:4:27–37.
141. McConnell KJ, Renfro S, Chan BK, et al. Early Performance in Medicaid Accountable Care Organizations: A Comparison of Oregon and Colorado. JAMA Intern Med. 2017;177(4):538-545.
142. Roberts ET, Zaslavsky AM, McWilliams JM. The Value-Based Payment Modifier: Program Outcomes and Implications for Disparities. Ann Intern Med. 2018 Feb 20;168(4):255-65.
143. Whitcomb WF, Lagu T, Krushell RJ, Lehman AP, Greenbaum J, McGirr J, et al. Experience with designing and implementing a bundled payment program for total hip replacement. Jt Comm J Qual Patient Saf 2015;41:406-13.
